# Highly Sensitive, Easy-to-Use, One-Step Detection of Peroxide-, Nitrate- and Chlorate-Based Explosives with Electron-Rich Ni Porphyrins

**DOI:** 10.1021/jacs.3c14118

**Published:** 2024-05-01

**Authors:** Mike Brockmann, Gabriel Glotz, Jan-Simon von Glasenapp, Lara Unterriker, Dmytro Neshchadin, Georg Gescheidt, Rainer Herges

**Affiliations:** † Otto Diels-Institute of Organic Chemistry, Christian-Albrechts-Universität zu Kiel, Otto Hahn Platz 4, 24118 Kiel, Germany; ‡ Institute of Physical and Theoretical Chemistry, Graz University of Technology, Stremayrgasse 9, 8010 Graz, Austria

## Abstract

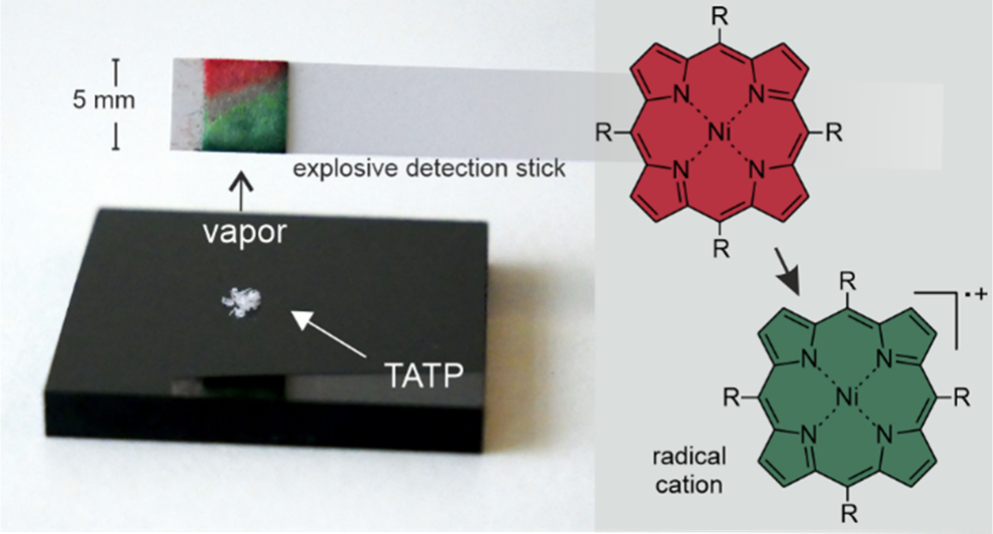

Homemade explosives, such as peroxides, nitrates, and chlorates, are increasingly abused by terrorists, criminals, and amateur chemists. The starting materials are easily accessible and instructions on how to make the explosives are described on the Internet. Safety considerations raise the need to detect these substances quickly and in low concentrations using simple methods. Conventional methods for the detection of these substances require sophisticated, electrically operated, analytical equipment. The simpler chemical detection methods are multistep and require several chemicals. We have developed a simple, one-step method that works similarly to a pH test strip in terms of handling. The analytical reaction is based on an acid-catalyzed oxidation of an electron-rich porphyrin to an unusually stable radical cation and dication. The detection limit for the peroxide-based explosive triacetone triperoxide (TATP), which is very frequently used by terrorists, is 40 ng and thus low enough to detect the substance without direct contact via the gas phase. It is sufficient to bring the stick close to the substance to observe a color change from red to green. Nitrates and chlorates, such as ammonium nitrate, urea nitrate, or potassium chlorate, are detected by direct contact with a sensitivity of 85–350 ng. A color change from red to dark brown is observed. The test thus detects all homemade explosives and distinguishes between the extremely impact-, shock-, and friction-sensitive peroxides and the less sensitive nitrates and chlorates by color change of a simple test strip.

## Introduction

Since the 1970s, there has been an increasing number of terrorist attacks with improvised explosive devices (IEDs) containing easily accessible explosives, such as peroxides, nitrates, and chlorates.^1,2^ As a countermeasure to protect sensitive areas, new analytical methods for trace detection of these substances have been developed and existing methods have been improved.^3,4^ In places with the appropriate infrastructure, sufficient space, and trained personnel, such as airports, instruments like gas chromatography–mass spectrometry (GC–MS), and especially ion mobility spectrometers (IMSs), have been introduced.^5^ However, there is also an increasing need for portable, easy-to-use, and fast methods to detect explosives onsite and for postblast analysis.^6^ A typical scenario is the discovery of an illegal laboratory with unknown substances, where the first responders must first quickly and reliably determine the safety situation.

Other applications include mass testing at public events or custom inspections and postblast scene analytics that are routinely performed to detect traces of remaining explosives.

**Figure 1:**
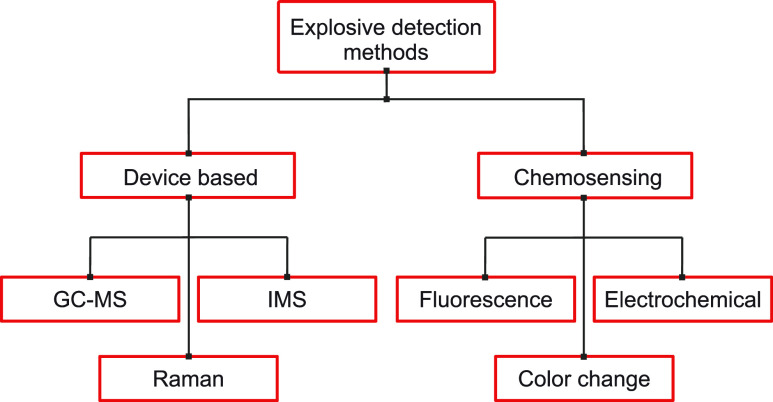
Overview of analytical methods for trace detection of explosives.

**Scheme 1:**
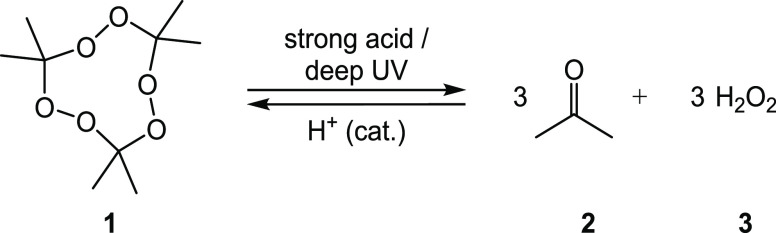
Acidic Decomposition and Acid-Catalyzed Preparation of TATP **1**

Chemosensing methods to detect peroxide-based explosives, nitrates, and chlorates include fluorescence response and colorimetric methods. Fluorescence detectors are less expensive than methods based on mass spectrometry, however, still require sensitive electronics and power supply.^5,7−10^ In general, the simplest and most robust detection methods are those based on a color change visible to the naked eye, e.g., test strips (Figure 1).^11^ Test strips for the detection of peroxides can unfortunately not be used for the detection of dialkyl peroxides. The cyclic alkyl peroxides to which the explosives TATP **1** (triacetone triperoxide), diacetone diperoxide, and HMTD **4** (hexamethylene triperoxide diamine) belong are remarkably chemically stable. All methods known to date for the detection of these explosives are at least two-step. The cyclic peroxide must first be split into hydroperoxides or hydrogen peroxide by a strong acid (p*K*_a_ < 1) or short-wave UV light (Scheme 1).^12−15^

However, no peroxide detection method has been available, which withstands the highly acidic conditions needed to hydrolyze the cyclic peroxides. Therefore, the reaction mixture must be neutralized before a conventional peroxide test is performed. Hence, three stages are needed to detect peroxide-based explosives:

1. Acid hydrolysis
2. Neutralization
3. Redox dye, color change

An exception is the colorimetric sensor developed by Suslick et al. TATP **1** is hydrolyzed in the vapor phase over a strongly acidic ion-exchange resin and the products are detected by colorimetric methods.^12^ Using a colorimetric sensor array, the approach discriminates between TATP and other peroxides and oxidants. Unfortunately, this method can only be applied to peroxides with high vapor pressure, such as TATP **1** (6.95 Pa at 25 °C)^16,17^ and deoxyadenosine diphosphate (DADP, 17.7 Pa),^18^ but not to HMTD **4**, which is considerably less volatile (3.9 × 10^–2^ Pa).^19^

To simplify existing detection methods, we set out to develop a sensitive, redox-responsive dye, which is stable under acidic conditions. This would allow us to mix the redox dye with the hydrolyzing acid to perform the detection in one step.

## Results and Discussion

### Electrochemistry of Metal Porphyrins

Among the chemically most persistent redox-active dyes are porphyrins. Nickel porphyrins only decompose (or demetallate) under very strongly acidic conditions.^20^

It is known that nickel porphyrins can be oxidized to the corresponding radical cations by one-electron oxidation. The electron transfer is accompanied by a color change from red to green.^21−23^ Another advantage of porphyrins, particularly Ni porphyrins is the extremely large molar extinction coefficient in the visible region (Soret band ε > 250,000 mol^–1^ dm^3^ cm^–1^), which should allow us to detect color changes at very low concentrations with the naked eye. Moreover, the human eye has a peak sensitivity at 555 nm (green light), approximately 10 times higher than that at 470 nm (blue) and 650 nm (red). Thus, a color change to green is particularly easy to recognize.

Ni porphyrins have been oxidized to the corresponding radical cation electrochemically and with a number of oxidizing agents, such as chromate or chlorate,^24^ however, not with TATP **1** or other cyclic or dialkyl peroxides.

**Figure 2:**
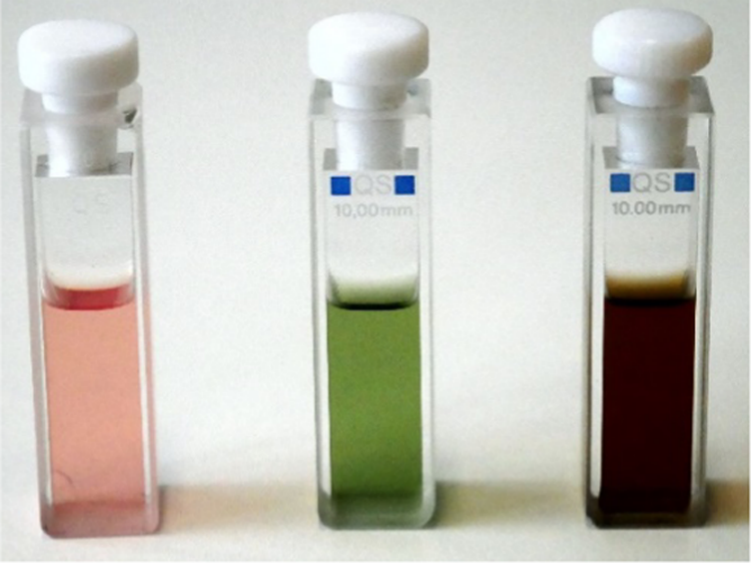
Porphyrin **9** was dissolved in dichloromethane (DCM, 25 μM, left). After addition of TFA, TATP **1** was added and the solution turned green (middle). Addition of nitrates (NO_3_^–^) leads to a brown color (right; for details, see the Detection of Nitrates section).

In preliminary experiments, we added TATP **1** to a solution of Ni-porphyrin **9** and trifluoroacetic acid (TFA) in dichloromethane and observed a color change from red to green (Figure 2).

The green species was identified as the radical cation (**9**^•+^; see further below). To develop this reaction further toward a generally applicable test for TATP **1** and other peroxide-based explosives, we systematically improved the response time and sensitivity of the system by determining the oxidation potentials and the reaction rate constants. As a straightforward strategy toward this end, we systematically changed the electronic properties of the porphyrin system by changing the substituents at the aryl group in the *meso* position from electron-withdrawing to electron-donating. In addition, we have changed the metal ion.

First, we have established the oxidation potentials of porphyrin derivatives **5**–**11** and their dependence on the presence of TFA and investigated the corresponding changes in the absorption spectra. Based on our observations, we then selected the most promising compounds for further evaluation by NMR.

Table 1 summarizes the oxidation potentials of **5**–**11** and quantifies their shifts upon addition of TFA (0.1 and 1 M) to the solutions used for the cyclic voltammetry (CV, CH_2_Cl_2_/0.1 M TBAClO_4_) measurements.

**Table 1:** Oxidation Potentials of Porphyrins **5**–**11** (0.1 mM) in DCM Containing 0.1 M TBAClO_4_ and TFA (0, 0.1, and 1.0 M) with a 100 mV s^–1^ Scan Rate^a^

	Ox(1)/V vs Fc/Fc^+^			Ox(2)/V vs Fc/Fc^+^		
	parent	0.1 M TFA	1 M TFA	parent	0.1 M TFA	1 M TFA
**5**	*0.93*^b^	0.67	0.67	0.93^b^	0.8	
**6**	*0.58*	*0.56*	*0.07*	*0.71*	*0.73*	*0.39*
**7**	0.53	0.49	c	0.54^d^	0.80	
**8**	*0.49*^e^	*0.48*	*0.05*	*0.59*	*0.69*	*0.37*
***9***	*0.58*^e^	*0.60*	*0.33*	*0.58*	*0.75*	*0.52*
**10**	0.46	c	c	0.67	c	c
**11**	0.65	0.43	c	0.66	c	c

a Reversible or quasi-reversible potentials are given in italics.

b Ref (25)(25).

c Not detectable.

d Weak shoulder.

e Ref (26).

For **5**, a two-electron transfer at 0.93 V vs Fc/Fc^+^ was reported.^25^ Upon addition of TFA (0.1 M, 1000 equiv), one wave at 0.67 V vs Fc/Fc^+^ and an additional weak peak at ca. 0.8 V vs. Fc/Fc^+^ were detected (Figure S25). Ni-porphyrin **6** reveals two oxidation waves at 0.58 (Ox(1)) and 0.71 (Ox(2)) V vs Fc/Fc^+^, in good agreement with previously reported values (Figure S26).^25,27^ The addition of TFA (0.1 M, 1000 equiv) hardly had any influence on the oxidation potentials (Figure S27). At a high TFA concentration (1 M), Ox(1) is drastically reduced to 0.07 V, whereas Ox(2) drops to 0.39 V vs Fc/Fc^+^ (Figure S28). Mesityl-substituted **7** shows two hardly distinguishable (second oxidation occurs as a shoulder) oxidations at 0.53/0.54 vs Fc/Fc^+^. Addition of TFA (0.1 M, 1000 equiv) leads to a more pronounced splitting of the two oxidations (Ox(1) to 0.49 and Ox (2) to 0.80 V vs Fc/Fc^+^). With 1 M TFA, **7** revealed no discernible oxidation waves (Figures S29 and S30). With para OMe groups at the phenyl substituents, **8** is probably the electron-richest porphyrin. As expected, Ox(1) with 0.49 V and Ox(2) with 0.59 V vs Fc/Fc^+^ are relatively low. With 0.1 M TFA, Ox(1) is unaffected (0.49 V), whereas Ox(2) is increased by 100 mV to 0.69 V vs Fc/Fc^+^ (Figures S31–S33). Analogous to **6**, 1 M TFA moves both oxidation potentials toward far less positive values of 0.05 and 0.37 V vs Fc/Fc^+^ V. 3,4,5-Methoxy substitution at the phenyl groups **9** lead to Ox(1)/Ox(2) of 0.56 V vs Fc/Fc^+^ as a two-electron process in line with published data.^26^ Low amounts of TFA do not affect Ox(1) and Ox(2) substantially (−>0.60 and 0.75 V vs Fc/Fc^+^, respectively), but as for **6** and **8** 1 M TFA leads to severe drops to 0.33 and 0.52 V vs Fc/Fc^+^, respectively (Figures S34–S36). The CV curves of Cu-porphyrin **10** indicate decomposition upon oxidation (**10**) (Figure S39). In the case of Pd-porphyrin **11**, a two-electron transfer (0.59 V vs Fc/Fc^+^) is detected without the addition of TFA, but its presence leads to its decomposition (Figure S40).

**Figure 3:**
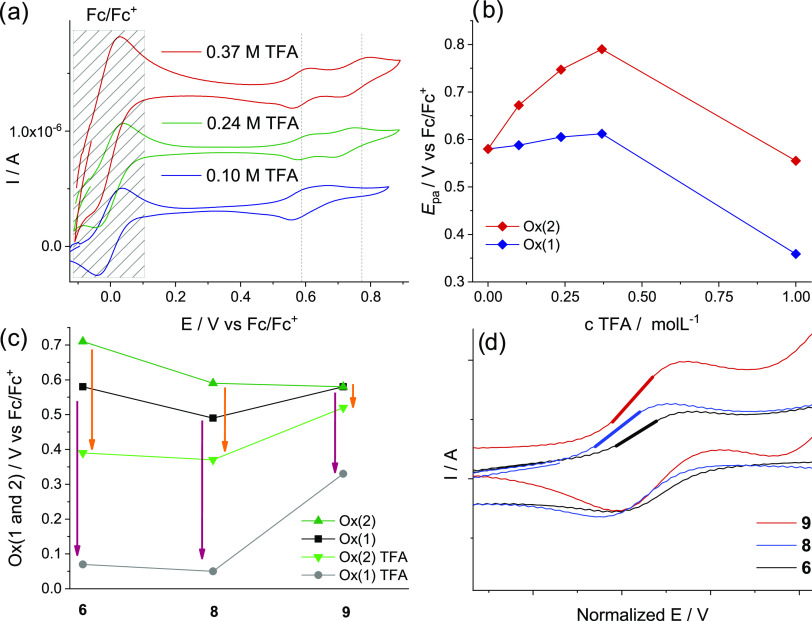
(a) Cyclovoltammograms of **9** at increasing concentration of TFA; (b) Shifts of Ox(1) and Ox(2) for **9** upon addition of TFA. (c) Drop of the oxidation potentials (Ox(1) and Ox(2)) for **6**, **8**, and **9** upon addition of 1 M TFA. (d) Overlay of the CV waves, representing Ox(1) for **6**, **8**, and **9**, indicating the different slopes for their first oxidation.

These results show that a high TFA concentration causes a substantial decrease of the oxidation potentials Ox(1) and Ox(2). The values are in line with the π system of the porphyrin ligand carrying the extra charges, reflecting the successive formation of a π-type radical cation and dication.^28^ Low concentrations of TFA lead to a slight increase of Ox(1), whereas a TFA concentration of 1 M, making it the dominating component of the solution, is the “game changer here” (see Figure 3b). This would be in line with findings that concentrated TFA particularly stabilizes π-type radical cations.^29,30^ Here, we have performed control experiments, in which an excess of tetraethylammonium trifluoroacetate (TEATFA) was added to the solution used for the electrochemical experiments. We have observed that the presence of TEATFA leads to distinctly different voltammograms than those taken in the presence of TFA (Figures S41 and S42). Accordingly, the stabilizing effect of TFA has to be ascribed to the acidic properties of the perfluorinated acid.

The drop of the oxidation potential upon addition of a very large excess of TFA is particularly significant for the first oxidation (Ox(1)). In terms of the size of this effect and reversible redox properties (Figure 3a), derivatives **6**, **8**, and **9** are promising candidates for well-reproducible redox reactions and sufficiently low oxidation potentials to achieve a favorable conversion to the characteristically colored radical cations (Figure 3c). Although, in this series, **9** has the highest Ox(1) value (0.33 vs 0.07 and 0.05 V vs Fc/Fc^+^for **6** and **8**), and the slope for its first oxidation is steepest. Accordingly, it can be expected that **9**-based sensors should have the fastest response. This is indeed by far the case in its application (see below). Therefore, it is the most preferable candidate for our purpose. Moreover, it is much better soluble in the formulation compared with **6** and **8**.

Accordingly, the following detailed investigations in terms of mechanism and response were carried out with **9**.

**Scheme 2:**
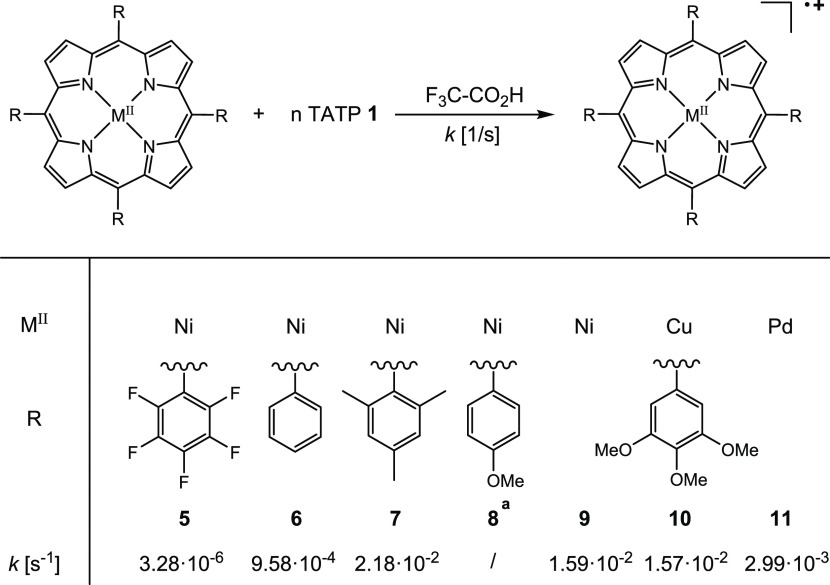
Pseudo-first-order Rate Constants *k* [s^–1^] of the Oxidation of Different Substituted Porphyrins Determined by UV/Vis (for Definition of the Reaction Rate Constant *k*, See Figures S1–S6) Due to poor solubility, determintation of the rate constant *k* was not possible.

As mentioned above, for practical applications, not only the oxidation potentials but also kinetic data (response time) are important. Therefore, we measured the reaction rates of porphyrins **5**–**11** in dry CH_2_Cl_2_ and a very large molar excess of TFA with a 10 M excess of TATP **1** (Figures S1–S6). The pseudo-first-order rate constants are listed in Scheme 2.

As expected, the lowest rate constant was observed for the electron-poor porphyrin **5** (Ni-TPPF_20_), which also has the highest oxidation potentials. Based on Hammett substituent parameters, *p*-MeO-porphyrin **8** should be the most electron-rich and indeed has the lowest oxidation potentials. If thermodynamic and kinetic parameters correlate, **8** should also exhibit the highest rate of oxidation. Unfortunately, porphyrin **8** is almost completely unsoluble in organic solvents and acids. Correlations between thermodynamic and kinetic data for all other porphyrins are less systematic. The fastest oxidation rates are measured for porphyrins **7** and **9**. However, the solubility of **7** in highly concentrated TFA is low and the second oxidation is not observed, which is in agreement with the electrochemical data. Porphyrin **9** exhibits higher oxidation potentials, probably because *m*-MeO in contrast to *p*-MeO substituents are electron-withdrawing. It is readily soluble in organic solvents, TFA, and neat perfluoropentanoic acid, and moreover, it reacts fast and exhibits a second oxidation to form the corresponding brown-colored dication (see the Detection of Nitrates section). We also investigated the Cu and Pd porphyrins **10** and **11**. Both porphyrins decompose under highly acidic conditions and are therefore not suitable as redox sensors.

In summary, there is no straightforward correlation between Hammett parameters or oxidation potentials and the rate of oxidation (*k*). Kadish et al. have shown in a detailed study that besides electronic effects of the meso-substituents, structural properties and especially the out-of-plane deformation of the porphyin scaffold have an important influence on the electrochemistry of Ni porphyrins.^31^ This finding also obviously applies to our study. Thermodynamical studies (oxidation potentials), kinetic investigations (rate of oxidation), and practical aspects (solubility) clearly indicate that [5,10,15,20-tetrakis(3,4,5-trimethoxyphenyl)porphyrinato]nickel(II) (**9**) in a strong acid is the most promising candidate to develop a detection method, particularly for peroxide-based exlosives.

In view of the development of a mild and efficient detection method, we systematically screened different acids with different p*K*_a_ values (Table 2).

**Table 2:** Detection of TATP **1** with Ni-porphyrin **9** in DCM in the Presence of Different Acids

acid	p*K*_a_	color change
TFA	0.23^32^	**+**
formic acid	3.75^33^	–
acetic acid	4.76^32^	–
methanesulfonic acid	–1.90^34^	–a
fluorosulfonic acid	–6.40^35^	–a
hydrochloric acid	–7.00^33^	–a
sulfuric acid	–3.00^33^	–a
*p*-toluenesulfonic acid	–2.80^35^	–b
boron trifluoride etherate		–
aluminum(III) chloride		–
titanium(IV) chloride		–a
pentafluoropropionic acid	0.18^36^	**+**
heptafluorobutyric acid	0.40^36^	**+**
perfluoropentanoic acid	–0.06^37^	**+**
trichloroacetic acid	0.51^33^	**+**

a Decomposition of porphyrin **9**.

b Very low solubility in dichloromethane.

It can be inferred from the results listed in Table 2 that the detection of TATP **1** by color change is observed only in the presence of acids that have a p*K*_a_ between 0 and 1. It should be noted that the p*K*_a_ values in Table 2 are only indicative because they have been measured in water. Acidities in organic solvents (DCM in the present case) could be different. Weaker acids, such as formic acid (p*K*_a_ 3.75), are not able to hydrolyze TATP **1**, and stronger acids, such as methanesulfonic acid (p*K*_a_ – 1.9), destroy the porphyrin **9**.

The choice of solvent is also important. The electrophilic nature of the porphyrin radical cation and the strongly acidic reaction conditions imply that only weakly nucleophilic solvents should be envisaged. Nevertheless, a high polarity is necessary to provide sufficient solubility of the porphyrin and TATP **1**. Furthermore, fluorinated and chlorinated solvents of high polarity stabilize carbocations and radical cations and thus favor the oxidation of the porphyrin (Table 3).

**Table 3:** Detection of TATP **1** with Porphyrin **9** in Different Solvents and a Large Excess of TFA^a^

solvent	color change	miscibility
DCM	**+**	**+**
1,1,2,2-tetrachloroethane	**+**	–
1,1,2-trichloroethane	**+**	**+**
hexafluoroisopropanol	**+**	**+**
1,2-dichloroethane	**+**	**+**
chloroform	**+**	**+**
nitromethane	**+**	**+**
1,2,3-trichloropropane	**+**	**+**
nitrobenzene	**+**	**+**
trifluoroacetic acid (TFA)	**+**	b
perfluoropentanoic acid	**+**	b
1,1,1,2,3-pentachloropropane	–	–
2,2,2-trifluoroethanol	–	**+**
fluorobenzene	–	**+**
2,2,2-trichloroethanol	–	**+**
acetonitrile	–	**+**

a For general procedures and additional solvents, see the SI (Determination of different solvents for the detection of explosives and Table S1).

b Neat.

The data in Table 3 indicate that only very weakly nucleophilic solvents are suitable. Obviously, trifluoroethanol, trichloroethanol, and acetonitrile are too nucleophilic. The fastest and most sensitive response was observed in DCM/TFA, neat TFA, and neat perfluoropentanoic acid. For practical applications, neat perfluoropentanoic acid is ideal because of its low vapor pressure (bp 140 °C).

### Detection of Peroxides

To elucidate the mechanism of the color change reaction, we performed NMR, UV, and electron paramagnetic resonance (EPR) measurements. In preliminary NMR experiments, we investigated the acid-catalyzed decomposition of TATP **1**. A 970 μM solution of TATP **1** in CD_2_Cl_2_ was mixed with a 1000-fold excess of TFA at 25 °C. Within 3 min, half of the TATP **1** was converted to acetone and hydrogen peroxide. No intermediates, such as the ring-opened 2,2′-dihydroperoxy-2,2′-diisopropylperoxide or 2-hydroxy-2-propylhydroperoxide or DADP, were spectroscopically detected (Figure S13). In a further experiment, we followed the reaction in the presence of [5,10,15,20-tetrakis(3,4,5-trimethoxyphenyl)porphyrinato]nickel(II) (**9**). The ^1^H NMR spectrum of pure Ni(II)-porphyrin **9** exhibits four signals assigned to the pyrrole, *o*-phenyl, *m*-methoxy, and *p*-methoxy substituents (a). Upon addition of a very large excess (1000-fold with respect to **9**) of TFA, a line broadening of the methoxy signals is observed, which is due to reversible protonation (b) (for an enlarged view, see Figure S16). No paramagnetic signals (e.g., downfield shift or broadening of the pyrrole protons) are visible in the ^1^H NMR spectrum, which indicates that TFA does not axially coordinate to the nickel(II) ion (Figure S14).

**Figure 4:**
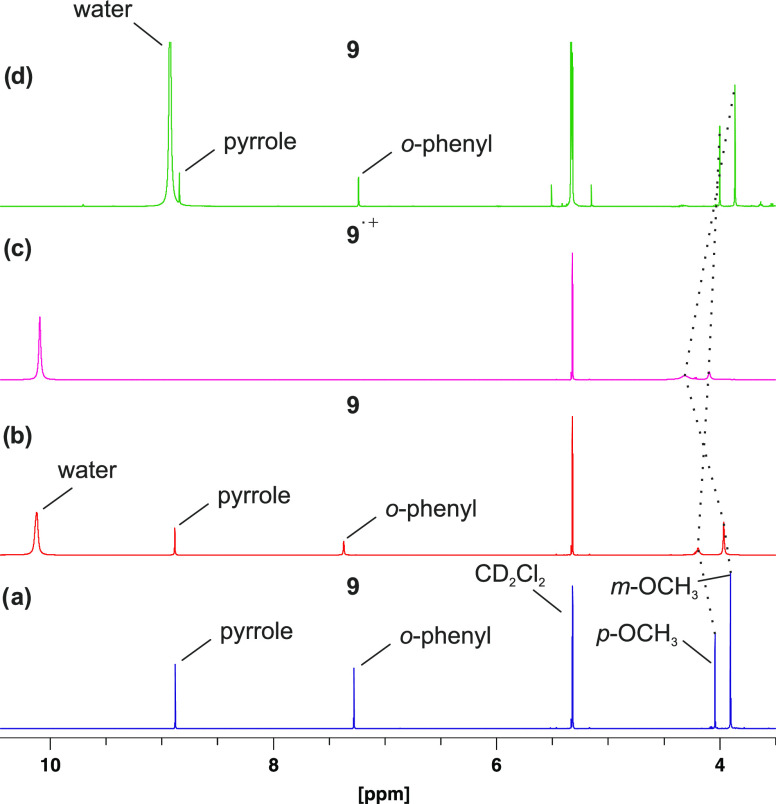
NMR spectra (600 MHz) of porphyrin **9** (a), after successive addition of TFA (b), TATP (c), and NEt_3_ (d). Almost no change in the spectrum is observed after adding TFA to porphyrin **9** (spectrum, b). Upon addition of TATP (**1**), the pyrrole and *o*-phenyl protons disappear, which is indicative of the formation of the radical cation **9**^•**+**^ (spectrum, c). Further addition of NEt_3_ restores the original spectrum of the neutral porphyrin **9** (spectrum, d). Only the region from 4 to 11 ppm is shown. The complete spectra are shown in the SI (Figure S15).

Upon addition of TATP **1** (1/6 equiv with respect to porphyrin **9**) to this solution, a rapid change of the ^1^H NMR spectrum was observed (c). As in the previous experiment, half of the TATP **1** was hydrolyzed to acetone and hydrogen peroxide within 3 min. Hence, the porphyrin has no catalytic effect on the decomposition of TATP **1**. However, the spectrum of the Ni-porphyrin **9** changes rapidly. After 3 min, the signals of the pyrrole protons and the *ortho* protons of the *meso*-phenyl substituents disappeared (**c**). This is in agreement with the formation of a π radical cation, which has the highest spin population at these positions, causing a paramagnetic broadening of the corresponding ^1^H NMR signals. Concurrently, the signals of the methoxy groups remain visible and shift in opposite directions. The *p*-methoxy signal shifts high field and the *m*-methoxy signal shifts downfield. Again, this is in agreement with the calculated positive and negative spin densities of the π radical cation at these protons. Even in a mixture of the neutral porphyrin **9** and the radical cation **9**^•+^, only one set of porphyrin signals is observed, shifting continuously as a function of the relative concentrations, which indicates a fast electron transfer between the neutral molecule and the π radical cation (**9**^•+^ + **9** → **9** + **9**^•+^) (Figure 4). After the addition of an excess of triethylamine, the *p*-methoxy signals shift downfield and the *m*-methoxy signals shift high field. In addition, the pyrrole and *o*-protons become visible again and the initial spectrum of porphyrin **9** is recovered (**d**). This implies the reduction of the π radical cation **9**^•+^ back to porphyrin **9** (Figure 4).

**Figure 5:**
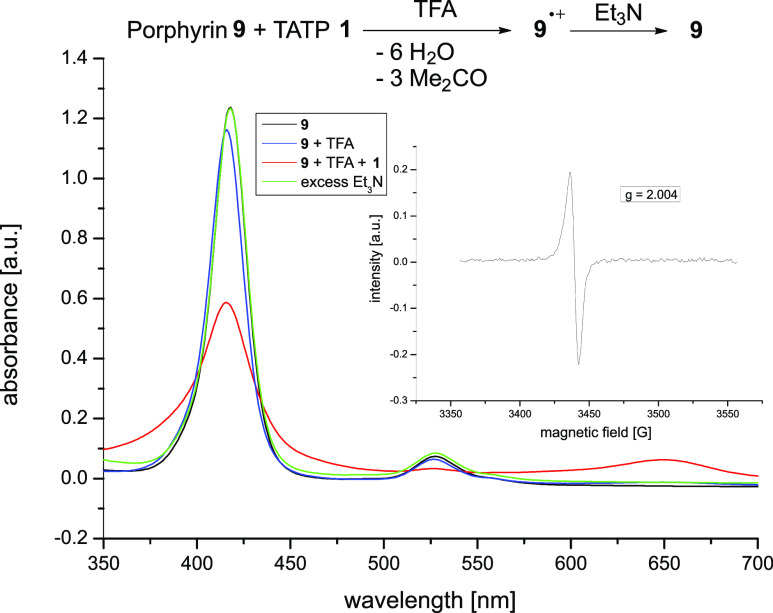
UV/vis spectrum of [5,10,15,20-tetrakis(3,4,5-trimethoxyphenyl)porphyrinato]nickel(II) (**9**) in dichloromethane (black) is shown. To this solution was successively added TFA (blue spectrum) and TATP **1** (red). To prove the reversibility of the reaction, Et_3_N was added (green). Note that the black curve of the original neutral porphyrin and the green curve of the neutral porphyrin restored from the radical cation almost perfectly overlap. The measurements were carried out in dichloromethane at 25 °C. Additionally, the EPR spectrum is shown, which was recorded at 77 K in DCM after addition of TFA and TATP **1** to porphyrin **9**.

We also followed the redox reaction by UV/vis spectroscopy in DCM and excess of TFA (Figure 5). Porphyrin **9** exhibits the typical spectrum of symmetrical porphyrins with a Soret band at λ_max_ = 418 nm (ε = 257,040 mol^–1^ dm^3^ cm^–1^) and a Q-band at λ_max_ = 524 nm (ε = 15,560 mol^–1^ dm^3^ cm^–1^). Upon addition of a 49,000-fold excess of TFA, a very small hypsochromic shift of the Soret band (2 nm) was observed. In agreement with our NMR measurements, this suggests that there is no axial coordination of TFA at the Ni ion since one would expect a bathochromic shift in this case.^38,39^ A demetalation and protonation at the pyrrole nitrogen atoms can also be excluded because this would as well lead to a bathochromic and not a hypsochromic shift (Figure S7). We interpret the slight hypsochromic shift as a protonation of the methoxy groups at the *meso*-phenyl rings.

After addition of TATP (1/6 equiv with respect to porphyrin **9**), the Soret band decreases, broadens, and shifts hypsochromically to λ_max_ = 416 nm (ε = 119,755 mol^–1^ dm^3^ cm^–1^). The Q-band at 524 nm disappears and a very broad band between 600 and 700 nm increases in intensity. The same was observed while performing potential-controlled oxidation of **9** in the presence of TFA (Figure S38). The spectrum has very close resemblance to known Ni-porphyrin radical cations, such as Ni-TPP^•+^ (**6**^•+^) or tetrakis(methoxyphenyl)-substituted Ni porphyrins (e.g., **8**^•**+**^).^21,23,26^ Moreover, the EPR spectrum exhibits a *g*-factor of *g* = 2.004 (Figure 5), which is close to the corresponding value of Ni-tetraphenyl porphyrin radical cation (*g* = 2.005)^40^ and [5,10,15,20-tetrakis(2,4,6-trimethoxyphenyl)porphyrinato]nickel(II) radical cation (*g* = 2.006).^26^ Addition of triethylamine, DABCO, or hydrazine restores the UV spectrum of the original neutral porphyrin **9** (Figure 5).

**Figure 6:**
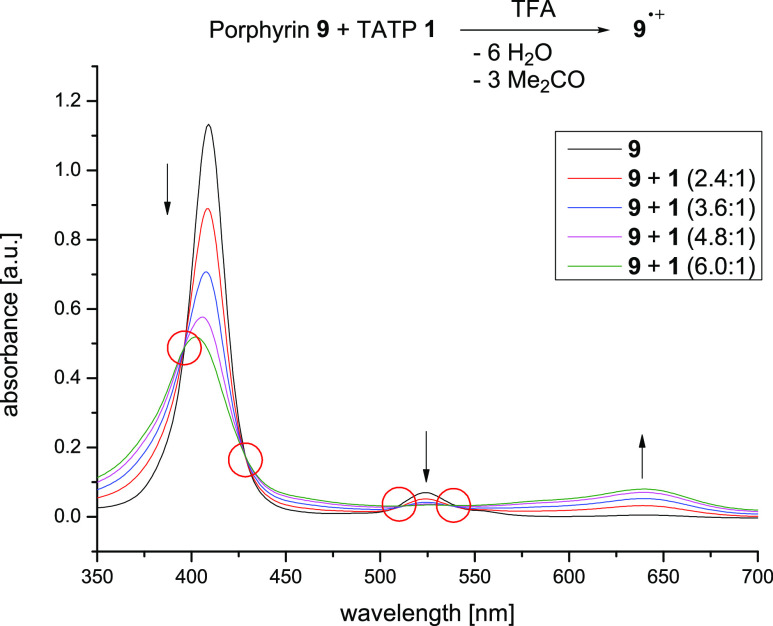
UV spectrum of porphyrin **9** and the spectra after addition of different amounts of TATP **1**. Up to a ratio of porphyrin **9**/TATP **1** of 6:1 isosbestic points are observed. The measurements were carried out in perfluoropentanoic acid at 25 °C.

UV titration experiments revealed the stoichiometry of the redox reaction. Upon addition of TATP **1**, the Q-band at 524 nm decreases and a broad band with λ_max_ = 639 nm increases in intensity. Up to a ratio (porphyrin **9**)/TATP **1** = 6:1, clear isosbestic points are observed, which indicates that only two species **9** and **9**^•+^ are present in solution (Figure 6).

In summary, one molecule of TATP **1** oxidizes 6 molecules of porphyrin **9** to the radical cation **9**^•+^, which explains the high sensitivity of the method.

**Scheme 3:**
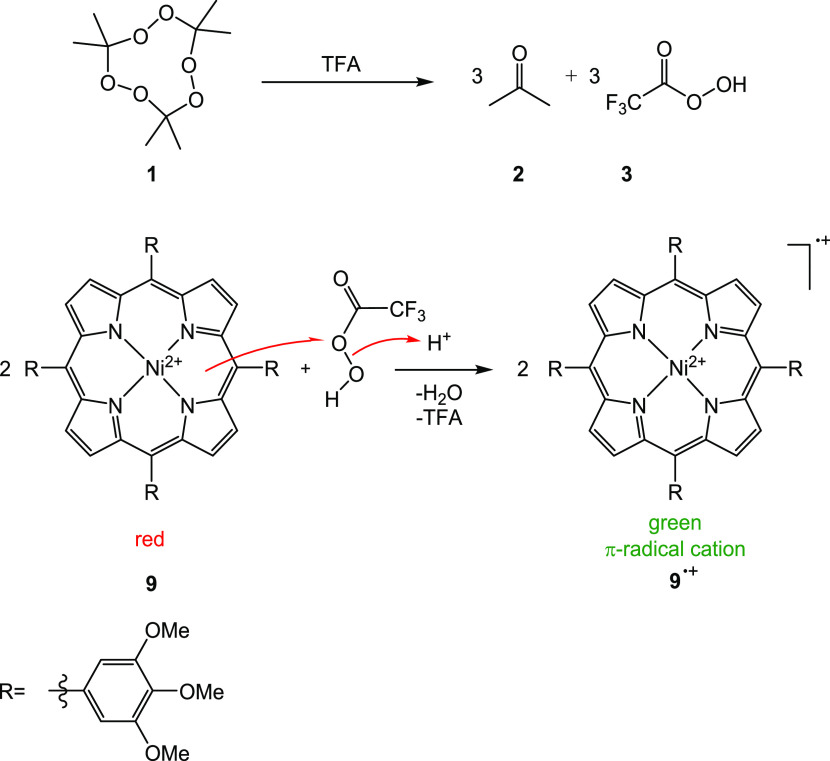
Proposed Mechanism for the Formation of the Radical Cation **9**^•+^ (For Detailed Mechanism, see SI Scheme S1)

Based on our experimental results, we propose the following mechanism (Scheme 3):

Our hypothetic mechanism includes the following steps: A proton-assisted single electron transfer from the Ni(II) porphyrin to the preformed peracid of TFA leads to the formation of the radical cation **9**^•**+**^ and a TFA radical. The TFA radical probably is in equilibrium with nickel oxo species^41−43^ (Scheme S1) or directly oxidizes a second Ni(II) porphyrin. Hence, one peracid TFA molecule oxidizes two Ni porphyrins, or one TATP **1** molecule oxidizes 6 Ni porphyrin molecules to the corresponding radical cation.

In addition to explosives, some natural products also have a cyclic peroxide structure, such as prostaglandin H_2_ and artemisinin.^44^ Both compounds give a positive test result, with artemisinin reacting very slowly (see SI “Detection of prostaglandin H_2_, artemisinin, other peroxides, and everyday hygiene products”). Other peroxides and everyday hygiene products were also tested (see Table S2).

### Detection of Nitrates

Besides peroxide-based compounds, such as TATP **1** and HMTD **4**, nitrate and chlorate salts are used to prepare homemade explosives (HMEs) and improvised explosive devices (IEDs). The extension of the present peroxide test to these components of HMEs therefore seemed worthwhile. The majority of colorimetric methods to detect nitrate are based on the preceding reduction of the chemically relatively inert nitrate to the more reactive nitrite ion.^45^ The latter can be detected with a number of reagents. Arguably, the most frequently used colorimetric method is the so-called Griess test.^46^ Nitrite (NO_2_^–^) reacts with an electron-deficient aniline to the corresponding diazonium salt, which reacts with an electron-rich aromatic compound to form a deeply colored azo dye (azo coupling).

Tetraaryl porphyrins are known to react with nitrogen dioxide (NO_2_) and nitrite (NO_2_^–^) to form intermediate porphyrin radical cations, which further react to nitro-substituted porphyrins.^47^ The reaction of Ni-tetraphenyl porphyrin **6** with nitric acid (HNO_3_), however, directly leads to the mono- and poly-nitrated porphyrins (β position) probably via electrophilic aromatic substitution.^48^ The latter reaction leads only to a very small color shift that is barely visible to the naked eye and is therefore not suitable as a detection method for nitrate. Surprisingly, the porphyrin **9**, in contrast to Ni-TPP **6**, reacted with nitrate under acidic conditions immediately and in one step, leading to a color change from red to deep brown.

To elucidate the mechanism of the color change reaction, NMR and UV measurements were performed.

**Figure 7:**
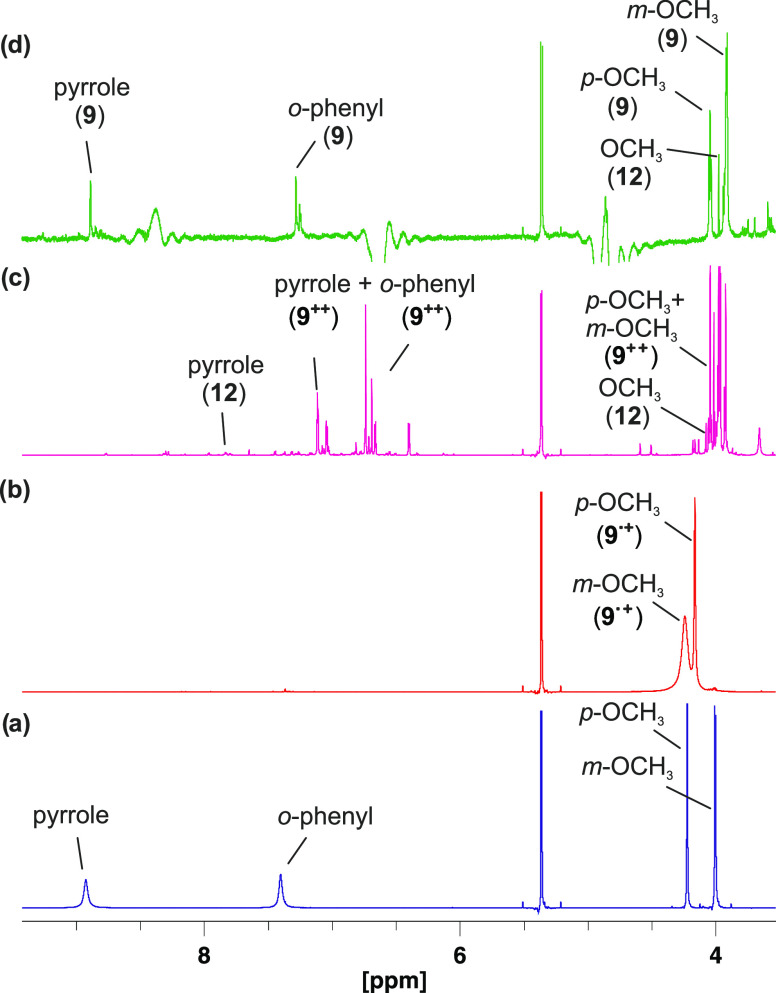
Change in NMR spectra (600 MHz) after addition of TFA and NH_4_NO_3_. (a, b) Upon addition of 0.2 equiv of NH_4_NO_3_ to a solution of **9** and TFA, the pyrrole and *o*-phenyl signals disappear and the *p*-methoxy protons shift high field and those of the *m*-methoxy protons shift low field, which is indicative of the formation of the radical cation **9**^•**+**^. (b, c) Further addition of NH_4_NO_3_ results in the formation of new asymmetric species, which is assigned to the dication **9**^**++**^. Furthermore, another unsymmetric species is forming, which was identified as a species nitrated in the β position 2-nitroporphyrin **12**. (c, d) After the addition of triethylamine, the initial spectrum of **9** is recovered and only the signals for the β nitrated porphyrin **12** remain. Only the region from 4 to 9.5 ppm is shown. The complete spectra are given in the SI (Figure S17). Note that the artifacts in spectrum (d) are due to the large excess of NEt_3_.

In an NMR titration experiment, we followed the reaction of porphyrin **9** with nitrate under acidic conditions. The ^1^H NMR spectrum of Ni(II)-porphyrin **9** after the addition of a very large excess of TFA (1000-fold with respect to **9**) exhibits four signals assigned to the pyrrole, *o*-phenyl, *m*-methoxy, and *p*-methoxy substituents (Figure 7a).

Upon addition of isotope-labeled NH_4_^15^NO_3_ (0.2 equiv with respect to porphyrin **9**) to this solution, a rapid change of the ^1^H NMR spectrum was observed (Figure 7b). The spectrum corresponds to the π radical cation **9**^•+^, which is confirmed by the disappearance of the signals of the pyrrole protons and the *ortho* protons of the *meso*-phenyl substituents. Concurrently, the signals of the methoxy groups remain visible and shift in opposite directions (Figure 7b, red spectrum). After the addition of another 0.8 equiv of NH_4_^15^NO_3_ (total 1.0 equiv with respect to porphyrin **9**), the signals for **9**^•+^ disappear completely and a species of low symmetry forms. Four doublets appear (in the range of 7.12–6.40 ppm), each with an integral of two protons corresponding to the high-field-shifted pyrrole protons. In the same region, there are also 4 singlets corresponding to the *ortho* protons of the phenyl substituents (Figure S18). The *para* and *meta* methoxy groups of the phenyl substituents also split into multiple signals (in the range of 4.00–3.90), confirming the formation of a structure of low symmetry (*C*_i_, *C*_2_, or *C*_s_), which could be identified as the dication **9**^**++**^ (Figure S18).

**Scheme 4:**
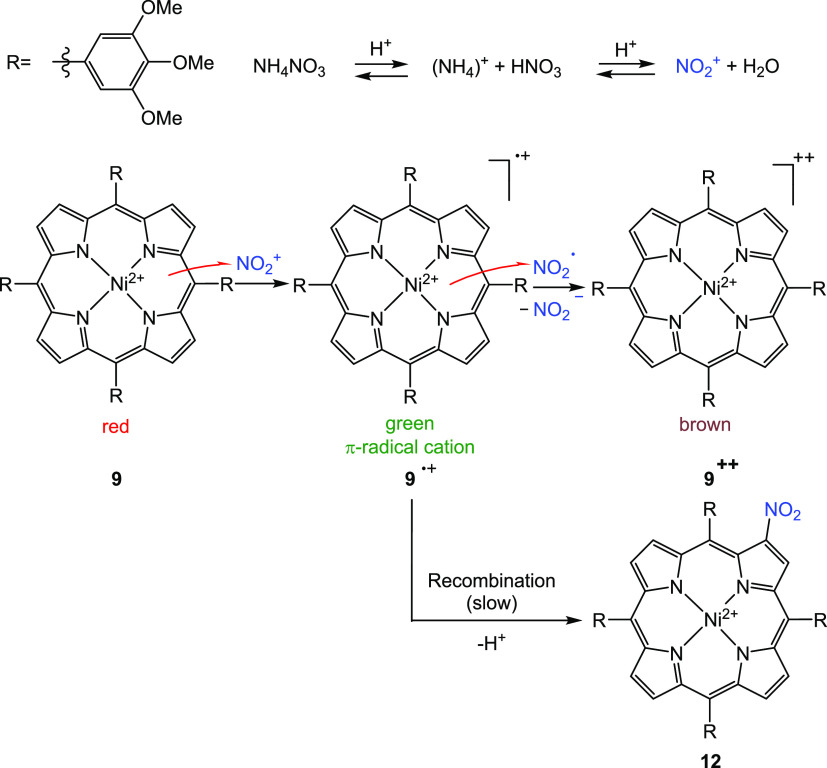
Proposed Reaction Mechanism for the Formation of the Porphyrin Dication **9**^+2^ and 2-Nitroporphyrin **12** The dication **9**^+2^ has a deep brown color (very broad absorption in the visible range, Figure 9), which explains the color change of the neutral porphyrin from red to deep brown in the presence of NO_3_^–^.

Another asymmetric species slowly forms with signals in the range of 9–7.5 ppm. ^15^N–^1^H HMBC measurements revealed that this species is the 2-nitroporphyrin **12** (Figure S19; Figure 7c, pink spectrum). The ^15^N signal couples with a singlet signal at 7.64 ppm (β proton). In addition, based on the two-dimensional (2D) spectra, six doublets for the other β protons with an integral of one and four singlets with an integral of two (*o*-methoxy protons) are visible. This indicates nitration in the β position (at one of the pyrrole rings) (Figure S20). The literature also agrees that Ni porphyrins usually react at the β position.^48^ Two different mechanisms are conceivable: a direct electrophilic aromatic substitution or a reaction via the π radical cation (Scheme 4).^47^ In our case, based on our results, it is more likely that nitration proceeds via the intermediate stage of the radical cation (Figures 7 and 9).

After the addition of an excess of triethylamine, the initial spectrum of **9** is recovered (Figure 7d, green spectrum). In the aromatic region and in the methoxy proton region, small signals are visible, which are assigned to the nitrated species **12**, whose formation is not reversible.

**Figure 8:**
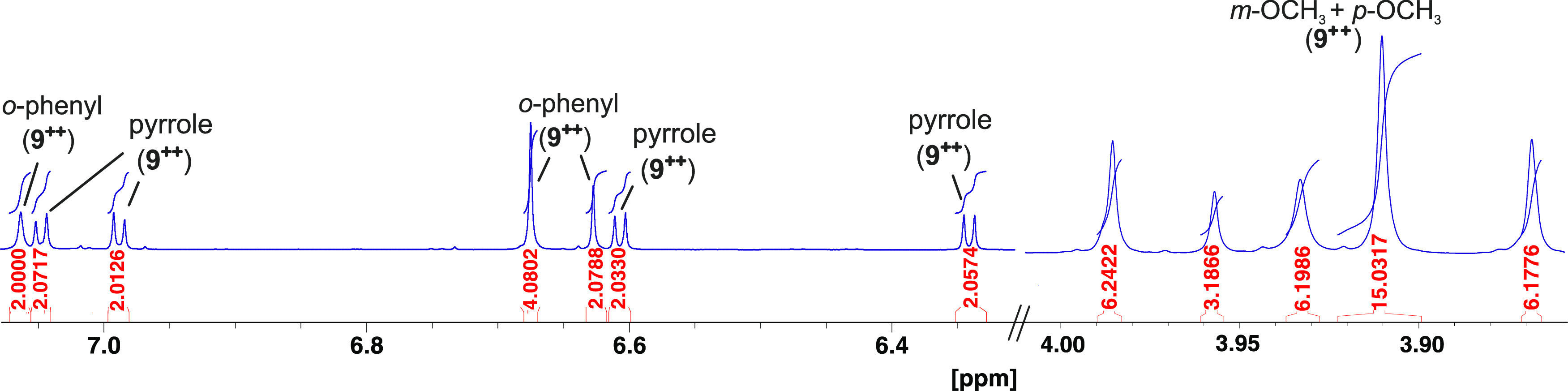
^1^H NMR spectrum (600 MHz) of dication **9**^**++**^ prepared with a large excess of TFA (49,700-fold) and Pb(OAc)_4_ (1 equiv with respect to porphyrin **9**). The complete spectra are given in the SI (Figure S21).

To avoid the formation of the nitrated species **12** and to obtain a clean reference spectrum of the dication **9**^**++**^, we used Pb(OAc)_4_ as a strong oxidizing agent. Under neutral conditions, no reaction took place. After addition of a large excess of TFA, the spectrum of the dication **9**^**++**^ was observed (Figure 8).

In the aromatic region, four doublets are observed, each with an integral of two protons (eight protons in total). These can be attributed to the pyrrole protons. In 2D experiments (COSY) (Figure S22), we found out that the pyrrole protons couple only with each other, suggesting that the protons bound to a pyrrole are chemically inequivalent, but there are two chemically equivalent pyrrole rings each. Additionally, we identify two singlets, each with an integral of two protons and one singlet with an integral of four protons (eight protons in total). These singlets can be associated with the *ortho* protons of the phenyl substituent.

In the aliphatic region, a total of five singlets are observed, with a combined integral of 36 protons. Nonetheless, despite employing 2D spectra (HSQC + HMBC) analysis (Figure S23), it remains challenging to achieve an unequivocal assignment for these protons.

The ^1^H and ^13^C shifts were also calculated using density functional theory (PBE/def2-SVP//TPSS/pcSseg-2). The experimental and calculated results are compared in Table 4. For computational details, see the Theoretical Calculations section.

**Table 4:** Comparison of the Experimental ^1^H Shifts of the Dication **9**^+2^ (*C*_2_ Symmetry) and the Calculated Shifts (for Detailed Information, see SI “Computational Details”)

numbering	experimental [ppm]	calculated^b^ [ppm]
*o*-phenyl-H	7.06 (2H)	6.54 (2H)
	6.68 (4H)	6.42 (2H)
	6.63 (2H)	6.33 (2H)
		6.22 (2H)
pyrrole-H	7.05 (2H)	7.21 (2H)
	6.99 (2H)	7.11 (2H)
	6.61 (2H)	6.60 (2H)
	6.34 (2H)	6.50 (2H)
*m*-methoxy-H	3.87–3.99	3.79–4.70^a^
*p*-methoxy-H	3.87–3.99	3.62–4.70^a^

a Note that there is almost free rotation of the methoxy groups, however, the calculations refer to a single conformation, including H-bonds.

b pbe/def2-SVP//M06-L/pcSseg-2.

The agreement of density functional theory (DFT) calculated and experimental ^1^H NMR shifts is within the usual range expected at this level of theory and without consideration of solvent effects.

The analogous calculation was performed for the shifts in the ^13^C spectrum (see Table 5). It should be noted that the experimental values are obtained by 2D spectra since the intensities of the signals are too low (Figure S23).

**Table 5:**
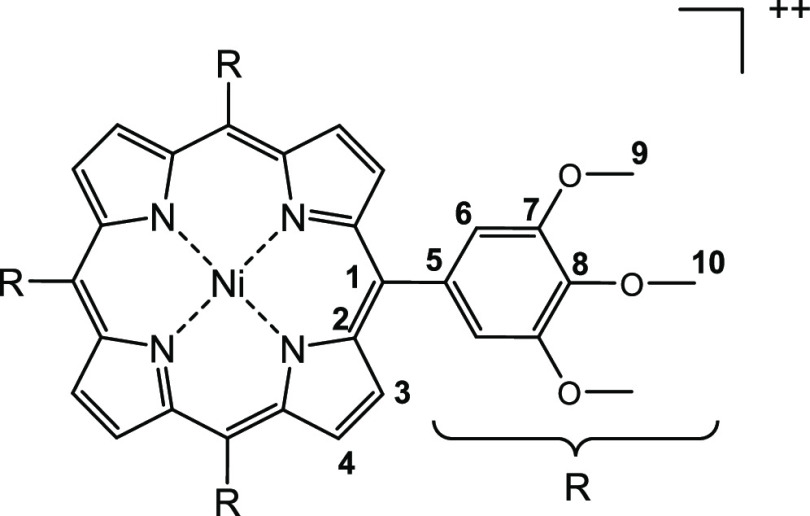
Comparison of the Experimental ^13^C Shifts of the Dication **9**^+2^ and the Calculated Values (for the Complete Set of ^13^C Shifts, See Table S12)

numbering	experimental [ppm]	calculated^b^ [ppm]
*C*-1^a^	148.2	147.0 (2C)
	145.3	146.7 (2C)
*C*-2^a^	156.7	155.9 (2C)
	154.9	155.6 (2C)
		151.8 (2C)
		151.7 (2C)
*C*-2^a^	137.6	138.1 (2C)
	135.6	137.7 (2C)
	132.0	134.2 (2C)
	131.1	133.3 (2C)

a Experimental assignment based on NMR spectra is ambiguous. Assignment is based on calculation results.

b PBE/def2-SVP//TPSS/pcSseg-2

Also, in this case, a comparison of the experimental and calculated shifts results in quite good agreement.

In the reaction **9** + Pb(OAc)_4_ → **9**^**++**^ + 2 OAc^–^ + Pb(OAc)_2_, no protons are transferred. We attribute the role of the large excess of TFA to the association of TFA with the porphyrin dication and the thermodynamic stabilization of **9**^**++**^ (for a ^1^H NMR spectrum of **9**^+2^, Figure S21).

The reaction of porphyrin **9** with nitrate was also investigated by UV spectroscopy. Upon addition of NH_4_NO_3_ again, the Q-band decreases and a broad band with λ_max_ = 639 nm increases in intensity, indicating the formation of the π radical cation **9**^•+^. Up to a ratio (porphyrin **9**)/NH_4_NO_3_ = 1:1, a clear isosbestic point is observed, which indicates that only two species **9** and **9**^•+^ are present in solution (Figure S8).

**Figure 9:**
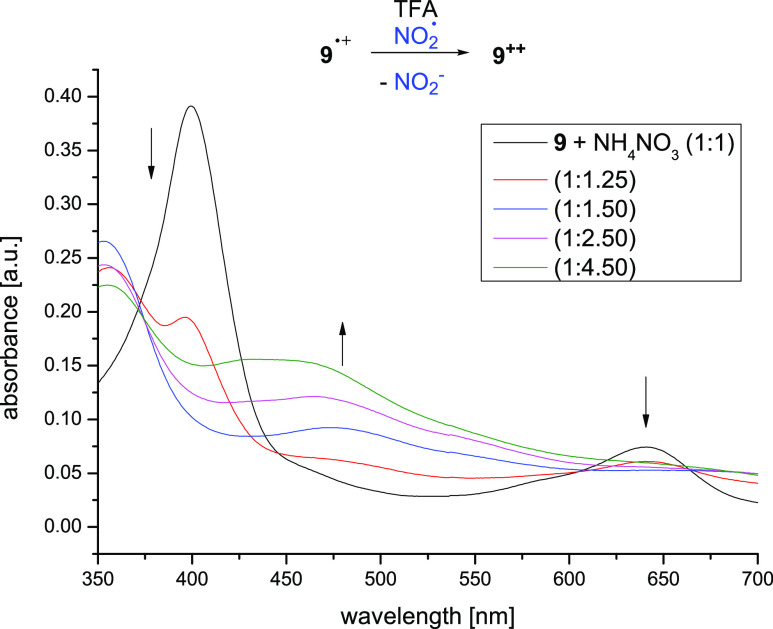
UV spectrum of porphyrin **9**^•**+**^ (black) and the spectra after addition of an excess of NH_4_NO_3_ (up to a ratio of 1:4.5). The typical spectrum of the dication **9**^**++**^ is forming. The measurements were carried out in perfluoropentanoic acid at 25 °C.

Further addition of NH_4_NO_3_ up to a ratio of 1:4.50 (based on porphyrin **9**) leads to a drastic change in the UV spectrum. The Soret band clearly loses intensity and shifts hypsochromic from λ_max_ = 409 nm to λ_max_ = 354 nm. There is also the formation of a broad shoulder in the range from 410 to 500 nm and the simultaneous decrease of the broad Q-band between 600–700 nm (Figure 9). This spectrum is typical for Ni porphyrin dications described in the literature.^21,26^ Simultaneously, with the oxidation of the π radical cation **9**^•+^ to the dication **9**^**++**^, small amounts of the β nitrated porphyrin **12** are formed. Therefore, no isosbestic point is visible (Figure 9).

Again, these observations were corroborated by a titration of **9** with Pb(OAc)_4_ in perfluoropentanoic acid. As expected, the typical spectrum for the π radical cation **9**^•+^ formed first, and upon further addition, the spectrum of the dication **9**^**++**^ was obtained (Figures S9 and S10).

Based on ^1^H NMR, ^15^N NMR, and UV–vis spectroscopy experiments, we propose the following mechanism (Scheme 4).

Under strongly acidic conditions (TFA or perfluoropentanoic acid), nitrate salts form the nitronium cation NO_2_^+^, which oxidizes the porphyrin **9** to the radical cation **9**^•**+**^ and is itself reduced to nitrogen dioxide (NO_2_^•^). Nitrogen dioxide in a fast reaction oxidizes the radical cation **9**^•**+**^ to the dication **9**^**++**^ and in parallel slowly recombines with the radical cation to form the nitrated porphyrin **12**.

The reaction of **9** and TFA with potassium chlorate also leads to a color change from red to brown. NMR and UV measurements reveal the intermediate formation of radical cation **9**^•**+**^ and subsequently of the dication **9**^**++**^ (Figures S11, S12 and S24).

### Theoretical Calculations

To elucidate the structures of the radical cation **9**^•**+**^ and the dication **9**^**++**^, quantum chemical geometry optimizations (PBE/def2-SVP) and NMR calculations (TPSS/pcSseg-2) were performed (for further information, see SI “Computational Details”).^49−53^ The chemical shifts from the ^1^H and ^13^C NMR calculations were compared with the experimental values to assist the assignment of the NMR signals to the corresponding hydrogen and carbon atoms (Tables 5 and 6). Toward this end **9**, **9**^•**+**^, and **9**^**++**^ were optimized at the PBE/def2-SVP level of density functional theory without symmetry restrictions. The overall symmetry of all three species is *C*_1_. This is due to the symmetry breaking caused by the rotational degree of freedom of the MeO groups. Averaging over all conceivable conformations and weighting the occupation probability according to the Boltzmann distribution was considered to be beyond the scope of this work. However, in a first approximation, the porphin core of the neutral porphyrin **9** exhibits approximate *S*_4_ symmetry and the radical cation **9**^•+^ and the dication **9**^**++**^ have *C*_2_ symmetry. According to the porphyrin literature, several “types” of distortions of the porphin ring are distinguished. Most important concerning the porphyrins in this work are ruffling and saddling. The two distortion modes can be visualized by the twisting or out-of-plane bending of two opposing pyrrole rings (see Table 6). To quantify the “degree of deformation”, several methods have been proposed. The NSD method^53,54^ is probably the most elegant, however, it is not very intuitive without in-depth knowledge of group theory (for NSD calculations, see Tables S3–S8). In Table 6, we use the dihedral angles Ψ and X to quantify the two distortion modes. Our calculations reveal that the out-of-plane distortion generally increases with increasing positive charge. The ruffling mode increases weakly and the saddle distortion increases strongly.

**Table 6:**
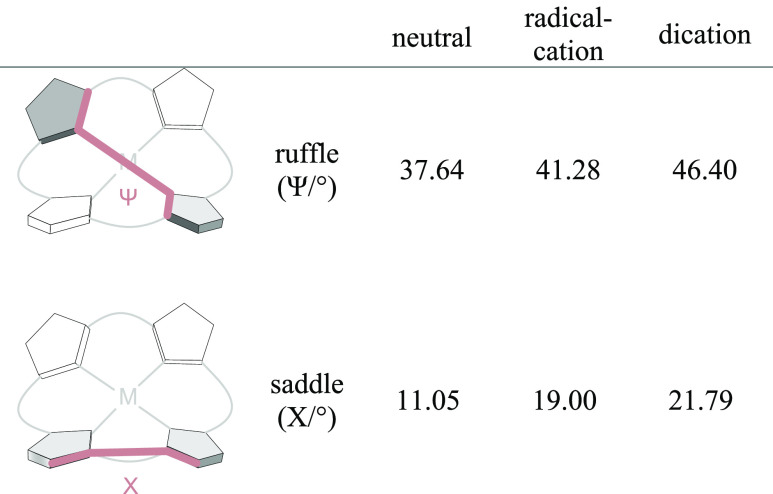
Calculated (PBE/def2-SVP) Dihedral Angles (Ψ and X [°]) Representing the Ruffling and Saddling Out-of-Plane Distortion for the Porphyrin Core of Neutral Porphyrin **9**, Radical Cation **9**^•+^, and Dication **9**^+2^

**Figure 10:**
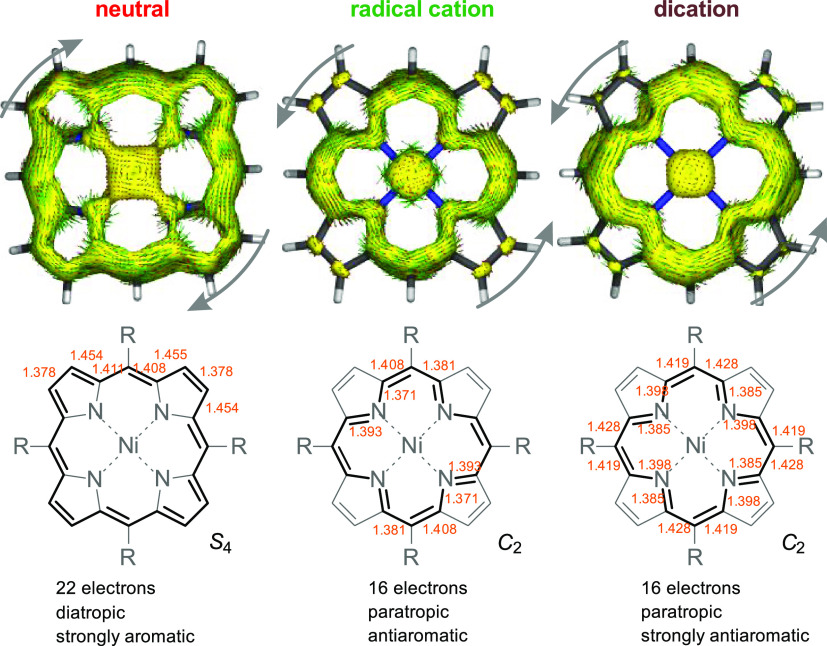
Orbital-separated ACID plots (only MOs with π character are included) for the neutral Ni porphin, the radical cation and dication. For reasons of clarity, the *meso*-substituents are hydrogen (R=H) (for full ACID plots of **9**, **9**^•+^, and **9**^**++**^ see SI “Computational Details”). Green arrows with red arrow heads indicate the direction and the strength of the ring current (current density vectors). The external magnetic field B points toward the viewer. The neutral porphin exhibits a strong diatropic ring current (clockwise). The radical cation and the dications show paratropic ring currents (counterclockwise). Note that the ring currents follow different cyclic pathways. The neutral Ni porphin is best described as an aromatic [20]annulene dianion (22 electrons), and the radical cation and dication are rather [16]annulenes which is also in line with the literature.^58^ The dication is strongly antiaromatic. The bond lengths [Å] (orange numbers) in the structures below are selected from the fully optimized structures (PBE/def2-SVP) of **9**, **9**^•+^, and **9**^**++**^ (R = 3,4,5-trimethoxyphenyl). The main cyclic π conjugation path is highlighted with bold lines.

Diatropic and paratropic ring currents within the porphyrin π system also have a strong influence on the NMR spectra. To investigate aromaticity and/or antiaromaticity of the neutral porphyrin **9**, the radical cation **9**^•+^, and the dication **9**^**++**^ we performed orbital-separated ACID calculations (Figure 10).^54,55^ Free base porphyrins are generally considered to exhibit [18]annulene type π systems. According to our ACID calculations (Figure 10), the Ni porphin is better described as a [20]annulene dianion π system with a strong diatropic (aromatic) ring current in the periphery of the macrocycle. The corresponding radical cation has a [16]annulene structure with a paratropic (antiaromatic) ring current that does not include any of the peripheral pyrrole double bonds. The paratropic ring current and thus the antiaromatic character are even more pronounced in the dication (Figures 10 and S50). The strong antiaromatic character of the dication **9**^**++**^ is also confirmed by the experimental and calculated ^1^H NMR spectra (see also Tables 4 and 5). Due to the strong diatropic ring current in the periphery of the neutral porphyrin macrocycle **9**, the pyrrole protons (analogous to the protons in benzene) are strongly deshielded and therefore low-field-shifted (8.86 ppm). Based on the reduced electron density in cations, a further downfield shift of the peripheral protons would be expected in the corresponding dication **9**^**++**^.^56,57^ The opposite is the case. The pyrrole protons in dication **9**^**++**^ are shifted by more than 2 ppm high field compared to the neutral porphyrin **9** (6.75 ppm, averaged). The very strong paratropic ring current in dication **9**^**++**^ leads to a shielding of the protons in the periphery. Obviously, this effect is so strong that it more than compensates for the deshielding by the positive charge.

The transition from the neutral system (aromatic) to the radical cation (weakly antiaromatic) and the dication (strongly antiaromatic) is also reflected by an increasing bond length alternation. The difference of the two C–N bond lengths in each pyrrole unit increases from 0.013 (neutral) and 0.022 (radical cation) to 0.055 Å (dication) (Figure 10). The increasing distortion with increasing positive charge is due to an increasing antiaromatic character. The system tries to avoid antiaromatic destabilization by reducing conjugation via bond length alternation and out-of-plane distortion. Although **9**^**++**^ is a strongly antiaromatic dication, it is remarkably stable under ambient conditions.

### Development of a Test Strip

To simplify the detection of homemade explosives (HMEs), such as TATP **1**, inorganic nitrates, and chlorate-based explosives, we have developed a test strip that contains [5,10,15,20-tetrakis(3,4,5-trimethoxyphenyl)porphyrinato]nickel(II) (**9**) as an indicator dye. The stick has a shelf life of two years (for details, see patent PCT/EP2021/058279).^59^

**Figure 11:**
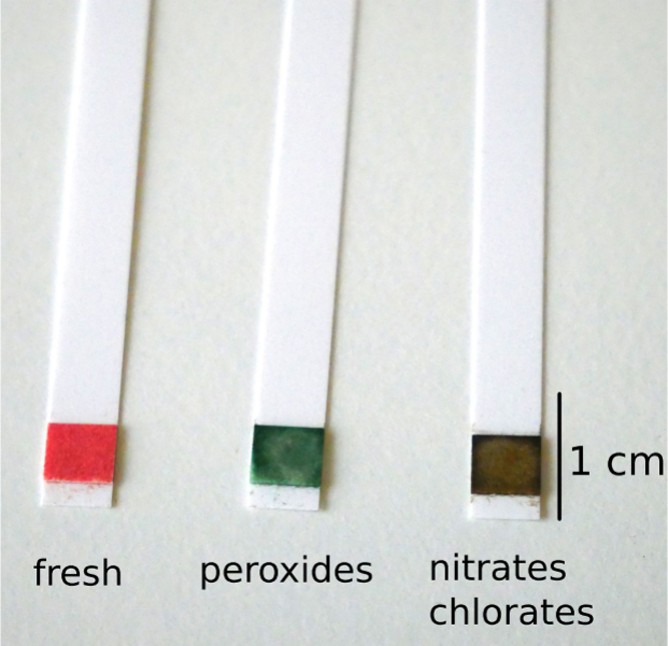
Test strips before and after detection of homemade explosives (HMEs). The pads of the sticks contain [5,10,15,20-tetrakis(3,4,5-trimethoxyphenyl)porphyrinato]nickel(II) (**9**) and are moistened (activated) with perfluoropentanoic acid. Left: fresh, no contact with explosives, red color; middle: peroxides; green color, right: nitrates or chlorates, deep brown.

For the detection of explosives, the stick is moistened (activated) with perfluoropentanoic acid and remains active for approximately 15 min. The stick is then brought into contact with the substance to be tested, resulting in a color change to green (peroxide-based explosives) or brown (inorganic nitrates or chlorate-based explosives) within 15 s (Figure 11).

**Figure 12:**
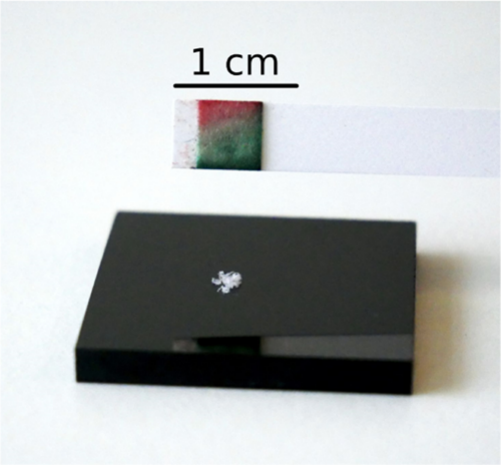
Test strip for the detection of homemade explosives (HMEs) was activated with perfluoropentanoic acid and held 5 mm above crystals of TATP **1**. A gradual color change from red to green was observed.

Among homemade explosives, TATP **1** (triacetone triperoxide) plays a particularly inglorious role. Since TATP **1** is easy to prepare but difficult to detect with standard methods, it is frequently used in terrorist attacks (e.g., Paris 2015, 130 dead, 416 injured, Brussels 2016, 32 dead, >300 injured). TATP **1** is also produced as a byproduct in the preparation of drugs (e.g., methamphetamine or MDMA), resulting in explosions.^60^ TATP **1** (nickname: Mother of Satan) is extremely sensitive to impact, shock, friction, and heat and can also explode spontaneously. During an operation, the first responders, therefore, must immediately determine their own security situation.^61^ Taking samples onsite, e.g., in a clandestine laboratory or at a postblast site and transportation to a well-equipped laboratory therefore is usually avoided. Destruction onsite is preferred, even if it involves collateral damage.^62^ The relatively high vapor pressure of TATP **1** (6.95 Pa at 25 °C)^16,17^ combined with the high sensitivity of our test now allows safe and simple noncontact detection of TATP **1** via the gas phase (Figure 12).

For demonstration, an activated stick is held approximately 5 mm above the substance to be tested. Alternatively, one can hold the stick into the headspace of a bottle containing a suspicious substance. In the case of TATP **1**, a gradual color change from red to green occurs within 15 s. This represents a significant gain in safety, as it is no longer necessary to come into contact with the explosive substance.

### Determination of the Detection Limits

The determination of detection limits on a test strip is less straightforward than in solution, where photometry can be applied. To determine the detection limits semiquantitatively (Table 7), we used the following procedure. First stock solutions of various homemade explosives (HMEs) in perfluoropentanoic acid were prepared.

**Table 7:** Detection Limits Determined with an Activated Stick

name	acronym	limit of detection [ng]	concentration
**triacetone triperoxide 1**	TATP	40	180 μM
hexamethylene triperoxide diamine	HMTD	50	240 μM
potassium chlorate	KClO_3_	270	2.2 mM
ammonium nitrate	AN	85	1.1 mM
urea nitrate	UN	350	2.8 mM

Ten μL of the stock solution was added to the stick in each case and a color change was allowed to occur within 1 min. This was repeated with decreasing concentrations. The test was considered to be positive if a slight color change was detected with the bare eye. Usually a 5- to 10-fold concentration is required for a complete color change of the test field. The detection limits determined in this way ranged in the two- to low three-digit nanogram range. It should also be noted that mixtures containing nitrate or chlorate, such as black powder or KClO_3_/sulfur, are detected.

## Conclusions

**Figure 13:**
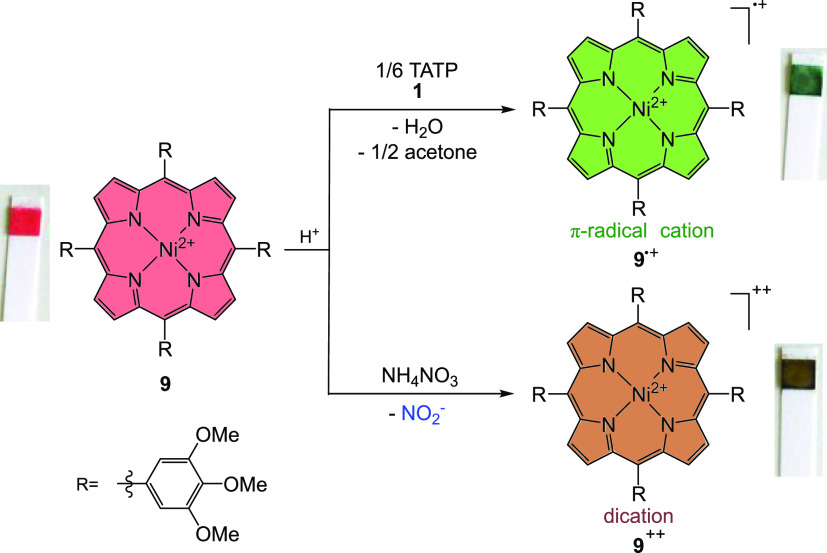
Overview of the detection of peroxide- and nitrate-based explosives. In the presence of a large excess of acid (TFA or perfluoropentanoic acid), a TATP **1** molecule is capable of oxidizing six porphyrin molecules **9** to the green π-radical cation **9**^•+^. In the case of nitrates and chlorates, the porphyrin **9** is converted to the brown dication **9**^**++**^ by two-electron oxidation.

We developed a very sensitive one-step colorimetric method for the trace detection of homemade explosives (HMEs) based on an electron-rich Ni porphyrin (**9**) as the indicator dye. Peroxide-based explosives, such as TATP **1** (triacetone triperoxide) or hexamethylene triperoxide diamine (**4**) (HMTD), give rise to a color change from red to green and inorganic nitrates or chlorates trigger a color change to deep brown. Spectroelectrochemical measurements, NMR, and UV–vis studies reveal that the green color is due to the formation of a porphyrin radical cation and the brown color can be attributed to the formation of a stable porphyrin dication (Figure 13). Both reaction mechanisms were elucidated.

Since both reactions are one-step, a simple (pH type) test strip could be developed for the detection of peroxides and nitrates/chlorates. TATP **1** and HMTD **4**, which are frequently used in terror attacks, are detected down to 40 or 50 ng, which is in the sensitivity range of sophisticated instruments, such as ion mobility spectrometers (IMSs) at security checks in airports. Detection limits for nitrates and chlorates are in the range between 85 and 350 ng. The test procedure is simple and robust. No power supply or electronics and no maintenance or warm-up period is needed and no professional training is necessary. The sticks and the activator acid are small enough to fit in any jacket or trouser pocket. Shelf life of the test sticks is two years. For the detection of the extremely sensitive TATP **1**, it is sufficient to bring the test strip close to the substance. We consider this a large gain in safety for first responders.

The porphyrin radical cation **9**^•**+**^ and particularly the dication **9**^**++**^ are antiaromatic but nevertheless remarkably stable under ambient conditions. They both exhibit a [16]annulene type π system. Unlike neutral free base and metal porphyrins, the peripheral pyrrole C=C double bonds are not included in the macrocyclic π system.

## Supporting Information Available

Cyclovoltametric measurements, synthesis of porpyhrin **9**, NMR spectra of acidic TATP **1** cleavage, full NMR spectra of TATP **1** titration with porphyrin **9**, UV spectra of demetalated porphyrin **9**, calculated structure of nickel porphyrins, full NMR spectra of NH_4_^15^NO_3_ titration with porphyrin **9**, NMR spectra of Pb(OAc)_4_ titration with porphyrin **9**, UV spectra of Pb(OAc)_4_ titration with porphyrin **9**; samples of sticks for explosive detection are available from the author RH (PDF)
