# Supplementary material for: Highly Sensitive, Easy-to-Use, One-Step Detection of Peroxide-, Nitrate- and Chlorate-Based Explosives with Electron-Rich Ni Porphyrins
Source: J Am Chem Soc. 2024 May 1;146(19):13010–24. doi: 10.1021/jacs.3c14118 (PMC11099979; doi:10.1021/jacs.3c14118)
Supplement: Supplementary file 1 — ja3c14118_si_001.pdf [file ja3c14118_si_001.pdf]

Supporting Information

Highly Sensitive, Easy-to-Use, One-Step Detection of Peroxide-, Nitrate- and Chlorate-Based Explosives with Electron-Rich Ni-Porphyrins

Mike Brockmann[1], Gabriel Glotz[2], Jan-Simon von Glasenapp[1], Lara Unterriker[1], Dmytro Neshchadin[2], Georg Gescheidt[2] and Rainer Herges*[1]

[1] Otto Diels-Institute of Organic Chemistry, Christian-Albrechts-Universität zu Kiel, Otto Hahn Platz 4, 24118 Kiel,
Germany; [2] Institute of Physical and Theoretical Chemistry, Graz University of Technology, Stremayrgasse 9, 8010 Graz, Austria

*E-mail: rherges@oc.uni-kiel.de

Contents

1. EXPERIMENTAL SECTION 2
1.1 Mass spectrometry 2
1.2 Melting point determination 2
1.3 Electron Spin Resonance Spectroscopy 2
1.4 UV/Vis spectroscopy 2
1.5 NMR spectroscopy 2
1.6 IR spectroscopy 3
1.7 Column chromatography 3
1.8 Chemicals and solvents 3
2. Synthesis 3
2.1 Synthesis of 5,10,15,20-Tetrakis(2,3,4,5,6-pentafluorphenyl)-21*H*,23*H*-porphyrin 3
2.2 Synthesis of 5,10,15,20-Tetraphenyl-21*H*,23*H*-porphyrin 4
2.3 Synthesis of 5,10,15,20-Tetrakis(2,4,6-trimethylphenyl)-21*H*,23*H*-porphyrin 4
2.4 Synthesis of 5,10,15,20-Tetrakis(3,4,5-trimethoxyphenyl)-21*H*,23*H*-porphyrin 5
2.5 General metallation procedure of porphyrins: 5
2.7 5,10,15,20-Tetraphenylnickel(II)porphyrin **6** 6
2.8 [5,10,15,20-Tetrakis(2,4,6-trimethylphenyl)porphyrinato]nickel(II) **7** 6
2.10 [5,10,15,20-Tetrakis(3,4,5-trimethoxyphenyl)porphyrinato]nickel(II) **9** 7
2.11 [5,10,15,20-Tetrakis(3,4,5-trimethoxyphenyl)porphyrinato]copper(II) **10** 7
2.12 [5,10,15,20-Tetrakis(3,4,5-trimethoxyphenyl)porphyrinato]palladium(II) **11** 8
3. Evaluation of different solvents for the detection of explosives 8
4. Detection of prostaglandin H_2, artemisinin, other peroxides and everyday hygiene products 8

5. Proposed mechanisms including the intermediacy of a nickel(IV) oxo species 10
6. UV-spectra.. 10
6.1 Determination of the rate constants ***k*** of porphyrins **5**, **6**, **7**, **8**, **9**, **10** and **11**.......................... 10
6.2 Mechanistic studies for the detection of peroxide-based explosives based on UV measurements 13
6.3 Preparation of stock solutions for UV titration of **9** with different oxidants.......................... 13
7. NMR spectra.. 16
7.1 Acid promoted decomposition of TATP **1** 16
7.2 Mechanistic studies for peroxide-based explosives based on NMR measurements................ 16
8. CV and Spectroelectrochemistry.. 22
8.1 Experimental 22
9. Computational Details.. 32
References.. 42

1. EXPERIMENTAL SECTION

1.1 Mass spectrometry

Electron ionization mass spectra were measured at 70 eV ionization energy using a JEOL Accu-TOF 4G mass spectrometer (EI). Electrospray ionization mass spectra (ESI-MS) were recorded using a FINNIGAN LCQ Deca quadrupole ion trap mass spectrometer.

1.2 Melting point determination

Melting points were determined with the Melting Point B-540 from BÜCHI and taken without correction.

1.3 Electron Spin Resonance Spectroscopy

Electron spin resonance (ESR) spectra were obtained using BRUKER's EMXplus spectrometer equipped with a PremiumX microwave bridge and a dual mode X-band cavity or a BRUKER's HQ X-band cavity.

1.4 UV/Vis spectroscopy

The UV/Vis spectra were measured with the UV-2600i UV/Vis spectrophotometer from SHIMADZU. Quartz cells with a diameter of 1 cm were used.

1.5 NMR spectroscopy

All NMR spectra were recorded in deuterated solvents from DEUTERO. Two-dimensional NMR techniques were used to assign the signals (COSY, HSQC, HMBC). The following table indicates both the degree of deuteration of each solvent and the solvent signal. Tetramethylsilane (TMS) was used as the internal standard for referencing the spectra.

solvent	deuteration level	^{1}H-Signal / ppm	^{13}C-Signal / ppm
CD_2Cl_2	99.8 %	5.32 (triplet)	53.84 (quintet)
$CDCl_3$	99.8 %	7.26 (singlet)	77.16 (triplet)

The following spectrometers were used:

	frequency / MHz			
device name	^{1}H-NMR spectra	^{13}C-NMR spectra	^{19}F-NMR spectra	^{15}N-NMR spectra
Bruker AC 200	200			
Bruker DRX 500	500	125	470	
Bruker AV 600	600	150		600

For the evaluation of the NMR spectra of the porphyrins, the carbon atoms were numbered according to the following example.

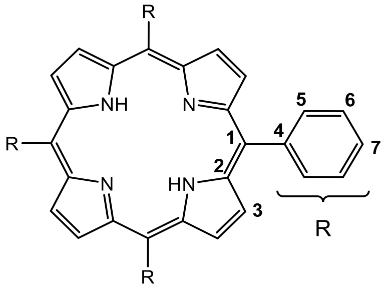

The atoms were numbered according to the scheme above. Starting from the *meso* position of the porphyrin, the porphyrin was numbered first. The substituent at the *meso* position of the porphyrin followed in the sequence indicated. The symmetry of the molecules was considered in the numbering.

Some carbon atoms of certain porphyrins could be assigned via the heteronuclear single quantum coherence (HSQC) and the heteronuclear multiple bond correlation (HMBC) spectra.

1.6 IR spectroscopy

Infrared spectra were recorded using a PERKIN-ELMER 1600 Series FT-IR spectrometer with A531-G Golden Gate Diamond ATR unit. Signals are described with the abbreviations w, m, and s for weak, medium, and strong intensity. Broad bands are additionally assigned with br.

1.7 Column chromatography

Column chromatographic separations were performed by flash chromatography on MERCK silica gel (grain size: 0.04-0.063 nm) and with the solvents indicated below.

1.8 Chemicals and solvents

The chemicals were purchased commercially and used without further preparation. Solvents used for synthesis were purified by distillation. For synthesis with air or moisture sensitive intermediates, work was carried out under a nitrogen atmosphere.

2. Synthesis

2.1 Synthesis of 5,10,15,20-Tetrakis(2,3,4,5,6-pentafluorphenyl)-21*H*,23*H*-porphyrin

Under nitrogen atmosphere, 2,3,4,5,6-pentafluorobenzaldehyde (3.82 g, 19.5 mmol), freshly distilled pyrrole (1.70 g, 25.3 mmol), and boron trifluoride diethyl etherate (300 µL, 2.40 mmol) were placed in 320 mL dry dichloromethane.

The reaction mixture was stirred at room temp. for 15 h, and then *p*-chloroanil (1.90 g, 7.70 mmol) was added and stirred at room temp. for an additional 3 h. The reaction was continued for 3 hours. The solvent was removed i. vac. and the crude product was purified by column chromatography on silica gel (*n*-pentane/dichloromethane, 3:1, $R_f = 0.50$). The product was obtained as a violet solid.

Yield: 1.89 g (1.93 mmol, 40%) (Lit.:[1] 41%).

^{1}H NMR (500 MHz, $CDCl_3$, 300 K, TMS): δ = 8.92 (s, 8 H, *H*-3), -2.91 (s, 2 H, N-*H*) ppm.

^{13}C NMR (125 MHz, $CDCl_3$, 300 K, TMS): δ = 143.0 (*C*-1), 132.6 (*C*-3), 103.1 (*C*-2) ppm.

Due to the large number of quaternary C atoms and the large number of ^{19}F-coupled C atoms, insufficient signal intensities were obtained in ^{13}C NMR spectroscopy. For this reason, the signals from the carbon atoms in the phenyl ring could not be evaluated.

^{19}F NMR (470 MHz, $CDCl_3$, 300 K): δ = -136.99 (dd, ^3J = 23.1 Hz, ^4J = 7.7 Hz, 8 F, *o-F*), -151.68 (t, ^3J = 20.8 Hz, 4 F, *p-F*), -161.78 (td, ^3J = 22.3 Hz, ^4J = 7.4 Hz, 8 F, *m-F*) ppm.

MS (EI, 70 eV): m/z (%) = 974 (100) [$M^{\cdot+}$].

MS (EI, HR, 70 eV): $C_{44}H_{10}F_{20}N_4$, m/z = calc.: 974.05861 u, found: 974.05911 u, diff.: -0.51 ppm.

MP: >400 °C.

FT-IR: $\tilde{\nu}$ = 2929 (s), 2154 (w), 1992 (w), 1495 (s), 1074 (m), 1042 (m), 979 (s), 913 (s), 805

(s), 767 (s), 755 (s), 724 (s), 632 (w), 542 (w), 501 (w) cm^{-1}.

2.2 Synthesis of 5,10,15,20-Tetraphenyl-21*H*,23*H*-porphyrin

Under nitrogen atmosphere, benzaldehyde (2.38 g, 22.4 mmol), freshly distilled pyrrole (1.50 g, 22.4 mmol), and boron trifluoride diethyl etherate (325 µL, 2.60 mmol) were dissolved in dry dichloromethane (340 mL) and stirred for 18 h at room temp. Subsequently, *p*-chloroanil (2.19 g, 8.93 mmol) was added and stirred for another 3 h at room temp. The solvent was removed i. vac. and the residue was purified by column chromatography on silica gel (n-pentane/dichloromethane, 3:2, $R_f = 0.45$). The product was obtained as a purple solid.

Yield: 574 mg (930 µmol, 16%).

^{1}H NMR (500 MHz, $CDCl_3$, 300 K, TMS): δ = 8.87 (s, 8 H, *H*-3), 8.24 (dd, ^3J = 7.6 Hz, ^4J = 1.4 Hz, 8 H, *H*-5), 7.82-7.74 (m, 12 H, *H*-6, 7), -2.74 (s, 2 H, N-*H*) ppm.

^{13}C NMR (125 MHz, $CDCl_3$, 300 K, TMS): δ = 142.1 (*C*-4), 134.6 (*C*-5), 127.7 (*C*-7), 126.7 (*C*-6), 120.2 (*C*-1) ppm.

MS (EI, 70 eV): m/z (%) = 614 (100) [$M^{\cdot+}$].

MS (EI, HR, 70 eV): $C_{44}H_{30}N_4$, m/z = calc.: 614.24705 u, found: 614.24718 u, diff.: -0.21 ppm.

MP: >400 °C.

FT-IR: $\tilde{\nu}$ = 3329 (w), 3058 (w), 1542 (w), 1521 (w), 1468 (w), 1423 (w), 1352 (w), 967 (w), 801 (s), 687 (m), 665 (s) cm^{-1}.

2.3 Synthesis of 5,10,15,20-Tetrakis(2,4,6-trimethylphenyl)-21*H*,23*H*-porphyrin

Under nitrogen atmosphere, 2,4,6-trimethylbenzaldehyde (3.32 g, 22.4 mmol), freshly distilled pyrrole (1.50 g, 22.4 mmol), and boron trifluoride diethyl etherate (325 µL, 2.60 mmol) were dissolved in dry dichloromethane (360 mL) and stirred for 19 h at room temp. Subsequently, *p*-Chloranil (2.19 g, 8.93 mmol) was added and stirred for another 3 h at

room temp. The solvent was removed i. vac. and the residual solid was purified by column chromatography on silica gel (n-pentane/dichloromethane, 3:2, R_f = 0.60). The product was obtained as a purple solid.

Yield: 481 mg (610 µmol, 11 %).

^{1}H-NMR (500 MHz, $CDCl_3$, 300 K, TMS): δ = 8.62 (s, 8 H, *H*-3), 7.27 (s, 8 H, *H*-6), 2.62 (s, 12 H, *H*-9), 1.86 (s, 24 H, *H*-8), -2.50 (s, 2 H, N-*H*) ppm.

^{13}C-NMR (125 MHz, $CDCl_3$, 300 K, TMS): δ = 139.5 (*C*-5), 138.3 (*C*-4), 137.6 (*C*-7), 127.7 (*C*-6), 117.6 (*C*-2), 21.7 (*C*-8), 21.5 (*C*-9) ppm.

MS (EI, 70 eV): m/z (%) = 782 (100) [$M^{\cdot+}$].

MS (EI, HR, 70 eV): $C_{56}H_{54}N_4$, m/z = calc.: 782.43485 u, found: 782.43442 u, diff.: 0.55 ppm.

MP: >400 °C.

FT-IR: $\tilde{\nu}$ = 3318 (w), 2957 (w), 2920 (w), 2825 (w), 1608 (w), 1563 (w), 1469 (s), 1434 (m), 1375 (w), 1340 (w),1146 (m), 1064 (w), 1042 (w), 987 (s), 967 (s), 942 (w), 849 (m), 822 (m), 800 (w), 788 (w), 734 (s), 639 (w), 523 (w) cm^{-1}.

2.4 Synthesis of 5,10,15,20-Tetrakis(3,4,5-trimethoxyphenyl)-21*H*,23*H*-porphyrin

Under nitrogen atmosphere, 3,4,5-trimethoxybenzaldehyde (1.92 g, 9.80 mmol) was dissolved in 200 mL of dry dichloromethane and TFA (226 µL, 2.94 mmol) was added. The reaction mixture was heated to reflux and stirred for 30 min. Subsequently, freshly distilled pyrrole (658 mg, 9.80 mmol) was added and stirred for another 2.5 h at reflux. Subsequently, 4,5-dichloro-3,6-dioxocyclohexa-1,4-diene-1,2-dicarbonitrile (DDQ) (889 mg, 3.92 mmol) was added and stirred for another 3.5 h under reflux. After cooling to room temp., the solvent was removed i. vac. The residue was purified by column chromatography on silica gel (cyclohexane/ethyl acetate, 1:1, *Rf* = 0.54). Purple crystals were obtained.

Yield: 262 mg (269 µmol, 11%).

^{1}H NMR (500 MHz, $CDCl_3$, 300 K, TMS): δ = 8.96 (s, 8 H, *H*-3), 7.47 (s, 8 H, *H*-5), 4.19 (s, 12 H, *H*-9), 3.97 (s, 24 H, *H*-8), -2.78 (s, 2 H, N-*H*) ppm.

^{13}C NMR (125 MHz, $CDCl_3$, 300 K, TMS): δ = 152.0 (*C*-7), 137.9 (*C*-6), 137.6 (*C*-4), 120.1 (*C*-1), 112.8 (*C*-5), 61.5 (*C*-9), 56.4 (*C*-8) ppm.

MS (EI, 70 eV): m/z (%) = 975 (100) [$M^{\cdot+}$].

MS (EI, HR, 70 eV): $C_{56}H_{54}O_{12}N_4$, m/z = calc.: 974.38110 u, found: 974.37901 u, diff.: 2.15 ppm.

MP: 321 °C.

FT-IR: $\tilde{\nu}$ = 2931 (w), 2897 (w), 2821 (w), 1593 (m), 1507 (m), 1483 (m), 1407 (m), 1397 (m), 1239 (m), 1109 (s), 997 (m), 983 (m), 897 (m), 803 (w), 771 (m), 709 (m), 665 (w), 518 (w) cm^{-1}.

2.5 General metallation procedure of porphyrins:

270 µmol of the porphyrin was dissolved in 50 mL of toluene and 2.70 mmol of nickel(II) acetylacetonate was added. The reaction mixture was stirred for 3 d (electron poor porphyrins **5, 6**) / 1 d (electron rich porphyrins **7, 8, 9**) under reflux. After cooling to room temp. the solvent was removed i. vac. and the residue was purified by column chromatography on silica gel (cyclohexane/ethyl acetate, 1:1). The product was obtained in each case as a red solid.

2.6 [5,10,15,20-Tetrakis(2,3,4,5,6-pentafluorphenyl)porphyrinato]nickel(II) **5**

Yield: 89 %.

^{1}H NMR (500 MHz, $CDCl_3$, 300 K, TMS): δ = 8.79 (s, 8 H, *H*-3) ppm.

^{13}C-NMR (125 MHz, $CDCl_3$, 300 K, TMS): δ = 143.1 (*C*-1), 132.8 (*C*-3), 103.0 (*C*-2) ppm.

Due to the large number of quaternary C atoms and the large number of ^{19}F-coupled C atoms, insufficient signal intensities were obtained in ^{13}C NMR spectroscopy. For this reason, the signals from the carbon atoms in the phenyl ring could not be evaluated.

^{19}F NMR (470 MHz, $CDCl_3$, 300 K): δ = -137.11 (dd, ^{3}J = 23.0 Hz, ^{4}J = 7.7 Hz, 8 F, *o-F*), -151.75 (t, ^{3}J = 20.8 Hz, 4 F, *p-F*), -161.11 (td, ^{3}J = 23.5 Hz, ^{4}J = 8.4 Hz, 8 F, *m-F*) ppm.

MS (EI, 70 eV): m/z (%) = 1030 (90) [$M^{\cdot+}$].

MS (EI, HR, 70 eV): $C_{44}H_8F_{20}N_4Ni$, m/z = calc.: 1029.97831 u, found: 1029.97738 u, diff.: 0.89 ppm.

MP: >400 °C.

FT-IR: ν˜ = 2161 (m), 1490 (s), 1345 (w), 1061 (m), 987 (s), 935 (s), 842 (w), 759 (s), 691 (m) cm^{-1}.

2.7 5,10,15,20-Tetraphenylnickel(II)porphyrin **6**

Yield: 94 %.

^{1}H-NMR (500 MHz, $CDCl_3$, 300 K, TMS): δ = 8.74 (s, 8 H, *H*-3), 8.01 (dd, ^{3}J = 7.5 Hz, ^{4}J = 1.3 Hz, 8 H, *H*-5), 7.72-7.65 (m, 12 H, *H*-6, 7) ppm.

^{13}C-NMR (125 MHz, $CDCl_3$, 300 K, TMS): δ = 142.6 (*C*-1), 140.9 (*C*-4), 133.7 (*C*-5), 132.2 (*C*-3), 127.7 (*C*-7), 126.8 (*C*-6) ppm.

MS (EI, 70 eV): m/z (%) = 670 (100) [$M^{\cdot+}$].

MS (EI, HR, 70 eV): $C_{44}H_{28}N_4Ni$, m/z = calc.: 670.16674 u, found: 670.16664 u, diff.: 0.15 ppm.

MP: >400 °C.

FT-IR: ν˜ = 1598 (w), 1437 (w), 1347 (w), 1255 (m), 1071 (m), 1002 (s), 791 (s), 735 (m), 707 (m), 693 (m) cm^{-1}.

2.8 [5,10,15,20-Tetrakis(2,4,6-trimethylphenyl)porphyrinato]nickel(II) **7**

Yield: 81 %.

^{1}H-NMR (500 MHz, $CDCl_3$, 300 K, TMS): δ = 8,53 (s, 8 H, *H*-3), 7,20 (s, 8 H, *H*-6), 2,57 (s, 12 H, *H*-9), 1,82 (s, 24 H, *H*-8) ppm.

^{13}C-NMR (125 MHz, $CDCl_3$, 300 K, TMS): δ = 143,0 (*C*-1), 139,4 (*C*-5), 137,6 (*C*-4), 137,5 (*C*-7), 131,0 (*C*-3), 127,6 (*C*-6), 21,4 (*C*-8), 21,4 (*C*-9) ppm.

MS (ESI): m/z (%) = 838 (100) [M^+].

MS (ESI, HR): $C_{56}H_{54}N_4$, m/z = calc.: 838.35400 u, found: 838.35477 u, diff.: -0.92 ppm.

MP: 387 °C.

FT-IR: ν˜ = 2950 (w), 2901 (w), 2881 (w), 1703 (m), 1582 (w), 1473 (s), 1436 (w), 1367 (s), 1251 (w), 1236 (w),1103 (m), 1001 (s), 883 (w), 797 (s), 783 (s), 681 (s), 581 (m), 521 (w), 487 (w) cm^{-1}.

2.9 [5,10,15,20-Tetrakis(4-Methoxyphenyl)porphyrinato]nickel(II) **8**

Yield: 71 %.

^{1}H-NMR (500 MHz, CDCl$_3$, 300 K, TMS): δ = 8.76 (s, 8 H, *H*-3), 7.92 (d, ^{3}J = 8.4 Hz, 8 H, *H*-6), 7.21 (d, ^{3}J = 8.4 Hz, 8 H, *H*-5), 4.05 (s, 12 H, *H*-8) ppm.

^{13}C-NMR (125 MHz, CDCl$_3$, 300 K, TMS): δ = 159.5 (*C*-7), 143.1 (*C*-2), 134.7 (*C*-6), 133.2 (*C*-1), 132.0 (*C*-3), 118.5 (*C*-4), 112.4 (*C*-5), 55.5 (*C*-8) ppm.

MS (EI, 70 eV): m/z (%) = 790 (80) [$M^{\cdot+}$].

MS (EI, HR, 70 eV): $C_{44}H_{28}N_4Ni$, m/z = calc.: 790.20845 u, found: 790.20901 u, diff.: -0.71 ppm.

MP: >400 °C.

FT-IR: ν˜ = 2907 (w), 2821 (w), 1603 (m), 1574 (w), 1532 (w), 1501 (s), 1455 (m), 1441 (m), 1382 (s), 1291 (m), 1252 (s), 1207 (s), 1137 (m), 1019 (m), 997 (s), 827 (w), 801 (s), 773 (s), 691 (m), 603 (w), 591 (m), 537 (m), 451 (w) cm−1.

2.10 [5,10,15,20-Tetrakis(3,4,5-trimethoxyphenyl)porphyrinato]nickel(II) **9**

Yield: 79 %.

^{1}H-NMR (500 MHz, CDCl$_3$, 300 K, TMS): δ = 8.86 (s, 8 H, *H*-3), 7.28 (s, 8 H, *H*-5), 4.13 (s, 12 H, *H*-9), 3.93 (s, 24 H, *H*-8) ppm.

^{13}C-NMR (125 MHz, CDCl$_3$, 300 K, TMS): δ = 151.6 (*C*-6), 142.8 (*C*-2), 137.9 (*C*-7), 136.3 (*C*-4), 132.2 (*C*-3), 119.0 (*C*-1), 111.9 (*C*-5), 61.3 (*C*-9), 56.3 (*C*-8) ppm.

MS (EI, 70 eV): m/z (%) = 1030 (30) [$M^{\cdot+}$].

MS (EI, HR, 70 eV): $C_{56}H_{52}N_4O_{12}Ni$, m/z = calc.: 1030.29352 u, found: 1030.29353 u, diff.: -0.001 ppm.

MP: 277 °C.

FT-IR: ν˜ = 3021 (w), 2971 (w), 2907 (w), 2809 (w), 1607 (m), 1511 (m), 1491 (m), 1483 (m), 1423 (s), 1379 (s), 1333 (m), 1241 (s), 1208 (w), 1132 (s), 1072 (m), 1018 (s), 971 (m), 837 (w), 778 (m), 769 (m), 750 (m), 723 (s), 599 (w), 521 (w), 471 (w) cm^{-1}.

2.11 [5,10,15,20-Tetrakis(3,4,5-trimethoxyphenyl)porphyrinato]copper(II) **10**

Yield: 59 %.

^{1}H-NMR (500 MHz, CDCl$_3$, 300 K, TMS): δ = 4.07 (s, 12 H, *H*-9), 3.88 (s, 24 H, *H*-8) ppm.

^{13}C-NMR (125 MHz, CDCl$_3$, 300 K, TMS): δ = 61.1 (*C*-7), 56.3 (*C*-6) ppm.

Due to paramagnetism, the pyrrole and o protons cannot be determined. The same applies to the other carbon atoms.

MS (ESI): m/z (%) = (1035) (100) [M^{+}].

MS (ESI, HR): $C_{56}H_{52}N_4O_{12}Cu$, m/z = calc.: 1035.28723 u, found: 1035.28747 u, diff.: -0.23 ppm.

MP: > 290°C (decomposition).

FT-IR: ṽ = **FT-IR:** ṽ = 2932 (br, w), 2833 (br, w), 2323 (w), 2251 (w), 2212 (w), 2161 (w), 2139 (w), 2110 (w), 2048 (w), 2024 (w), 1986 (w), 1732 (br, w), 1577 (m), 1501 (m), 1448 (m), 1408 (m), 1354 (m), 1321 (w), 1275 (w), 1233 (m), 1163 (w), 1117 (m), 1074 (m), 1001 (m), 941 (m), 895 (w), 861 (w), 800 (w), 784 (w), 760 (w), 722 (m), 685 (w), 646 (w), 612 (w), 521 (w), 506 (w), 494 (w), 454 (m), 442 (w), 427 (w), 411 (w) cm^{-1}.

2.12 [5,10,15,20-Tetrakis(3,4,5-trimethoxyphenyl)porphyrinato]palladium(II) **11**

Yield: 93 %.

^{1}H-NMR (500 MHz, $CDCl_3$, 300 K, TMS): δ = 8.93 (s, 8 H, *H*-3), 7.42 (s, 8 H, *H*-5), 4.18 (s, 12 H, *H*-9), 3.96 (s, 24 H, *H*-8) ppm.

^{13}C-NMR (125 MHz, $CDCl_3$, 300 K, TMS): δ = 151.5 (*C*-6), 141.7 (*C*-2), 137.9 (*C*-7), 137.1 (*C*-4), 131.0 (*C*-3), 121.7 (*C*-1), 112.3 (*C*-5), 61.3 (*C*-9), 56.4 (*C*-8) ppm.

MS (EI, 70 eV): m/z (%) = (1078) (100) [$M^{\cdot +}$].

MS (EI, HR, 70 eV): $C_{56}H_{52}N_4O_{12}Pd$, m/z = calc.: 1078.26165 u, found: 1078.26229 u, diff.: -0.59 ppm.

MP: 340.7°C.

FT-IR: ṽ = 3647 (w), 2930 (br, w), 2832 (br, w), 2285 (w), 2228 (w), 2187 (w), 2160 (w), 2109 (w), 2046 (w), 2008 (w), 1579 (m), 1503 (m), 1447 (m), 1047 (m), 1357 (m), 1233 (m), 1182 (w), 1164 (w), 1121 (m), 1075 (m), 1005 (m), 949 (m), 927 (w), 859 (w), 814 (m), 798 (m), 761 (w), 718 (m), 642 (w), 523 (w), 480 (w), 465 (w), 442 (w), 425 (w), 414 (m) cm^{-1}.

3. Evaluation of different solvents for the detection of explosives

To identify suitable solvents for the detection of explosives, stock solutions (25 µM) were prepared in each solvent. Of this stock solution, 300 µl was used in each case and 70 µl of TFA was added. Then 10 µl of a 970 µM solution of TATP 1 (in DCM) was added and a visual color change was monitored.

Table S1. Detection of TATP 1 in different solvents and miscibility with TFA.

solvent	color change	miscibility
Sulfolane	-	+
Dimethyl sulfoxide	-	+
Dimethylpropylene urea	-	+
Toluene	-	+
Bromoform	-	+
Diethyl ether	-	-
Cyclohexane	-	-
n-Pentane	-	-
Water	-	-
Ethylacetate	-	+
Acetone	-	+

4. Detection of prostaglandin H_2, artemisinin, other peroxides and everyday hygiene products

For the detection of prostaglandin H_2 and artemisinin, a test strip provided with porphyrin **9** was moistened with perfluoropentanoic acid (~10 µl). Subsequently, 0.2 mg prostaglandin H_2 was added to the activated stick. An immediate color change from red to green occurred. In the case of artemisinin, 0.2 mg was also added to an activated stick. Only after about 10 minutes a slight discoloration from red to green occurred.

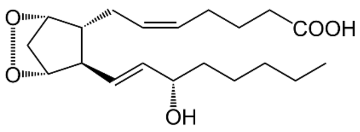

Prostaglandin H_2

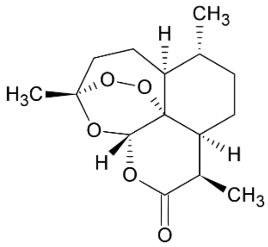

Artemisinin

For the detection of various peroxides and everyday hygiene products, a test strip containing porphyrin 9 was moistened with perfluoropentanoic acid (10 µl). The substance to be tested was then added to the test strip. A green coloration within 10 minutes was considered a positive test result.

Table S2. Detection of different peroxides and everyday hygiene products using the Ni-porphyrin 9 based test strip which is activated with perfluoropentanoic acid.

Substance	Result
**H_2O_2**	Positive
**Benzoylperoxide**	Positive
***tert*-Butyl hydroperoxide**	Positive
***meta*-Chloroperoxybenzoic acid**	Positive
**Mold remover (NaOCl 43 g/l)**	Positive
**Mold remover (H_2O_2 (7.9 %))**	Positive
**Detergents (solid and liquid)**	Negative
**Toothpaste**	Negative
**Deodorant**	Negative
**Parfume**	Negative

5. Proposed mechanisms including the intermediacy of a nickel(IV) oxo species

Scheme S1. Proposed mechanism of the formation of the green radical cation $9^{\cdot+}$ including a possible equilibrium with the TFA radical. [2–4]

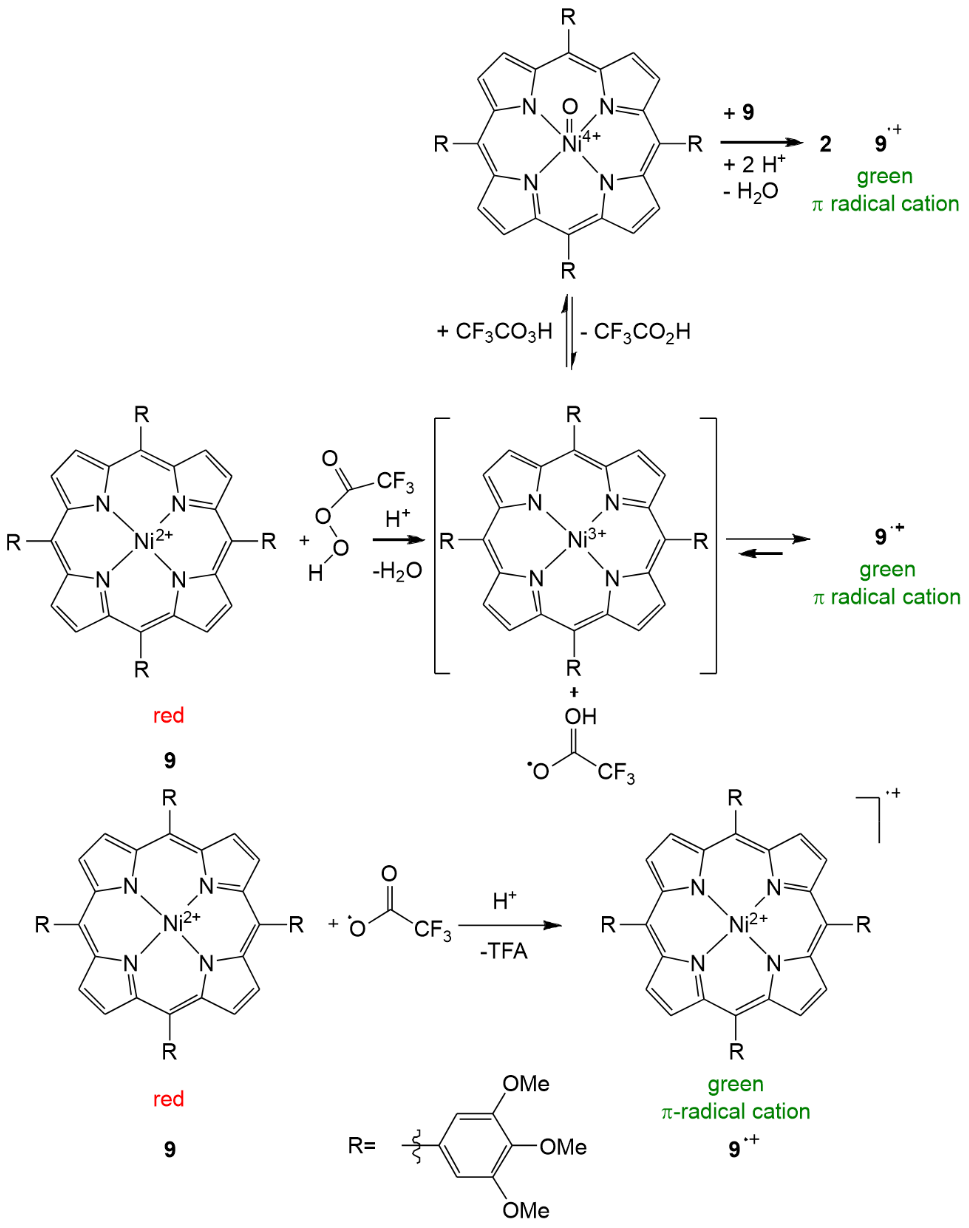

6. UV-spectra

6.1 Determination of the rate constants *k* of porphyrins **5**, **6**, **7**, **8**, **9**, **10** and **11**

UV/Vis measurements were performed to determine the response time of porphyrins **5**, **6**, **7**, **8**, **9**, **10** and **11**. For this purpose, 2 ml of porphyrin stock solution (5 µM in dry and degassed DCM) and 10 equivalents TATP **1** (with respect to the porphyrin; 333 µM solution in dry DCM) were placed in a cuvette. Afterwards a 49 700-fold excess of TFA was

added and a UV spectrum was measured immediately. A spectrum was then recorded every 18 s over a period of 7.5 min.

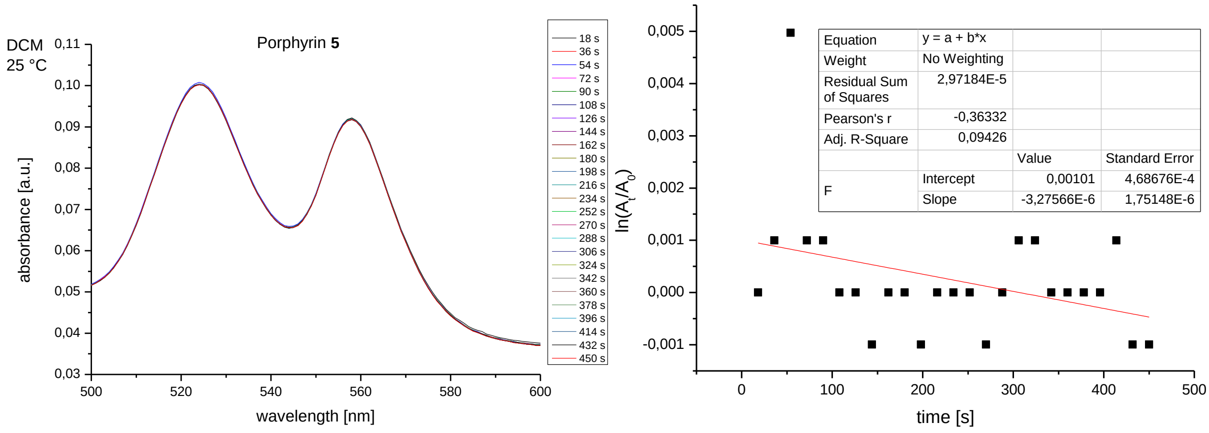

Figure S1. UV-spectra of porphyrin 5 after addition of 10 eq. TATP **1** and a 49700-fold excess of TFA. Even after 450 s no π radical cation **5**$^{\cdot+}$ was formed and the spectra of porphyrin **5** remains unchanged. The measurements were carried out in dry and degassed dichloromethane at 25 °C.

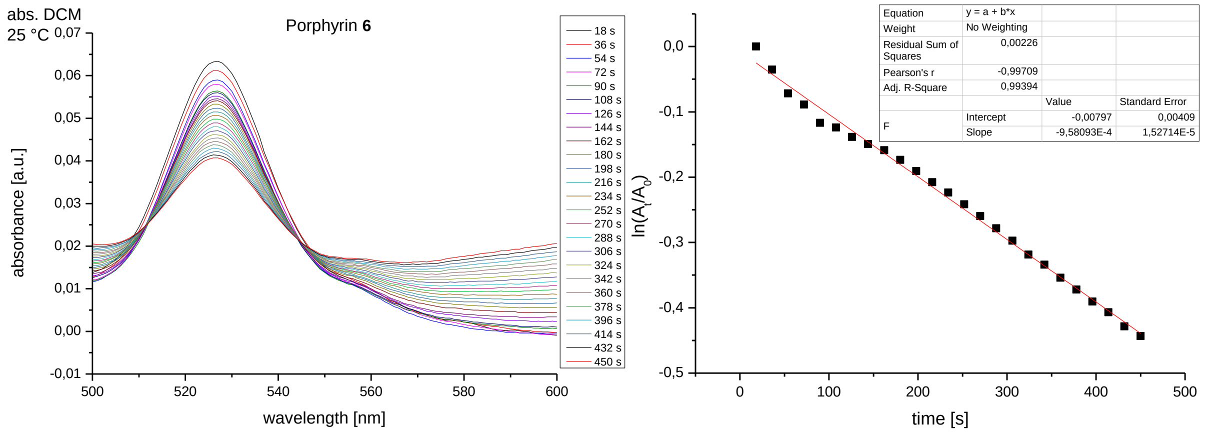

Figure S2. UV-spectra of porphyrin **6** after addition of 10 eq. TATP **1** and a 49700-fold excess of TFA. Plotting ln(A_t/A_0) against time gives a rate constant of k=9.6·10^{-4} s^{-1}. The measurements were carried out in dry and degassed dichloromethane at 25 °C.

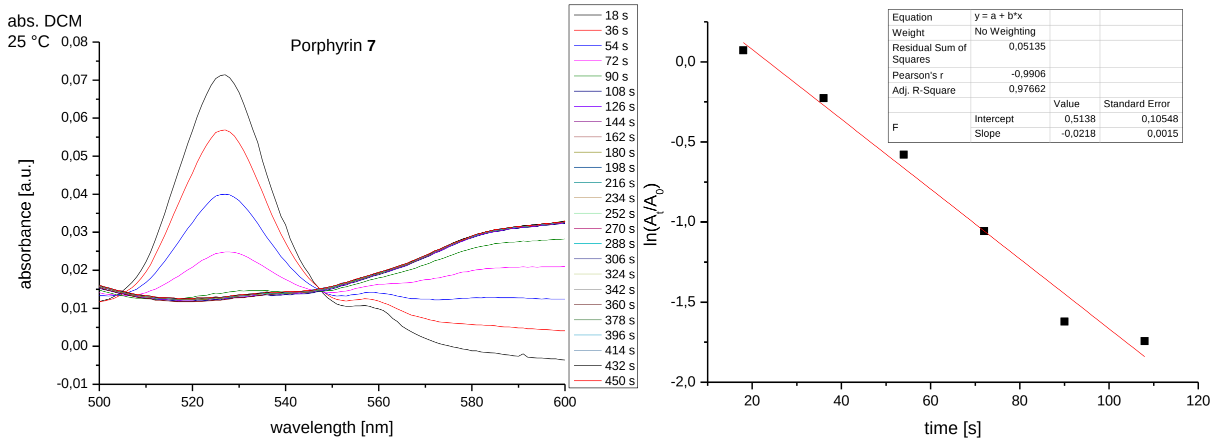

Figure S3. UV-spectra of porphyrin **7** after addition of 10 eq. TATP **1** and a 49700-fold excess of TFA. Plotting $\ln(A_t/A_0)$ against time gives a rate constant of $k=2.2\cdot10^{-2}$ s^{-1}. The measurements were carried out in dry and degassed dichloromethane at 25 °C.

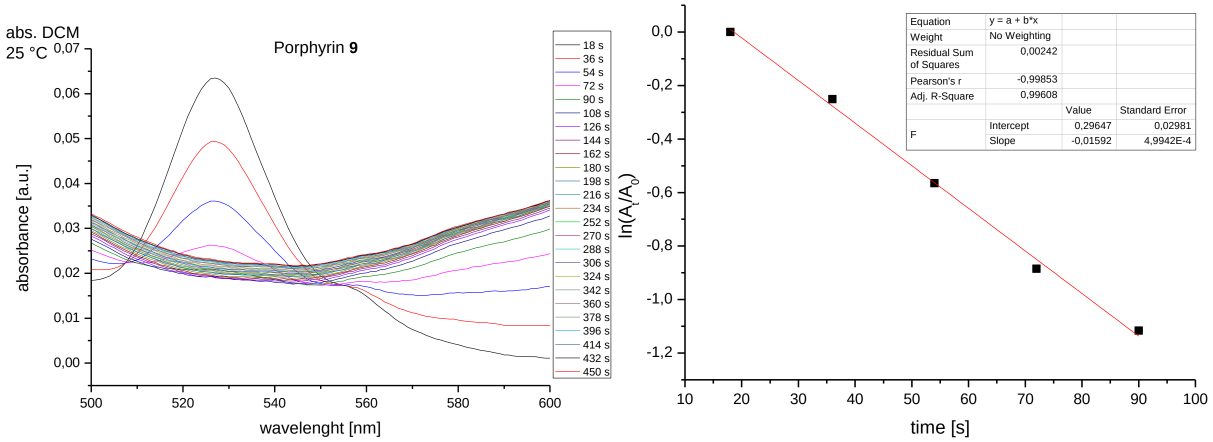

Figure S4. UV-spectra of porphyrin **9** after addition of 10 eq. TATP **1** and a 49700-fold excess of TFA. Plotting $\ln(A_t/A_0)$ against time gives a rate constant of $k=1.6\cdot10^{-2}$ s^{-1}. The measurements were carried out in dry and degassed dichloromethane at 25 °C. After complete conversion to the radical cation (90 s) the dication forms and the isosbestic point is lost.

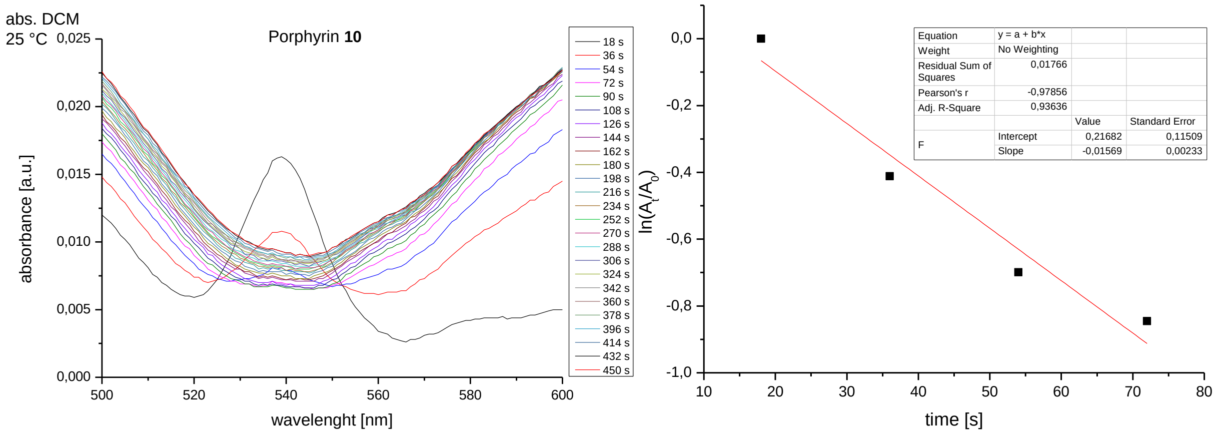

Figure S5. UV-spectra of porphyrin **10** after addition of 10 eq. TATP **1** and a 49700-fold excess of TFA. Plotting $\ln(A_t/A_0)$ against time gives a rate constant of $k=1.6\cdot10^{-2}$ s^{-1}. The measurements were carried out in dry and degassed dichloromethane at 25 °C. The spectra clearly indicate decomposition after a short induction period.

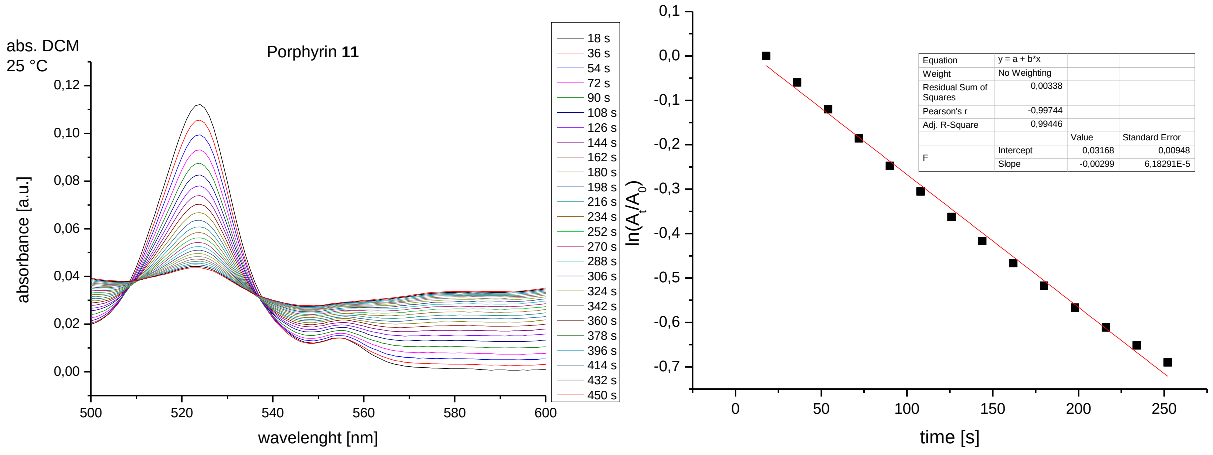

Figure S6. UV-spectra of porphyrin **11** after addition of 10 eq. TATP **1** and a 49700-fold excess of TFA. Plotting $\ln(A_t/A_0)$ against time gives a rate constant of $k=3.0\cdot10^{-3}$ s^{-1}. The measurements were carried out in dry and degassed dichloromethane at 25 °C.

6.2 Mechanistic studies for the detection of peroxide-based explosives based on UV measurements

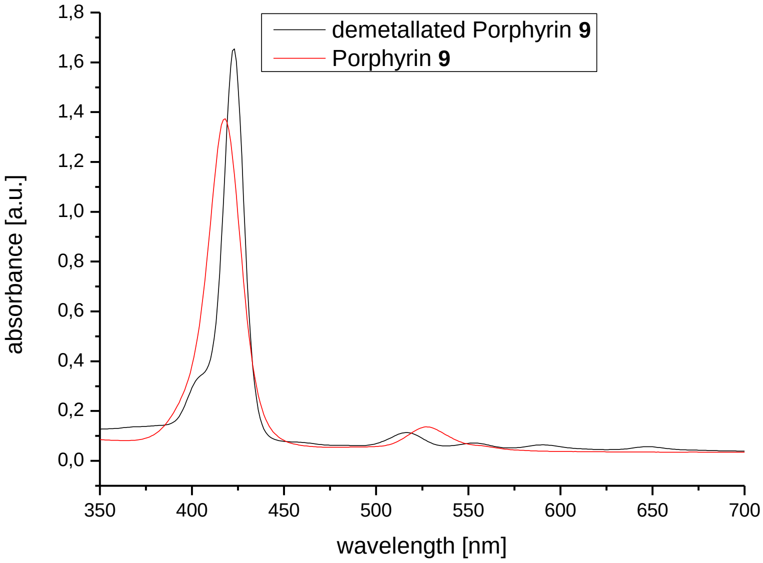

Figure S7. UV spectra of the Ni-porphyrin **9** and of the corresponding free base (demetallated) porphyrin. [5,10,15,20-Tetrakis(3,4,5-trimethoxyphenyl)porphyrinato]nickel(II) (**9**) (c = 5.00 µM) (black) and 5,10,15,20-tetrakis-(3,4,5-trimethoxyphenyl)-21*H*,23*H*-porphyrin (c = 5.00 µM) (red). The comparison of the two spectra indicates that a demetallation of porphyrin **9** (in the presence of TFA) would lead to a bathochromic shift of the Soret band. The measurements were carried out in dichloromethane at 25 °C.

6.3 Preparation of stock solutions for UV titration of 9 with different oxidants

A stock solution of the porphyrin **9** (5 µM) in DCM was prepared. 2 mL of the porphyrin stock solution was added to a cuvette and evaporated using a stream of N2. Subsequently, 2 mL of perfluoropentanoic acid was added and the measurements were carried out. Also stock solutions of NH_4NO_3 (4.87 mM) and $Pb(OAc)_4$ (1 mM) in DCM were prepared and used for the measurements.

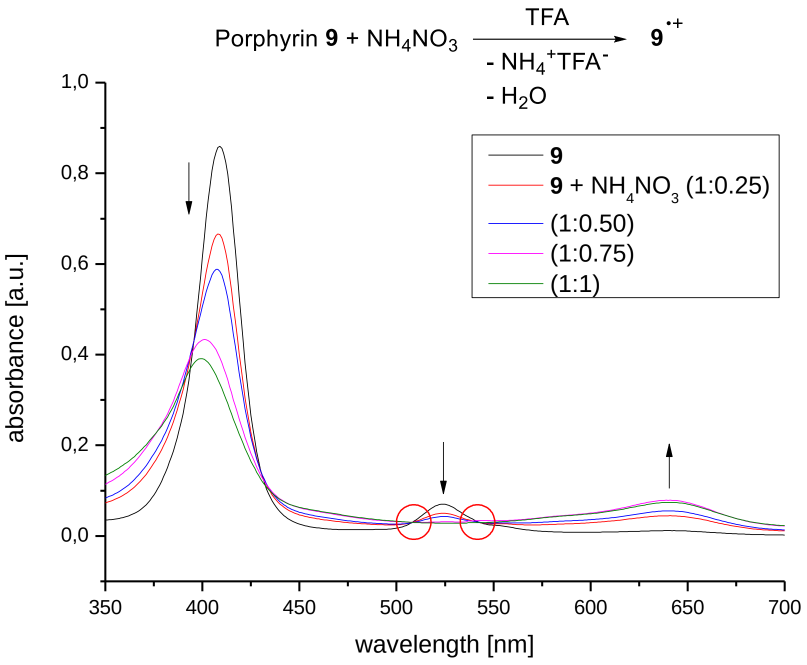

Figure S8. UV spectrum of porphyrin **9** before and after addition of different concentrations of NH_4NO_3. Up to a ratio of (porphyrin **9**)/NH_4NO_3 of 1:1 an isosbestic points (marked with circles) are observed and the typical spectrum of the π radical cation **9**$^{\cdot+}$ is forming. The measurements were carried out in perfluoropentanoic acid at 25 °C.

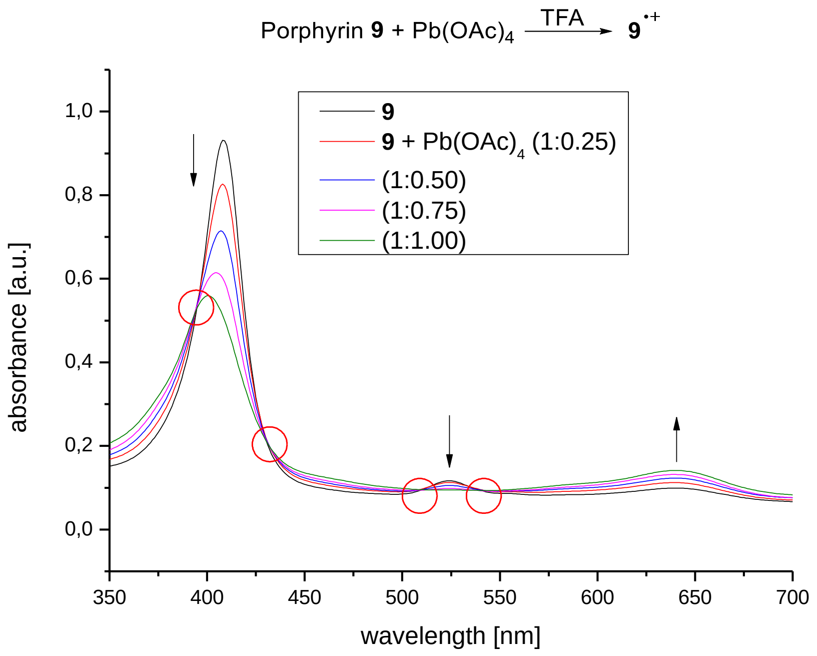

Figure S9. UV spectrum of porphyrin **9** before and after addition of different concentrations of $Pb(OAc)_4$. Up to a ratio of (porphyrin **9**)/$Pb(OAc)_4$ of 1:1 isosbestic points are observed (marked with circles) and the typical spectrum of the π radical cation **9**$^{\cdot+}$ is forming. The measurements were carried out in perfluoropentanoic acid at 25 °C.

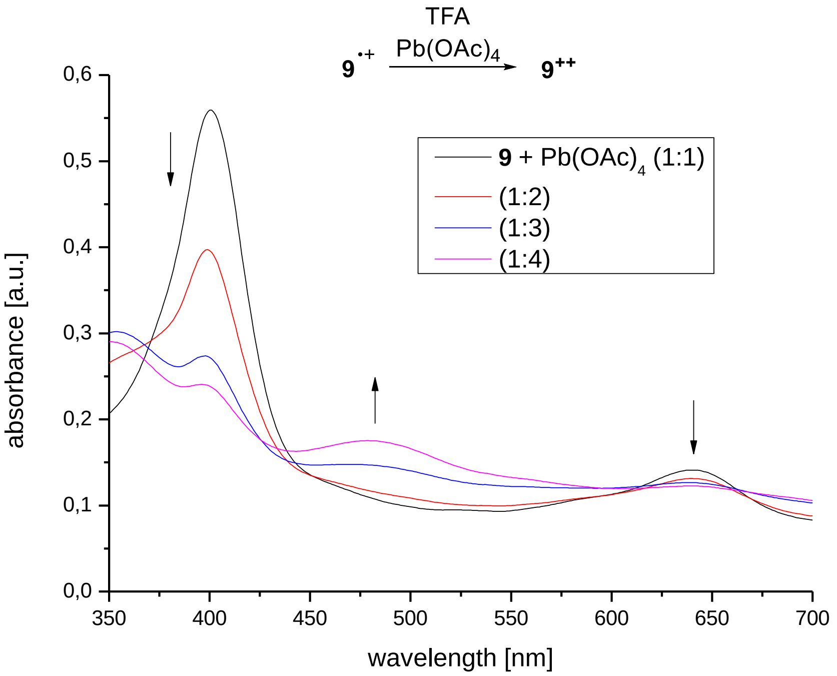

Figure S10. UV spectrum of porphyrin **9**$^{\cdot+}$ and the spectra after addition of different concentrations of $Pb(OAc)_4$. The addition of an excess of $Pb(OAc)_4$ leads to the formation of dication **9**$^{++}$, which is confirmed by the decrease of the broad band between 600-700 ppm, the formation of a broad shoulder between 450-550 ppm and by the decrease and hypsochromic shift of the Soret band. The measurements were carried out in perfluoropentanoic acid at 25 °C.

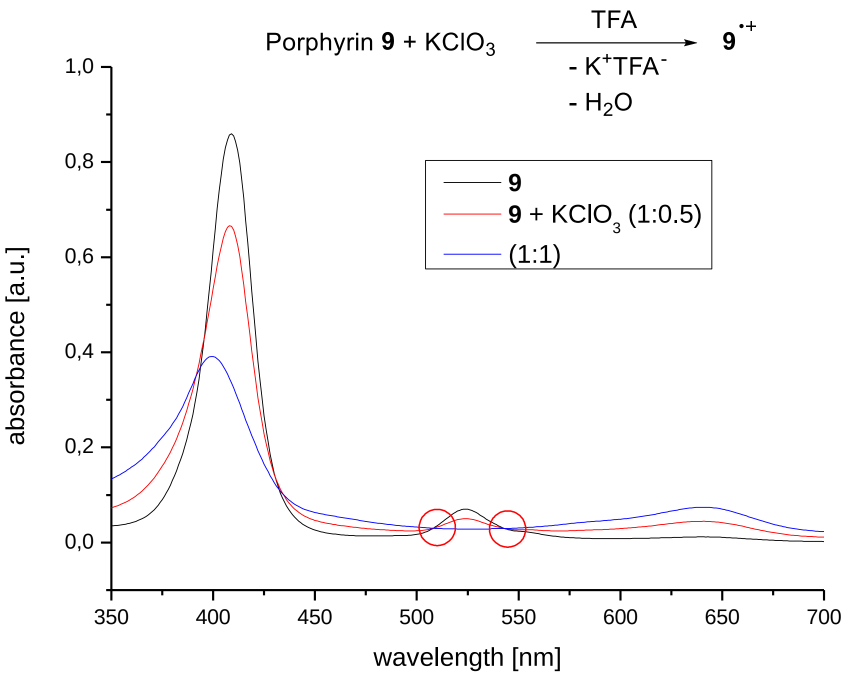

Figure S11. UV spectrum of porphyrin **9** and the spectra after addition of different concentrations of KClO3. Up to a ratio of (porphyrin **9**)/KClO3 of 1:1 isosbestic points are observed and the typical spectrum of the π radical cation $\mathbf{9}^{\cdot+}$ is forming. The measurements were carried out in perfluoropentanoic acid at 25 °C.

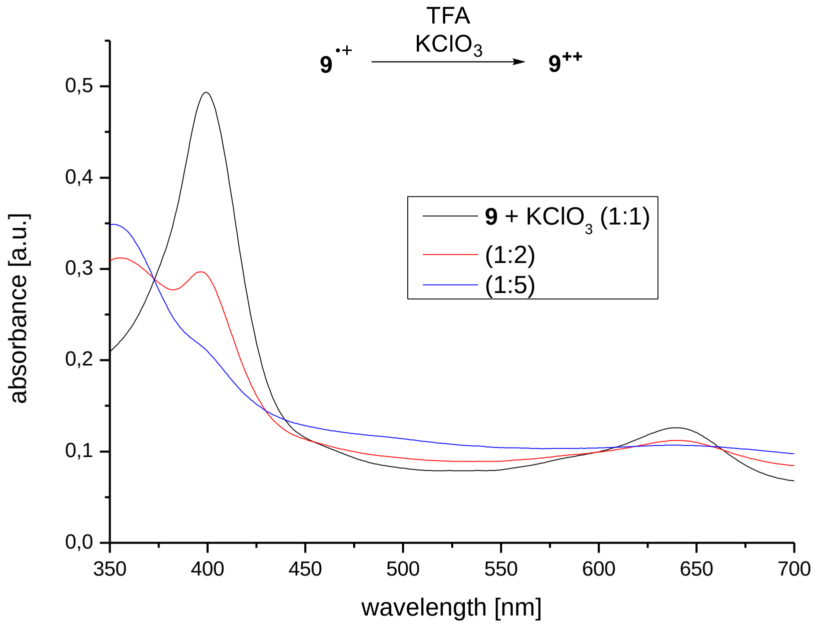

Figure S12. UV spectrum of porphyrin $\mathbf{9}^{\cdot+}$ and the spectra after addition of different concentrations of KClO3. The addition of an excess of KClO3 leads to the formation of dication $\mathbf{9}^{++}$, which is confirmed by the decrease of the broad band between 600-700 ppm, the formation of a broad shoulder between 450-550 ppm and by the decrease and hypsochromic shift of the Soret band. The measurements were carried out in perfluoropentanoic acid at 25 °C.

7. NMR spectra

7.1 Acid promoted decomposition of TATP **1**

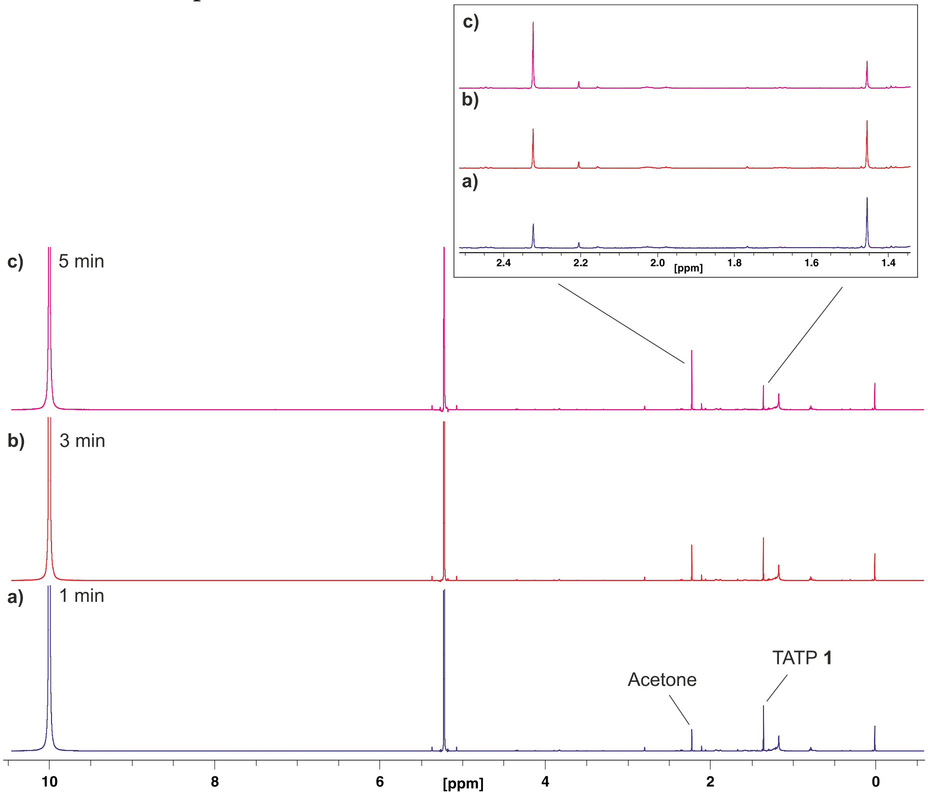

Figure S13. Kinetic measurements of the acidic decomposition of TATP **1** in CD_2Cl_2. Spectrum a) (blue) shows a 970 µM solution of TATP **1** in CD_2Cl_2 with a 1000-fold excess of TFA at 300 K after 1 minute. Spectrum b) (red) was recorded after 3 minutes (50% decomposition) and spectrum c) (pink) after 5 minutes (70% decomposition).

7.2 Mechanistic studies for peroxide-based explosives based on NMR measurements

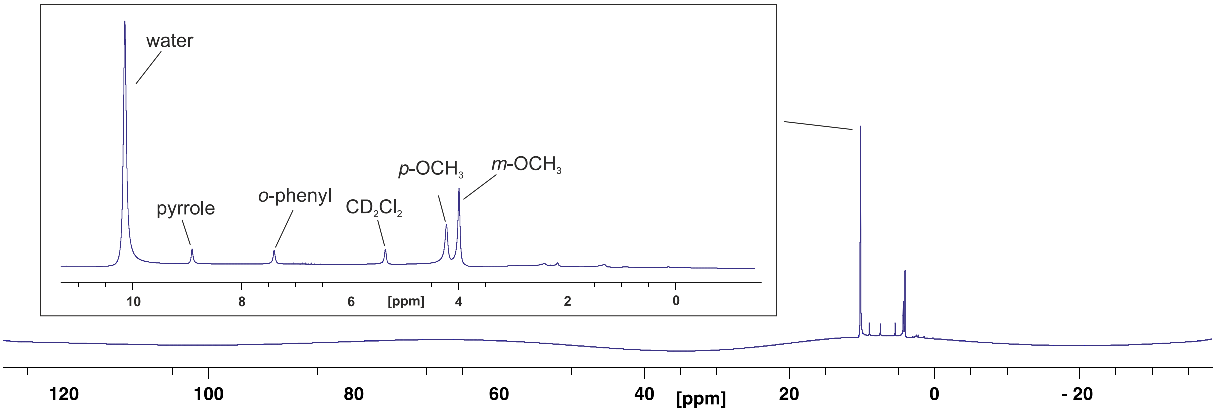

Figure S14. After the addition of a 1000-fold excess of TFA, no additional signals are observed in the range from 130 ppm to -35 ppm. No axial coordination of TFA to the nickel(II) ion occurs. Spectrum was measured in CD_2Cl_2 at 300 K.

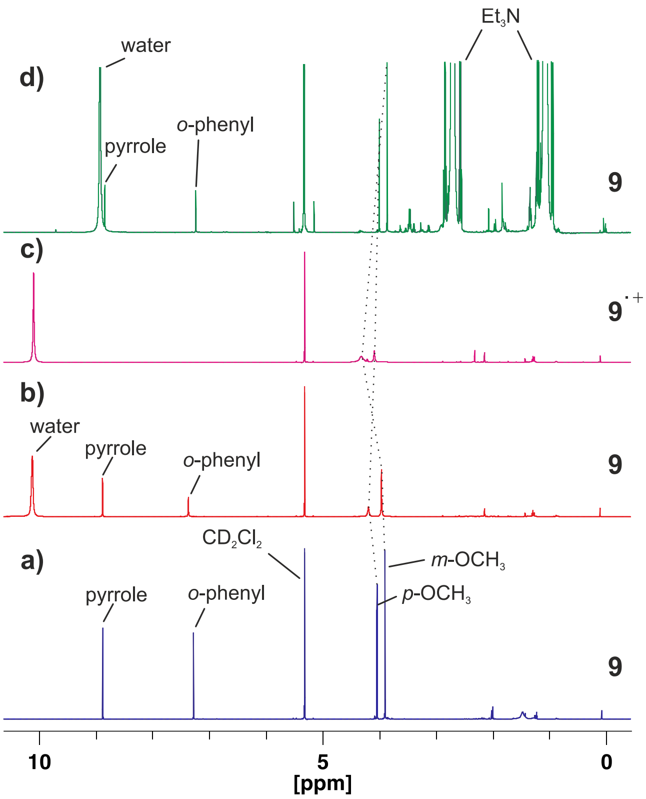

Figure S15. Full NMR spectra of porphyrin **9** (a), after successive addition of TFA (b), and TATP (c), and NEt_3 (d). Almost no change of the spectrum is observed after adding TFA to porphyrin **9** (spectrum b). Upon addition of TATP **1** the pyrrole and *o*-phenyl protons disappear, which is indicative of the formation of the radical cation **9**$^{\cdot+}$ (spectrum c). Further addition of NEt_3 restores the original spectrum of the neutral porphyrin **9** (spectrum d).

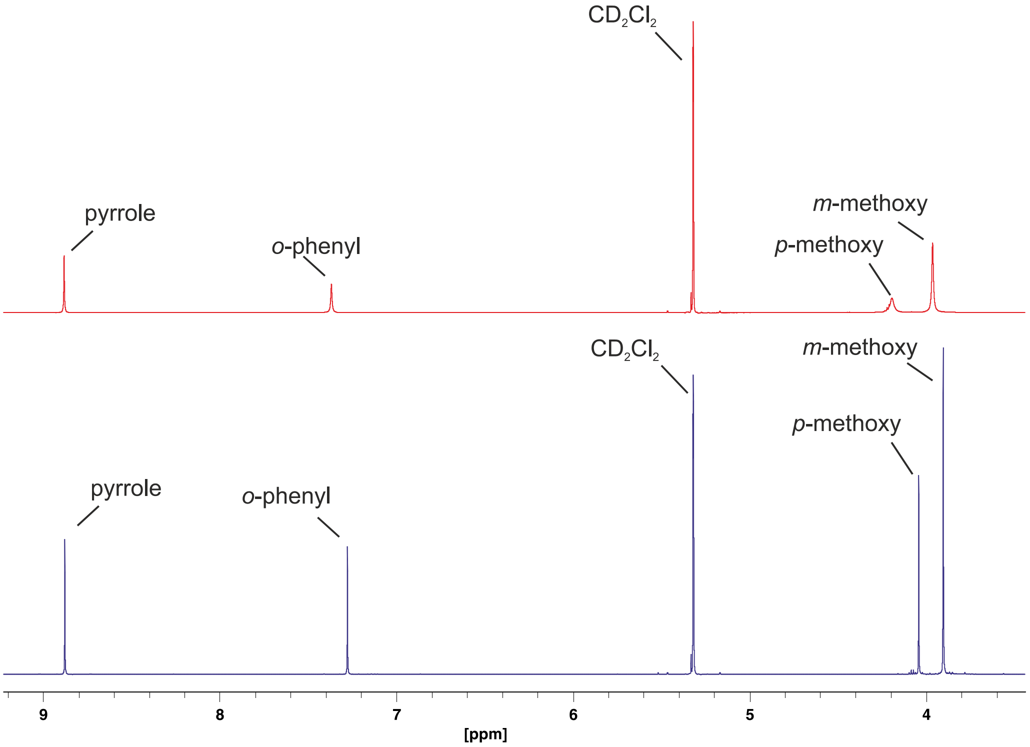

Figure S16. NMR spectra of Porphyrin **9** in CD_2Cl_2 (blue) and after the addition of a 1000-fold excess of TFA (red).

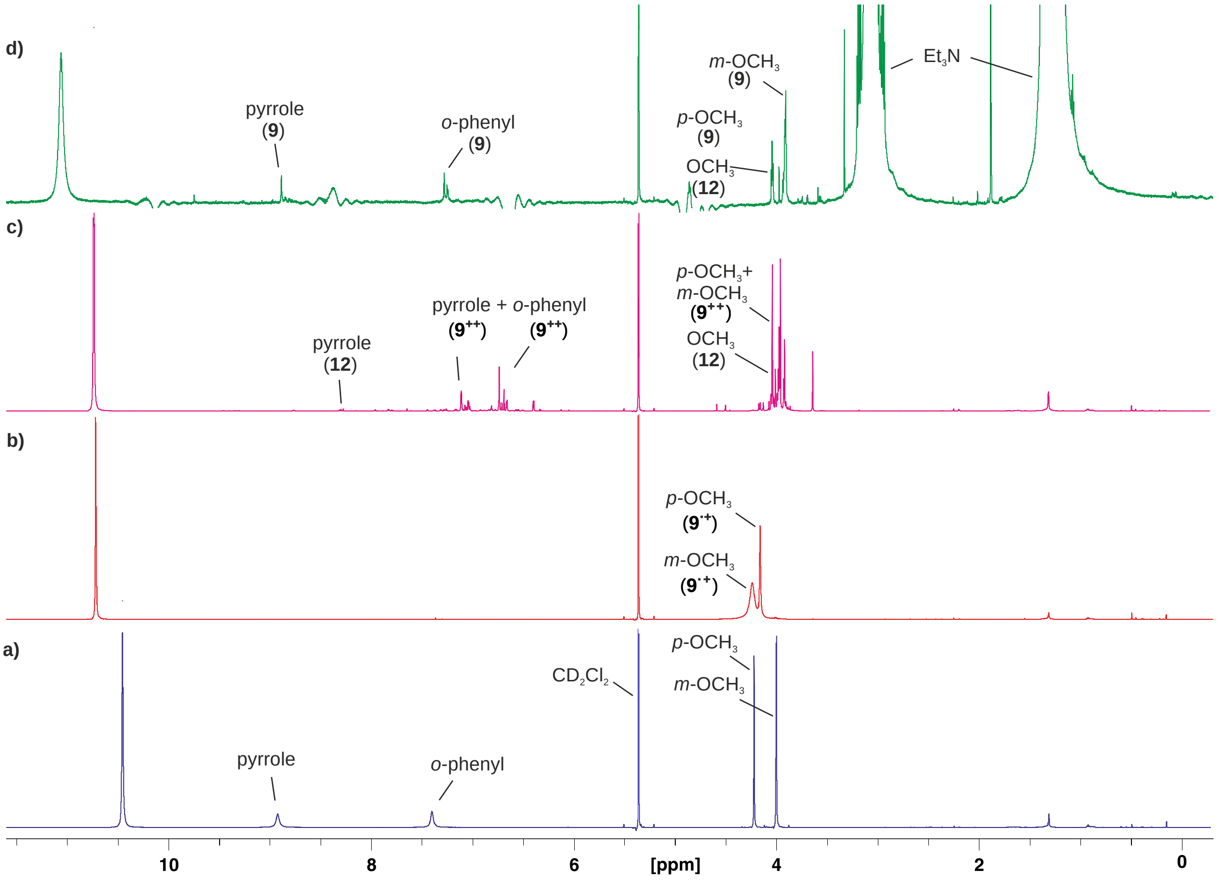

Figure S17. Full spectra of the change in NMR spectra after addition of TFA and NH_4NO_3. **a)→b)**: Upon addition of 0.2 eq. of NH_4NO_3 to a solution of **9** and TFA, the pyrrole and *o*-phenyl signals disappear and the *p*-methoxy protons are shifting high field and those of the *m*-methoxy protons low field, which is indicative of the formation of the radical cation $\mathbf{9}^{\cdot+}$. **b)→c)**: Further addition of NH_4NO_3 results in the formation of new asymmetric species, which is assigned to the dication $\mathbf{9}^{++}$. Furthermore another unsymmetric species is forming, which was identified as a species nitrated in β position (2-nitroporphyrin **12**). **c)→d)**: After the addition of triethylamine, the initial spectrum of **9** is recovered and only the signals for the β nitrated porphyrin **12** remain.

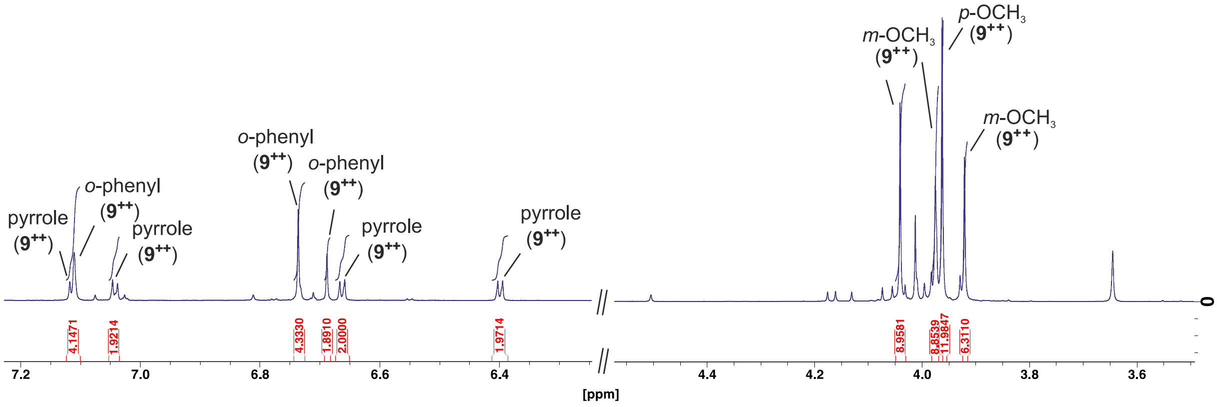

Figure S18. Shown is a zoom of spectrum c) of Figure S17.

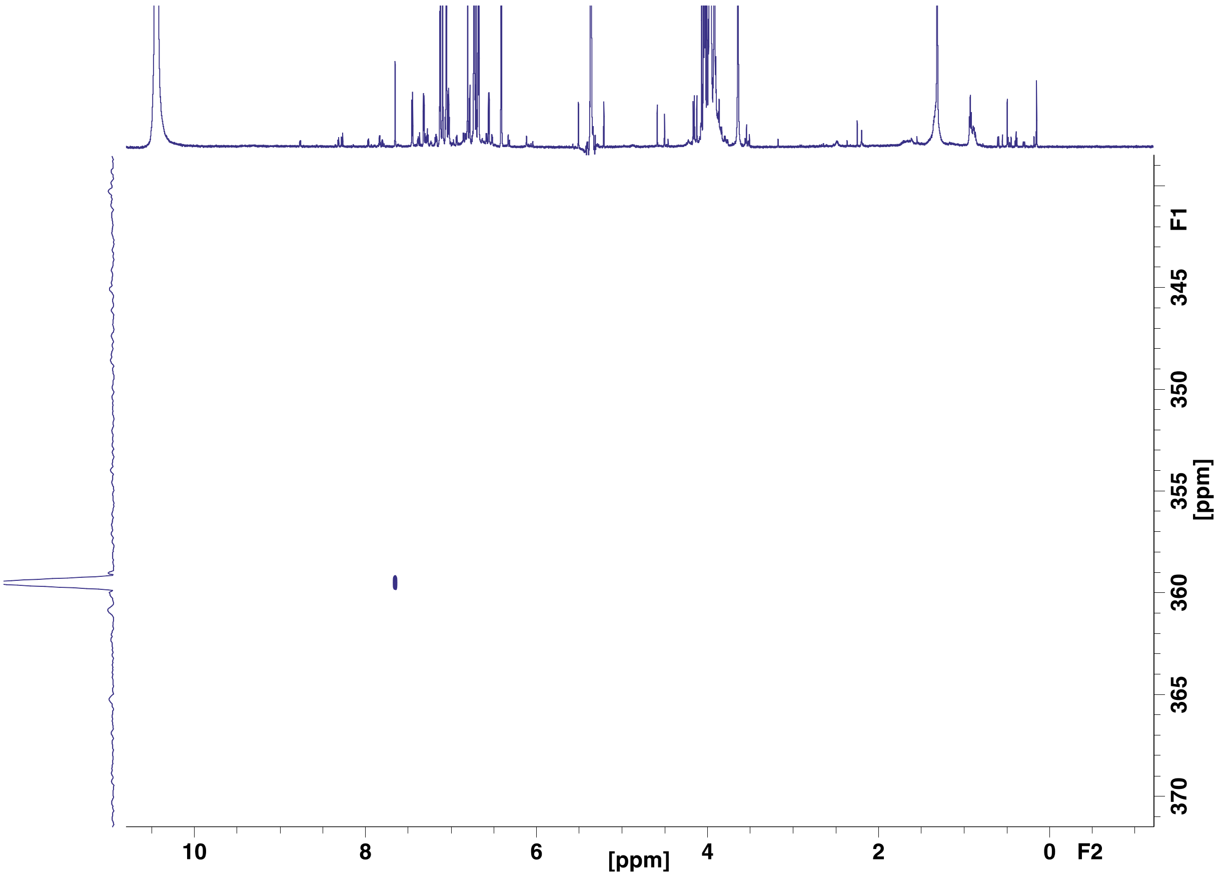

Figure S19. A signal at 359.5 ppm is coupling with a signal at 7.61 ppm in the ^{15}N-^{1}H HMBC spectra indicating the nitration in β position resulting in 2-nitroporphyrin **12**.

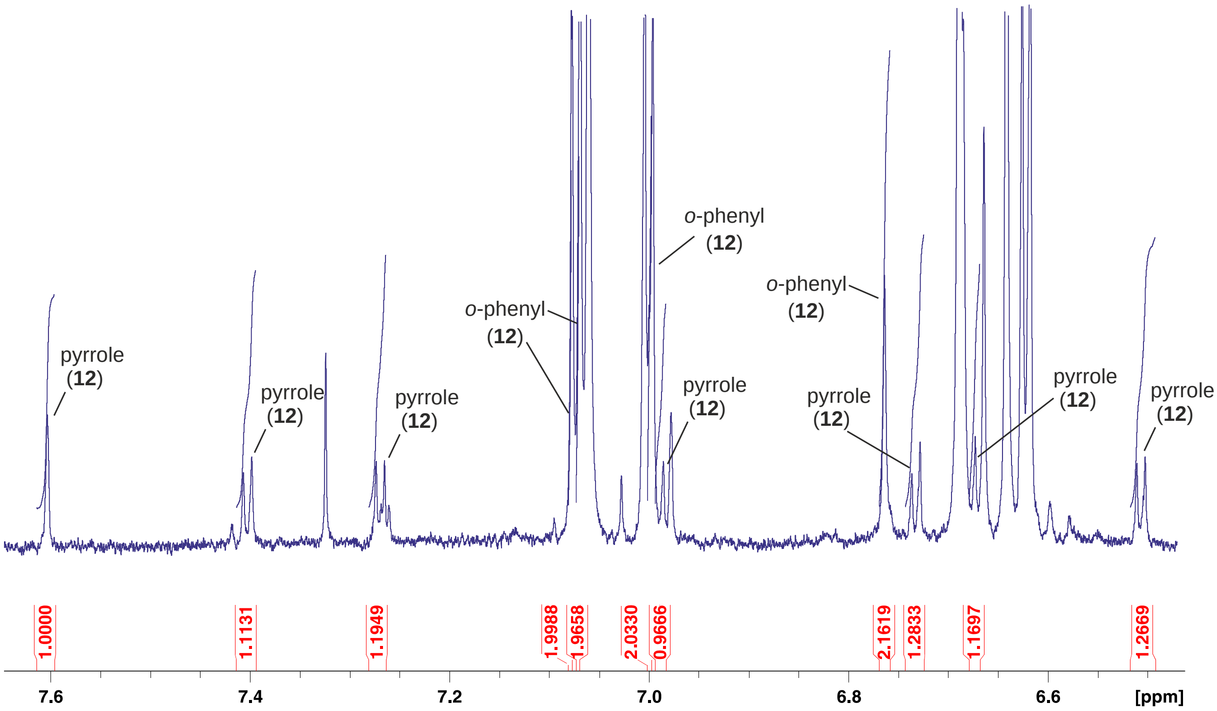

Figure S20. ^{1}H NMR spectrum of 2-nitroporphyrin **12**. As expected, 7 pyrrole protons and 8 ortho phenyl protons were found. The additional signals can be assigned to the dication **9**$^{++}$, which was formed as the main product.

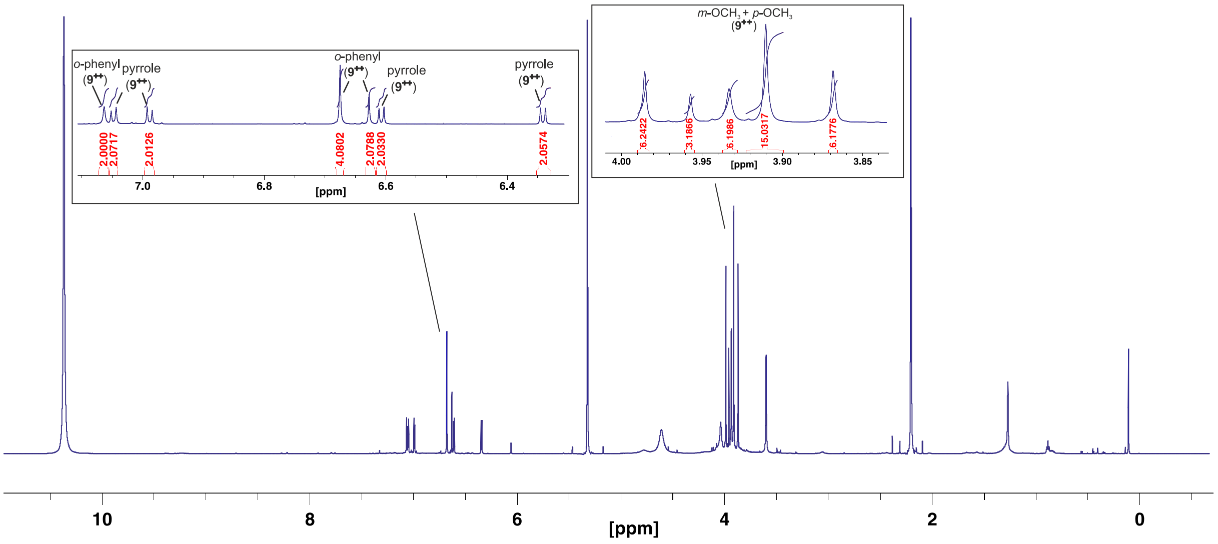

Figure S21. ^{1}H NMR spectrum of dication $\mathbf{9}^{++}$ prepared with large excess of TFA (49700-fold) and Pb(OAc)4 (1 equivalent with respect to porphyrin **9**). Four duplets each with an integral of two protons appear in the aromatic region (a total of eight protons), which can be assigned to the pyrrole protons. In addition, two singlets with an integral of two protons and one singlet with an integral of four protons (total of eight protons) are found, which can be assigned to the *o*-protons of the phenyl substituent. In the aliphatic region, five singlet with a total integral of 36 protons are visible.

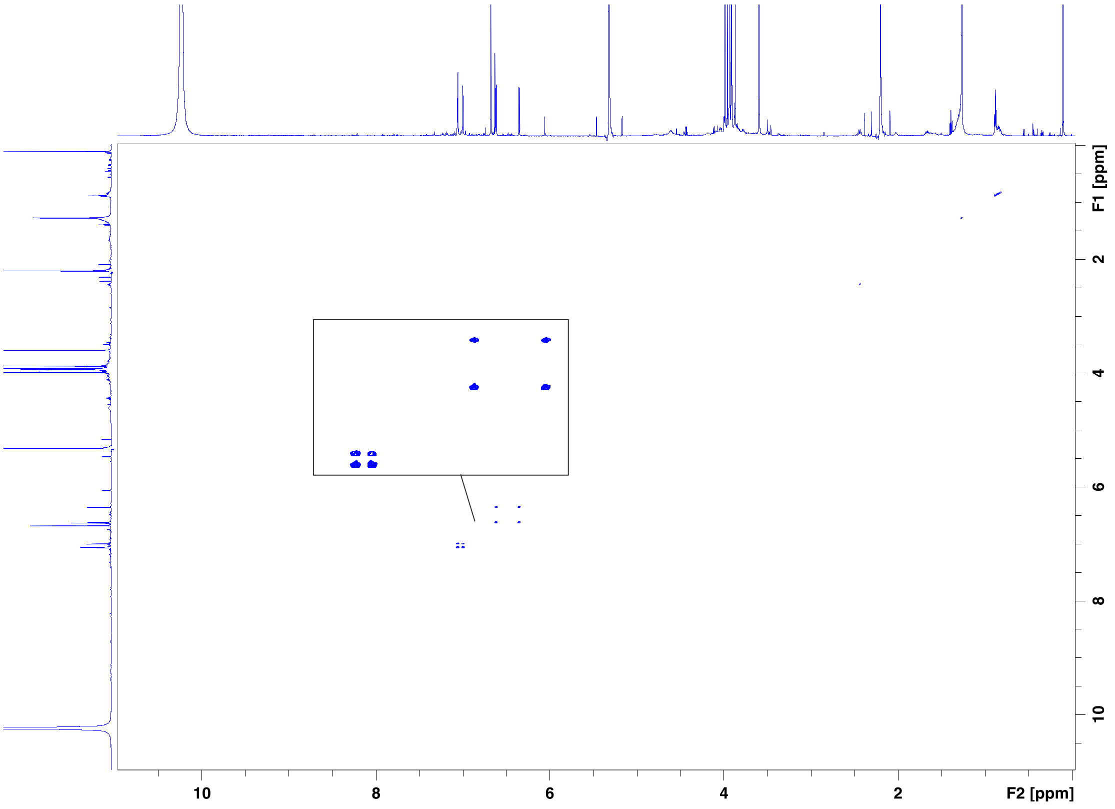

Figure S22. Shown is the COSY spectrum of the dication $\mathbf{9}^{++}$. Only the pyrrole protons couple with each other, suggesting that the protons at the pyrrole rings are chemically inequivalent, but there are two chemically equivalent pyrrole rings each.

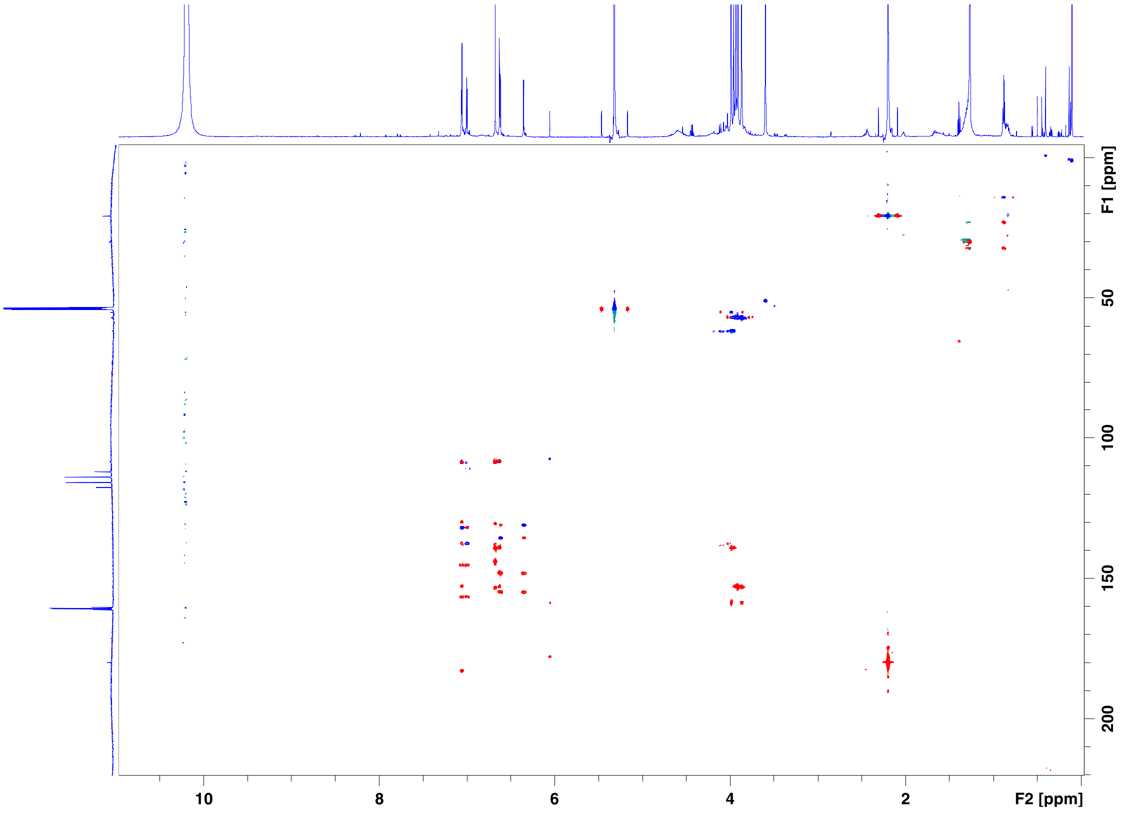

Figure S23. Shown is the HSQC (blue) and the HMBC (red) spectrum of the dication **9**$^{++}$. It can be clearly seen that our previously made assumption that the protons on one pyrrole are chemically inequivalent is correct.

NMR-analysis of the dication 9^{++}

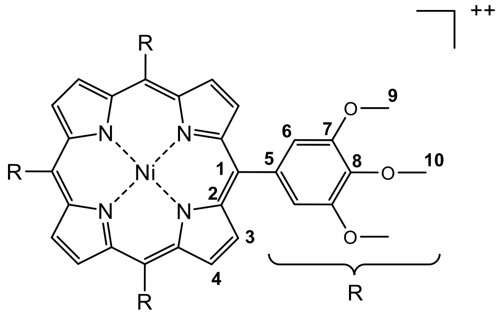

The atoms were numbered according to the scheme shown. Starting from the *meso* position of the porphyrin, the porphyrin was numbered first. The substituent at the *meso* position of the porphyrin followed in the manner indicated. The symmetry of the molecules was respected in the numbering even though not all signals are chemically equivalent.

The carbon atoms of the dication **9**$^{++}$ could be assigned via the heteronuclear single quantum coherence (HSQC) and the heteronuclear multiple bond correlation (HMBC) spectra (Figure S23).

^{1}H-NMR (500 MHz, $CDCl_3$, 300 K, TMS): δ = 7.06 (s, 2 H, *H*-6), 7.05 (d, ^3J = 5.0 Hz, 2 H, *H*-3/4), 6.99 (d, ^3J = 5.0 Hz, 2 H, *H*-3/4), 6.68 (s, 4 H, *H*-6), 6.63 (s, 2 H, *H*-6), 6.61 (d, ^3J = 4.9 Hz, 2 H, *H*-3/4), 6.34 (d, ^3J = 4.9 Hz, 2 H, *H*-3/4), 3.99 (s, 6 H, *H*-9/10), 3.96 (s, 3 H, *H*-9/10), 3.93 (s, 6 H, *H*-9/10), 3.91 (s, 15 H, *H*-9/10), 3.87 (s, 6 H, *H*-9/10) ppm.

^{13}C NMR (125 MHz, CD_2Cl_2, 300 K, TMS): δ = 156.7 (*C*-1/2), 154.9 (C-1/2), 153.3 (C-7/8), 153.0 (C-7/8), 152.9 (*C*-7/8), 148.2 (*C*-1/2), 148.0 (C-5), 145.3 (*C*-1/2), 144.0 (*C*-5), 139.2 (*C*-7/8), 139.1 (*C*-7/8), 137.6 (*C*-3/4), 135.6 (*C*-3/4), 132.0 (*C*-3/4), 131.1 (*C*-3/4), 130.5 (*C*-5), 130.0 (*C*-5), 108.8 (*C*-6), 108.7 (*C*-6), 108.5 (*C*-6), 108.3 (*C*-6), 61.9 (*C*-9/10), 61.9 (*C*-9/10), 57.7 (*C*-9/10), 57.0 (*C*-9/10), 56.9 (*C*-9/10), 55.1 (*C*-9/10) ppm.

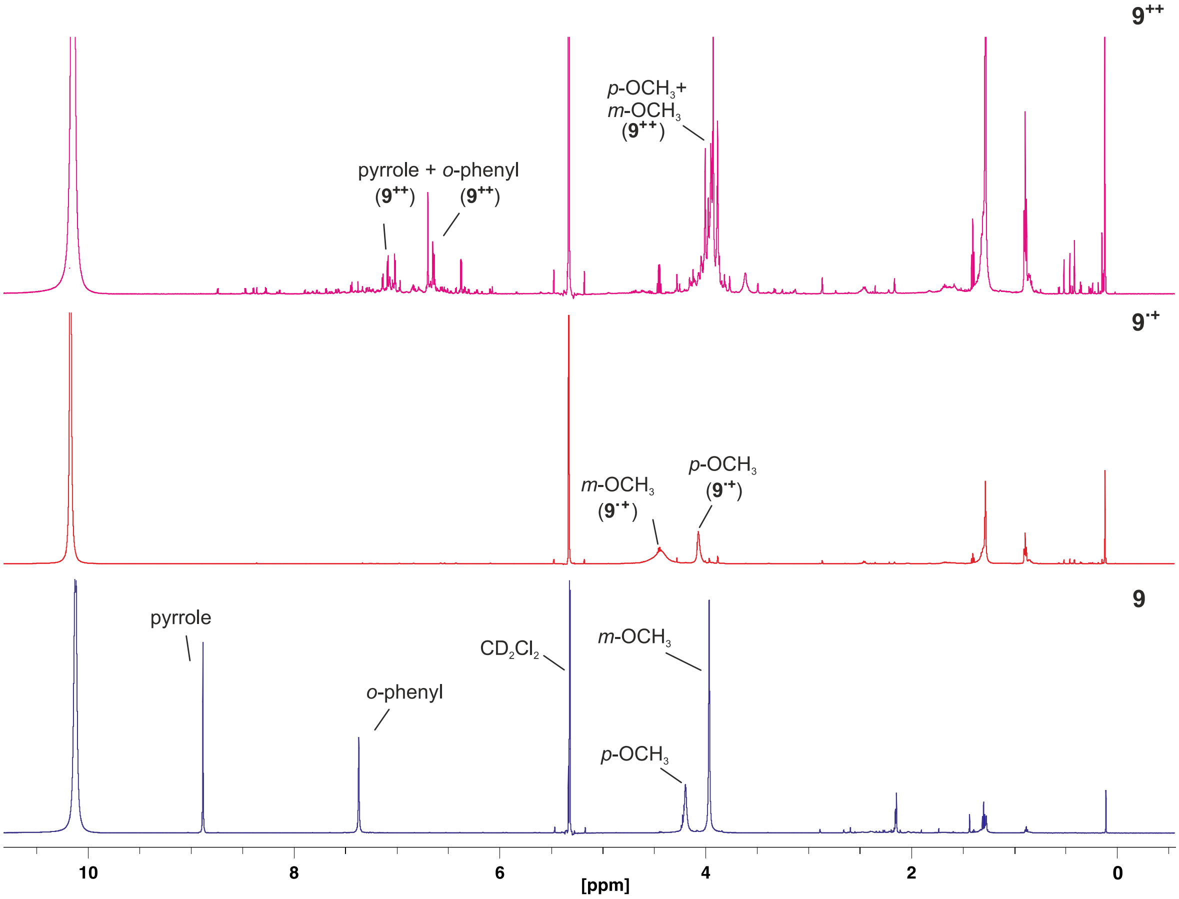

Figure S24. Full ^{1}H NMR spectra of **9** after addition of TFA (blue) and $KClO_3$ (one equivalent) (red). At first the radical cation $\mathbf{9}^{\cdot+}$ is forming and after further addition of $KClO_3$ the dication $\mathbf{9}^{++}$ forms (pink).

8. CV and Spectroelectrochemistry

8.1 Experimental

Voltammetric measurements were performed on an Autolab PGSTAT12 potentiostat (Eco Chemie BV, The Netherlands) connected to a PC running the software package Nova 2.1.4 (Metrohm, The Netherlands). For all measurements, 0.1 $molL^{-1}$ tetrabutylammonium perchlorate ($TBAClO_4$) in dry dichloromethane was used as the electrolyte. Cyclic voltammetry was performed with a Pt wire as a counter electrode, a Pt disc as a working electrode, and silver wire as a pseudo reference electrode, later referenced using ferrocene as the internal standard.

Spectro-electrochemistry was performed using previously reported homemade cell.[5] Briefly, Pt wire is used as a counter electrode, Pt mesh as a working electrode, and Ag wire as a pseudo reference electrode, Optical fibers were utilized as light guides to the cell and from the cell to the spectrometer. Calcium Fluoride windows are used and the light path is perpendicular to the working electrode. The cell contains fluidic connections for sample transfer to and from the cell, in addition, allowing for oxygen-free conditions.

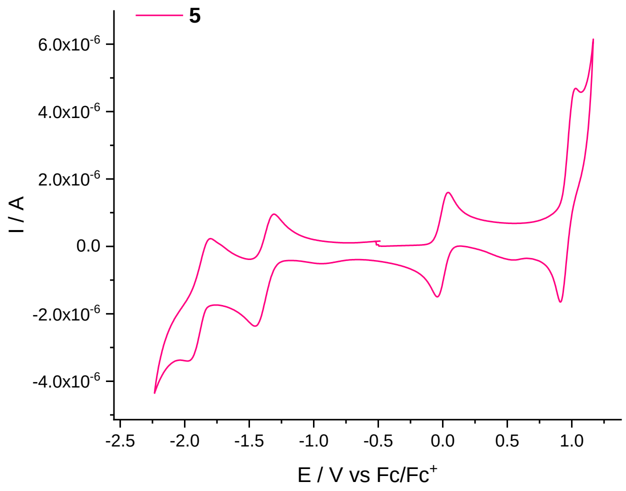

Figure S25. CV of **5** (0.1 mM) in DCM containing 0.1 M TFA and 0.1 M $TBAClO_4$ at a scan rate of 0.1 Vs^{-1}, Ferrocene is added as the internal reference.

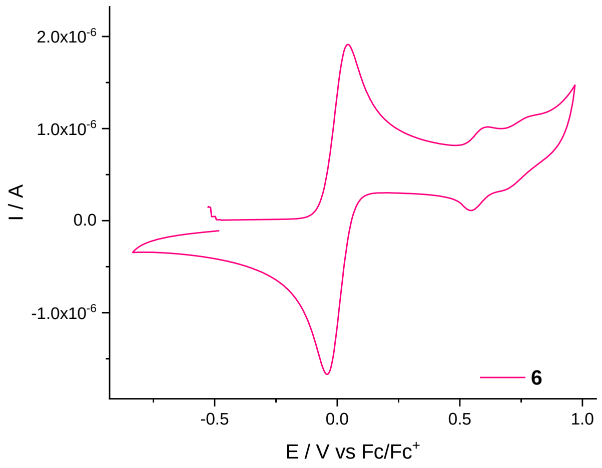

Figure S26. CV of **6** (0.1 mM) in DCM containing 0.1 M $TBAClO_4$ at a scan rate of 0.1 Vs^{-1}, Ferrocene is added as the internal reference.

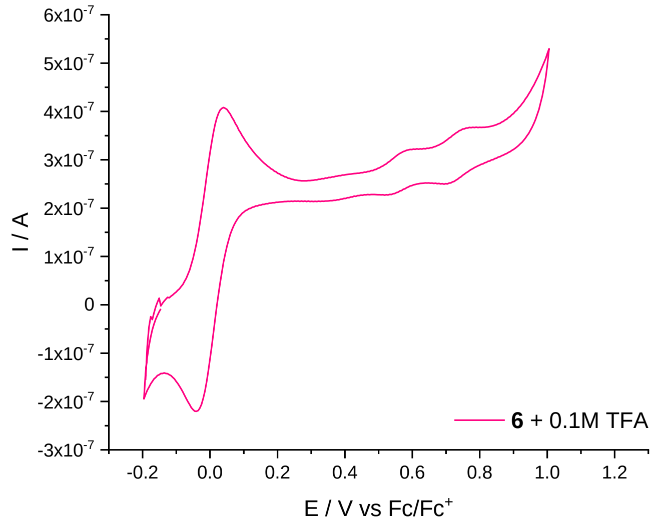

Figure S27. CV of **6** (0.1 mM) in DCM containing 0.1 M TFA and 0.1 M TBAClO4 at a scan rate of 0.1 Vs^{-1}, Ferrocene is added as the internal reference.

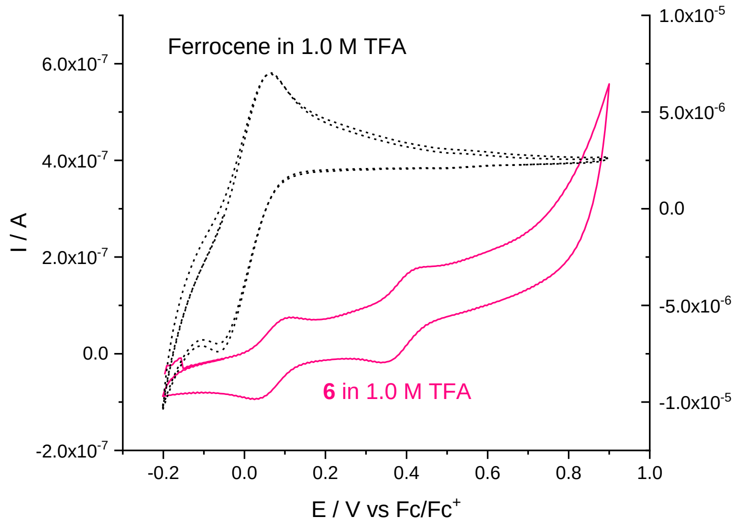

Figure S28. CV of **6** (0.1 mM) in DCM containing 1.0 M TFA and 0.1 M TBAClO4 at a scan rate of 0.1 Vs^{-1}, Ferrocene is used as the reference.

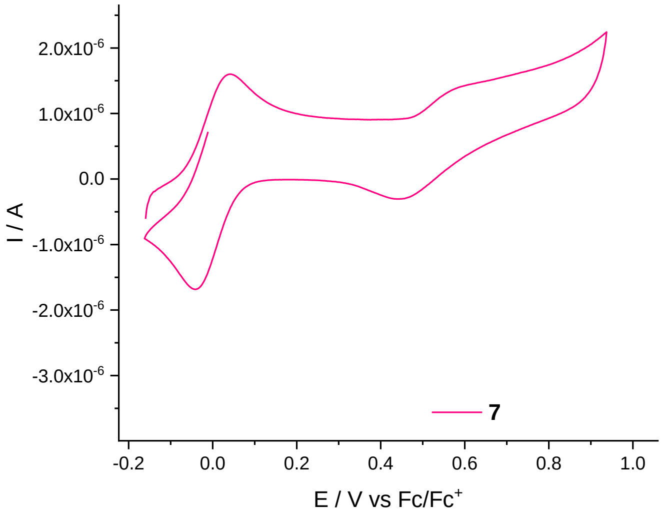

Figure S29. CV of **7** (0.1 mM) in DCM containing 0.1 M 0.1 M TBAClO$_4$ at a scan rate of 0.1 Vs^{-1}, Ferrocene is added as the internal reference.

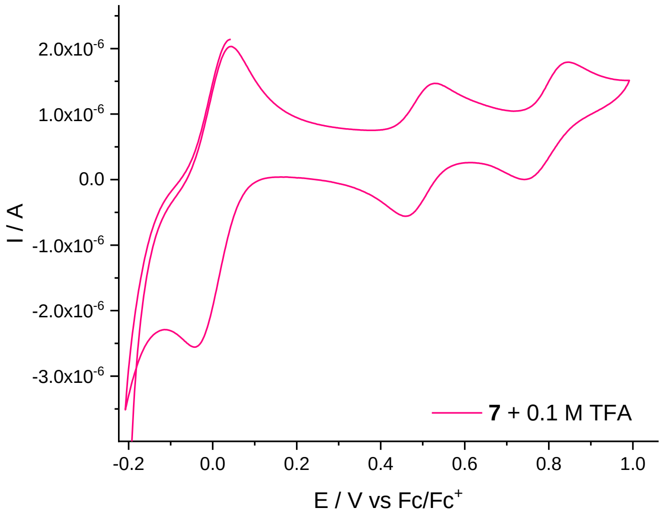

Figure S30. CV of **7** (0.1 mM) in DCM containing 0.1 M TFA and 0.1 M TBAClO$_4$ at a scan rate of 0.1 Vs^{-1}, Ferrocene is added as the internal reference.

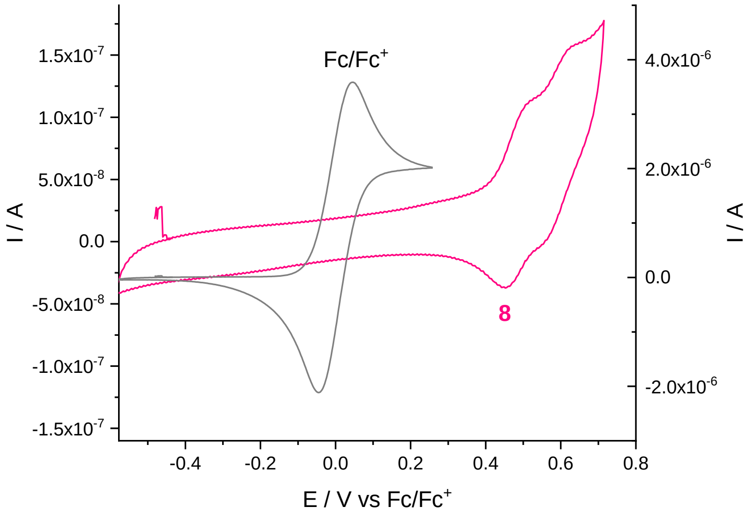

Figure S31. CV of **8** (0.1 mM) in DCM containing 0.1 M TBAClO4 at a scan rate of 0.1 Vs^{-1}, Ferrocene is added as the internal reference.

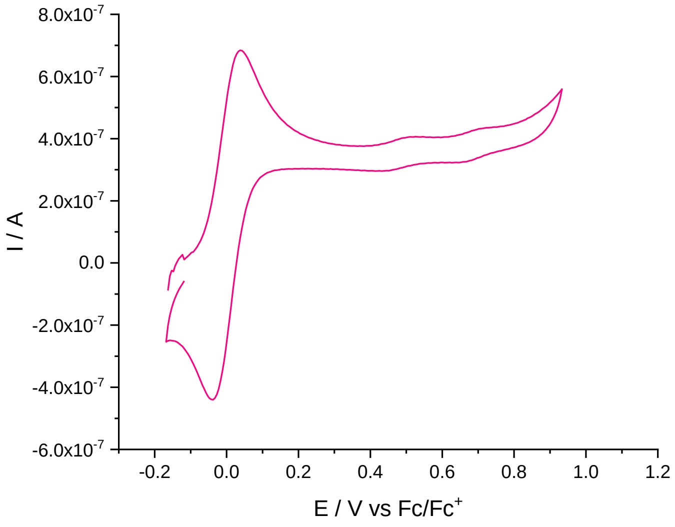

Figure S32. CV of **8** (0.1 mM) in DCM containing 0.1 M TFA and 0.1 M TBAClO4 at a scan rate of 0.1 Vs^{-1}, Ferrocene is added as the internal reference.

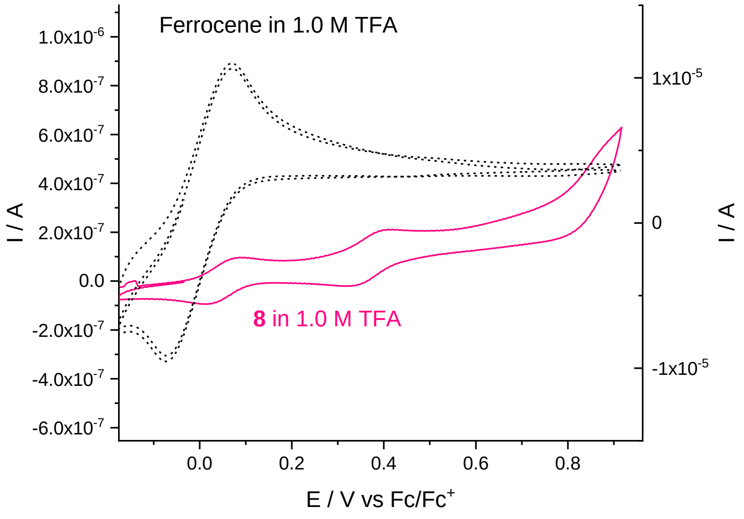

Figure S33. CV of **8** (0.1 mM) in DCM containing 1.0 M TFA and 0.1 M TBAClO4 at a scan rate of 0.1 Vs^{-1}, Ferrocene is used as the reference.

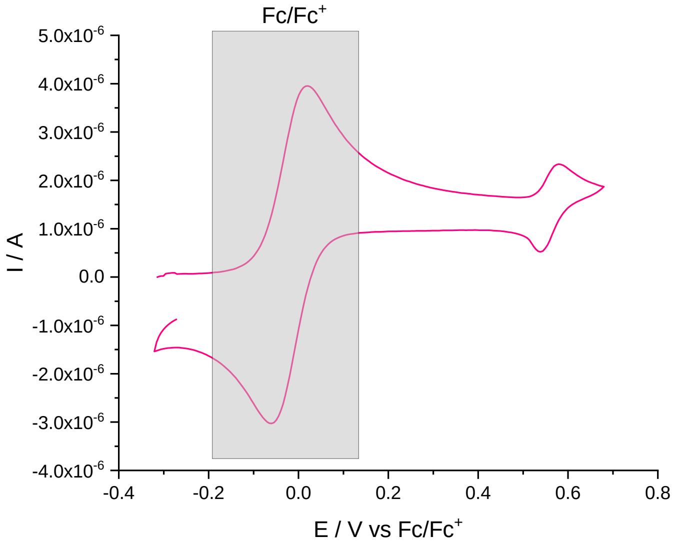

Figure S34. CV of **9** (0.1 mM) in DCM containing 0.1 M TBAClO4 at a scan rate of 0.1 Vs^{-1}, Ferrocene is added as the internal reference.

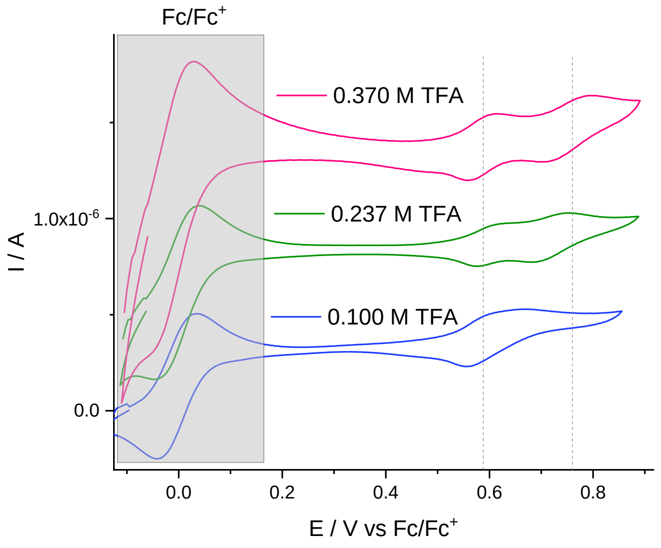

Figure S35. CV of **9** (0.1 mM) in DCM containing 0.1 – 0.37 M TFA and 0.1 M $TBAClO_4$ at a scan rate of 0.1 Vs^{-1}, Ferrocene is added as the internal reference.

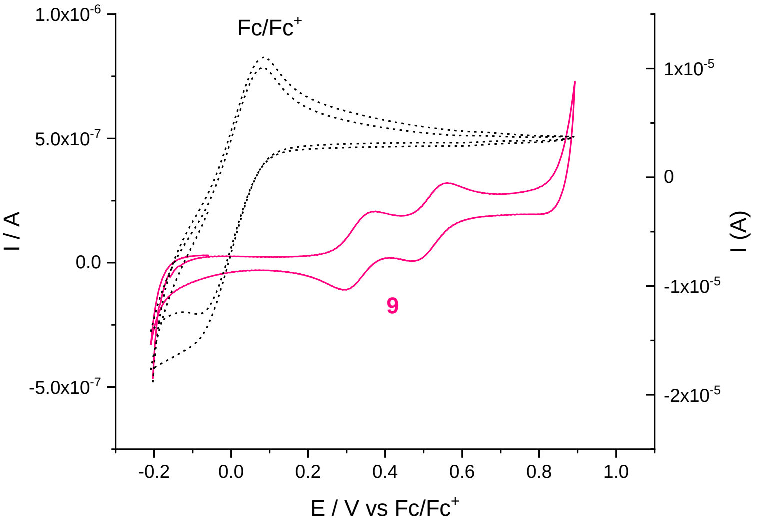

Figure S36. CV of **9** (0.1 mM) in DCM containing 1.0 M TFA and 0.1 M $TBAClO_4$ at a scan rate of 0.1 Vs^{-1}, Ferrocene is used as the reference.

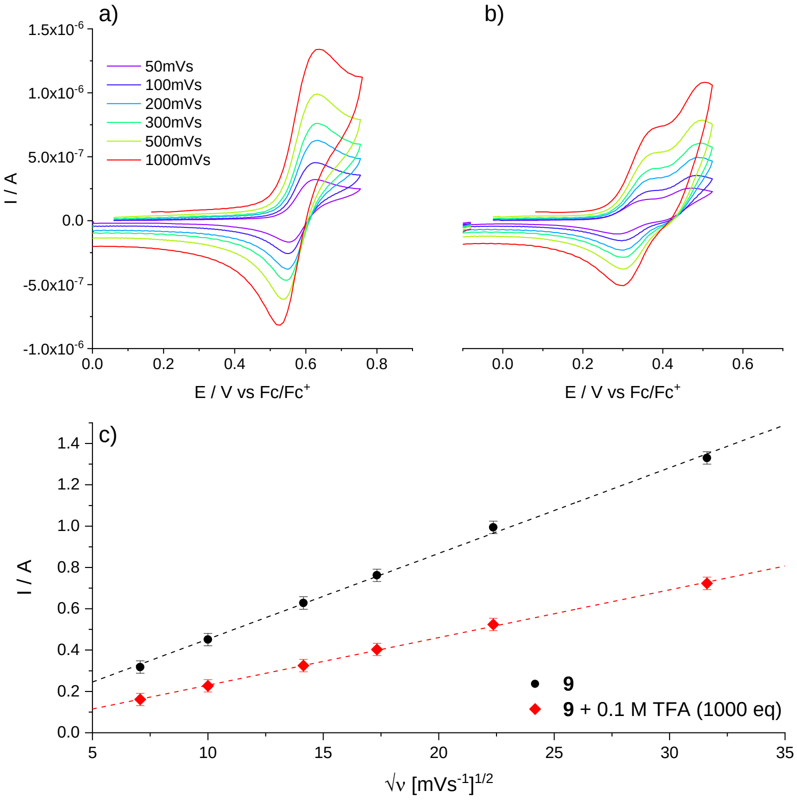

Figure S37. Cyclic voltammograms of **9** in DCM with 0.1 M of TBAClO4, referenced to Fc/Fc^{+} at varying scan rates a) without TFA present, b) with 0.1 M (1000 eq of TFA present), and c) Randles–Sevcik plot for the first oxidation of **9** with (red diamonds) and without (black circles) addition of TFA.

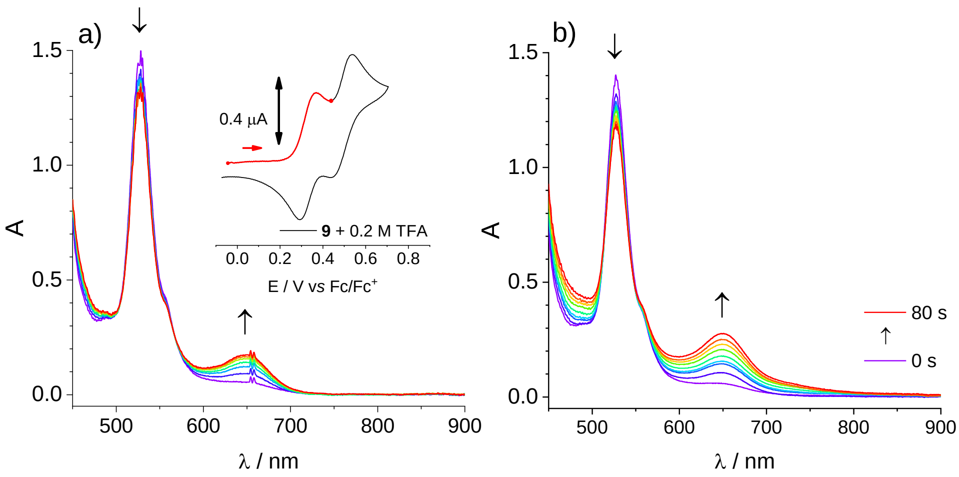

Figure S38. Spectroelectrochemical experiment of **9** (instrument artifact at 656 nm). Resulting in UV/vis spectra during the potential scan indicated in red on the CV presented as an insert. b) Potential controlled electrolysis (oxidation) of **9** in the presence of 0.2 M TFA (2000 eq) with 0.35 vs Fc/Fc^{+} applied to the working electrode.

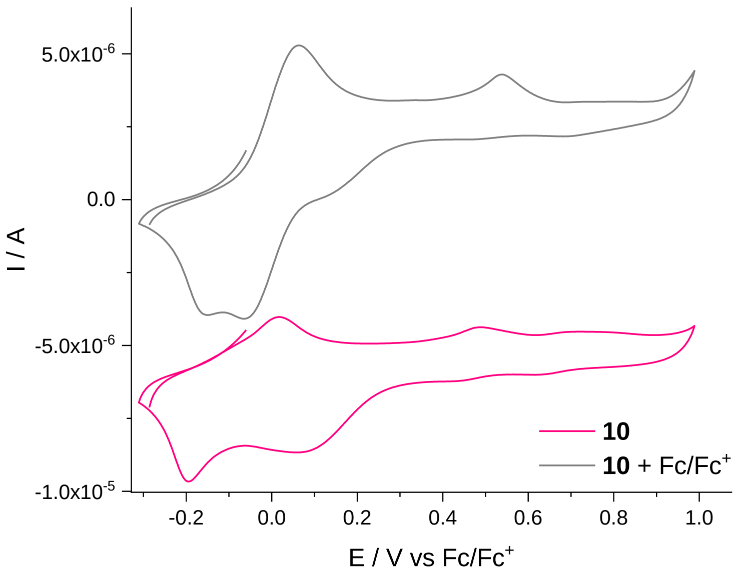

Figure S39. CV of **10** (0.1 mM) in DCM containing 0.1 M TBAClO4 at a scan rate of 0.1 Vs^{-1}, Ferrocene is added as the internal reference.

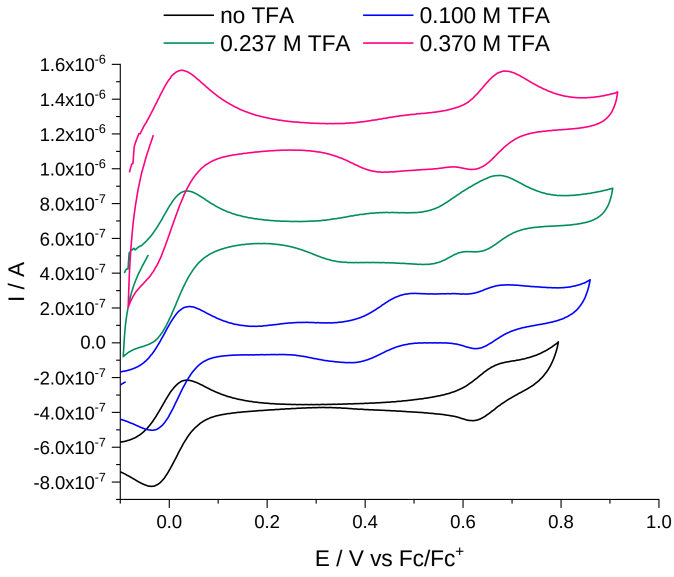

Figure S40. CV of **11** (0.1 mM) in DCM containing 0.1 – 0.37 M TFA and 0.1 M TBAClO4 at a scan rate of 0.1 Vs^{-1}, Ferrocene is added as the internal reference.

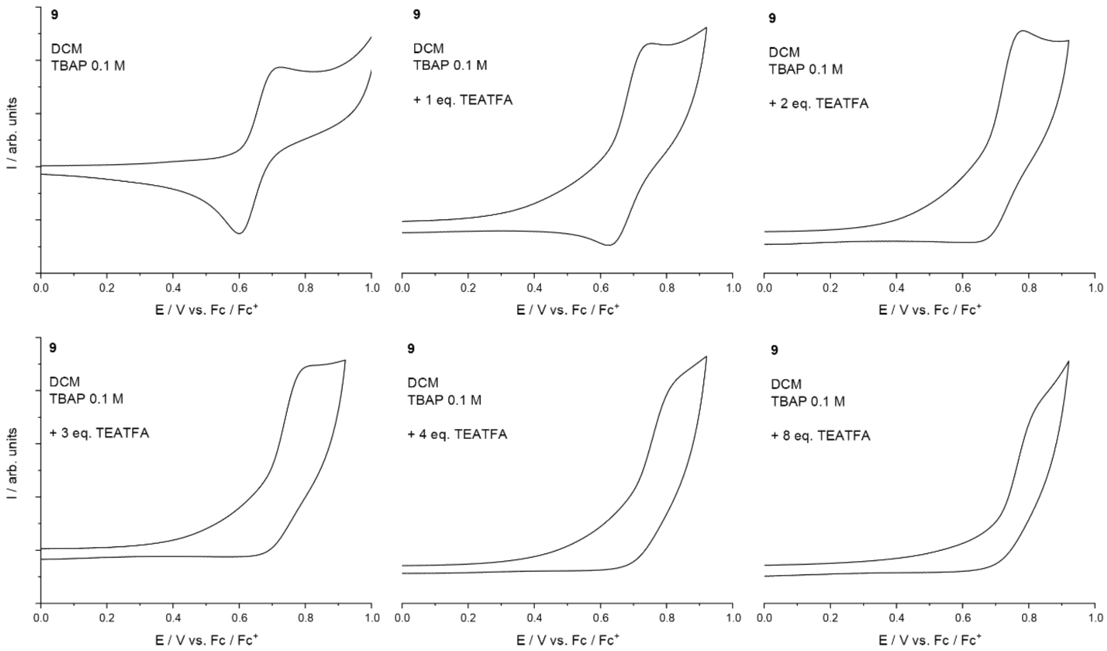

Figure S41. CVs of **9** (0.1 mM) in DCM and tetrabutylammonium perchlorate (0.1 M) upon the gradual addition of tetraethyl trifluoracetate (1– 8 equiv., see the amounts in the graphs).

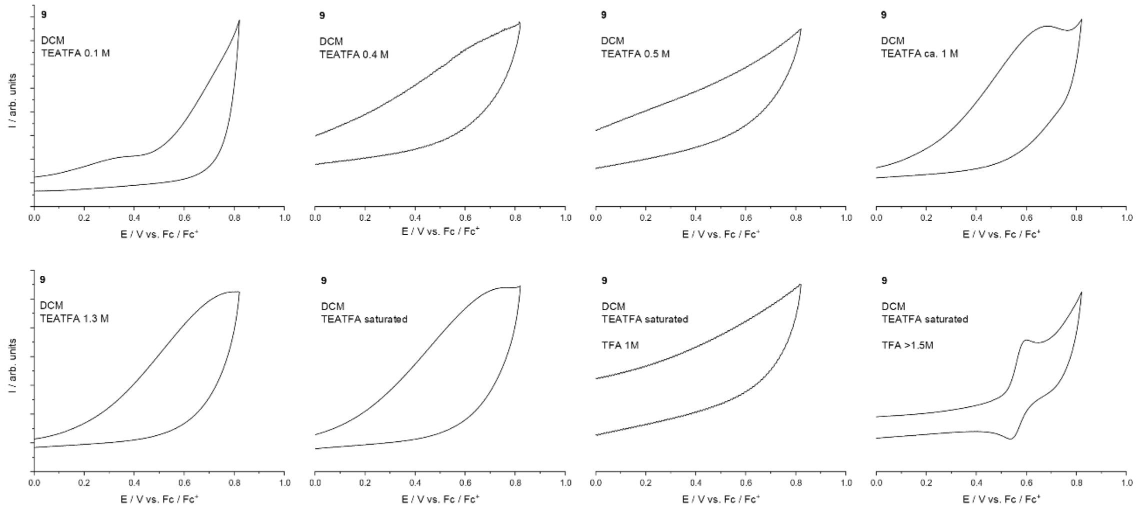

Figure S42. CVs of **9** (0.1 mM) in DCM with tetraethyl trifluoracetate as the supporting salt at increasing concentrations (0.1 M – 1.3 M and saturated solution, the concentrations are given in the graphs) and subsequent addition of TFA (1 and >1.5 M).

9. Computational Details

General molecular features:

Geometry optimizations were carried out on a PBE/def2-SVP[6–8] level of theory using ORCA[9]. Optimized geometries of the neutral porphyrin **9**, the radical-cation $\mathbf{9}^{\cdot+}$ and the dication $\mathbf{9}^{++}$ are shown below.

The extend to which ruffling and saddling is present in the fully optimized structures of neutral porphyrin, radical-cation and dication was evaluated using the NSD tool by Kinsbury and Senge[10] based on the method of Jenzen and Shellnutt.[11] Unfortunately, the definition of the terminology is inconsistent across the literature refereeing to the same deformation as either ruffling or saddling. We used the definition of the terms as discussed by Kinsbury and Senge in which ruffling is associated with the B_{1u} normal mode of the porphyrin core while the saddling motion is tied to the B_{2u} normal mode. The NSD distortions as well as a graphical representation of the obtained data is displayed in Tab. S3-8, Fig. S43-S46. Since a distortion of a geometry along normal coordinates is not immediately intuitive, we also use internal dihedrals Ψ for ruffling and X for saddling.

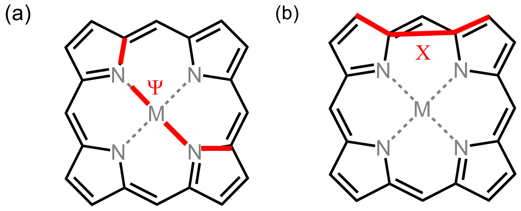

Figure S43. Graphical representation ruffling and saddling dihedral angles. (a) dihedral Ψ for ruffling (b) dihedral X for saddling.

Since the optimized structures exhibit C_2 symmetry the dihedral angle was averaged over all four saddling dihedrals (X) and two available ruffling dihedrals (Ψ).

Table S3. In-plane distortions for the fully optimized structure of the neutral porphyrin 9. NSD output in Å.

basis	Δ_{ip}	δ_{ip}	B_{2g}	B_{1g}	$E_u(x)$	$E_u(y)$	A_{1g}	A_{2g}
min.	0.34	0.01	0.00	0.00	0.00	0.00	-0.33	-0.04
ext.	0.36	0.00	0.00	0.00	0.00	0.00	-0.33	-0.04
			0.00	0.00	0.00	0.00	0.10	-0.11
total	0.38	0.00	0.00	0.00	0.00	0.00	-0.33	-0.04
			0.00	0.00	0.00	0.00	0.10	-0.11
			0.00	0.00	0.00	0.00	-0.02	0.00
			0.00	0.00	0.00	0.00	-0.09	-0.01
			0.00	0.00	0.00	0.00	0.01	-0.01
			0.00	0.00	0.00	0.00	0.03	
					0.00	0.00		
					0.00	0.00		
					0.00	0.00		
					0.00	0.00		
					0.00	0.00		
comp.	0.38	0.00	0.00	0.00	0.00	0.00	0.36	0.12

Table S4. Out-of-plane distortions for the fully optimized structure of the neutral porphyrin 9. NSD output in Å.

basis	Δ_{oop}	δ_{oop}	B_{2u}	B_{1u}	A_{2u}	$E_g(x)$	$E_g(y)$	A_{1u}
min.	1.87	0.00	0.69	-1.74	0.00	0.00	0.00	0.00
ext.	1.87	0.00	0.69	-1.74	0.00	0.00	0.00	0.00
			-0.10	0.00	0.00	0.00	0.00	0.00
total	1.87	0.00	0.69	-1.74	0.00	0.00	0.00	0.00
			-0.10	0.00	0.00	0.00	0.00	0.00
			0.00	-0.03	0.00	0.00	0.00	
						0.00	0.00	
						0.00	0.00	
comp.	1.87	0.00	0.69	1.74	0.00	0.00	0.00	0.00

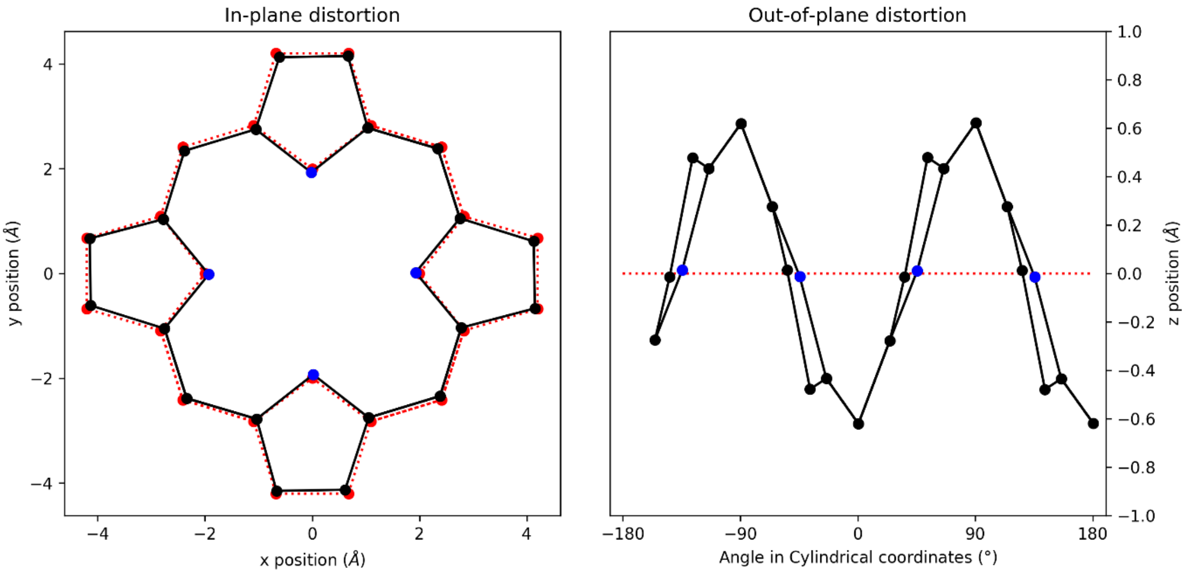

Figure S44. Graphical representation of in-plane and out-of-plane distortions for the fully optimized structure of the neutral molecule **9** generated by the NSD tool.

Table S5. In-plane distortions for the fully optimized structure of the radical-cation $9^{\cdot+}$. NSD output in Å.

basis	Δ_{ip}	δ_{ip}	B_{2g}	B_{1g}	$E_u(x)$	$E_u(y)$	A_{1g}	A_{2g}
min.	0.44	0.02	0.00	0.00	-0.01	-0.01	-0.43	0.07
ext.	0.49	0.00	0.00	0.00	-0.01	-0.01	-0.43	0.07
			0.00	0.00	0.00	0.00	0.10	0.20
total	0.51	0.00	0.00	0.00	-0.01	-0.01	-0.43	0.07
			0.00	0.00	0.00	0.00	0.10	0.20
			0.00	0.00	0.00	0.00	0.03	0.00
			0.00	0.00	0.00	0.00	-0.12	0.01
			0.00	0.00	0.00	0.00	0.01	0.03

			0.00	0.00	0.00	0.00	0.03	
					0.00	0.00		
					0.00	0.00		
					0.00	0.00		
					0.00	0.00		
					0.00	0.00		
comp.	0.51	0.00	0.01	0.00	0.01	0.01	0.46	0.22

Table S6. Out-of-plane distortions for the fully optimized structure of the radical-cation 9$^{\cdot+}$. NSD output in Å.

basis	Δ_{oop}	δ_{oop}	B_{2u}	B_{1u}	A_{2u}	$E_g(x)$	$E_g(y)$	A_{1u}
min.	2.23	0.01	-1.20	-1.89	0.00	0.00	0.00	0.00
ext.	2.24	0.00	-1.19	-1.89	0.00	0.00	-0.01	0.00
			0.16	0.00	0.00	-0.01	-0.01	0.00
total	2.24	0.00	-1.19	-1.89	0.00	0.00	-0.01	0.00
			0.16	0.00	0.00	-0.01	-0.01	0.00
			0.00	-0.03	0.00	0.00	0.00	
						0.00	0.00	
						0.00	0.00	
comp.	2.24	0.00	1.21	1.89	0.00	0.01	0.01	0.00

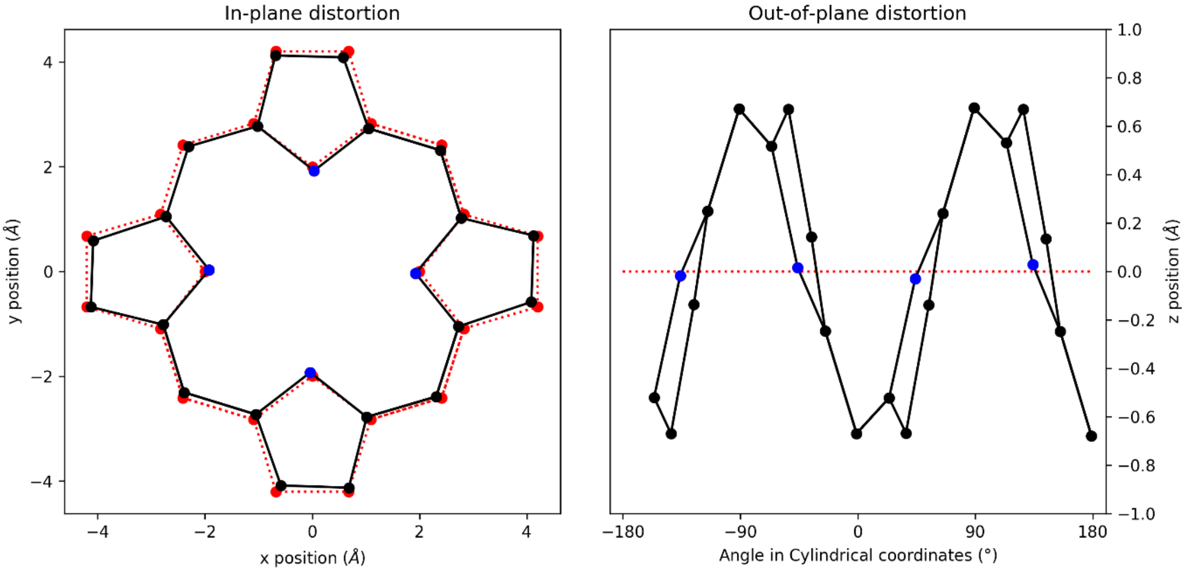

Figure S45. Graphical representation of in-plane and out-of-plane distortions for the fully optimized structure of the radical-cation **9**$^{\cdot+}$ generated by the NSD tool.

Table S7. In-plane distortions for the fully optimized structure of the dication 9^{++}. NSD output in Å.

basis	Δ_{ip}	δ_{ip}	B_{2g}	B_{1g}	$E_u(x)$	$E_u(y)$	A_{1g}	A_{2g}
min.	0.53	0.03	0.00	0.00	0.00	0.00	-0.52	0.10
ext.	0.60	0.01	0.00	0.00	0.00	0.00	-0.52	0.10
			0.00	0.00	0.00	0.00	0.17	0.23
total	0.62	0.00	0.00	0.00	0.00	0.00	-0.51	0.10
			0.00	0.00	0.00	0.00	0.17	0.23
			0.00	0.00	0.00	0.00	0.05	0.00
			0.00	0.00	0.00	0.00	-0.15	0.01
			0.00	0.00	0.00	0.00	0.01	0.02
			0.00	0.00	0.00	0.00	0.04	
					0.00	0.00		
					0.00	0.00		
					0.00	0.00		
					0.00	0.00		
					0.00	0.00		
comp.	0.62	0.00	0.00	0.00	0.00	0.00	0.57	0.26

Table S8. Out-of-plane distortions for the fully optimized structure of the dication 9^{++}. NSD output in Å.

basis	Δ_{oop}	δ_{oop}	B_{2u}	B_{1u}	A_{2u}	$E_g(x)$	$E_g(y)$	A_{1u}
min.	2.43	0.01	-1.14	-2.15	0.01	-0.01	-0.01	0.00
ext.	2.44	0.00	-1.14	-2.15	0.01	-0.01	-0.01	0.00
			0.21	-0.02	0.00	0.00	0.00	0.00
total	2.44	0.00	-1.14	-2.15	0.01	-0.01	-0.01	0.00
			0.21	-0.02	0.00	0.00	0.00	0.00
			0.00	-0.04	0.00	0.00	0.00	
						0.00	0.00	
						0.00	0.00	
comp.	2.44	0.00	1.16	2.15	0.01	0.01	0.01	0.00

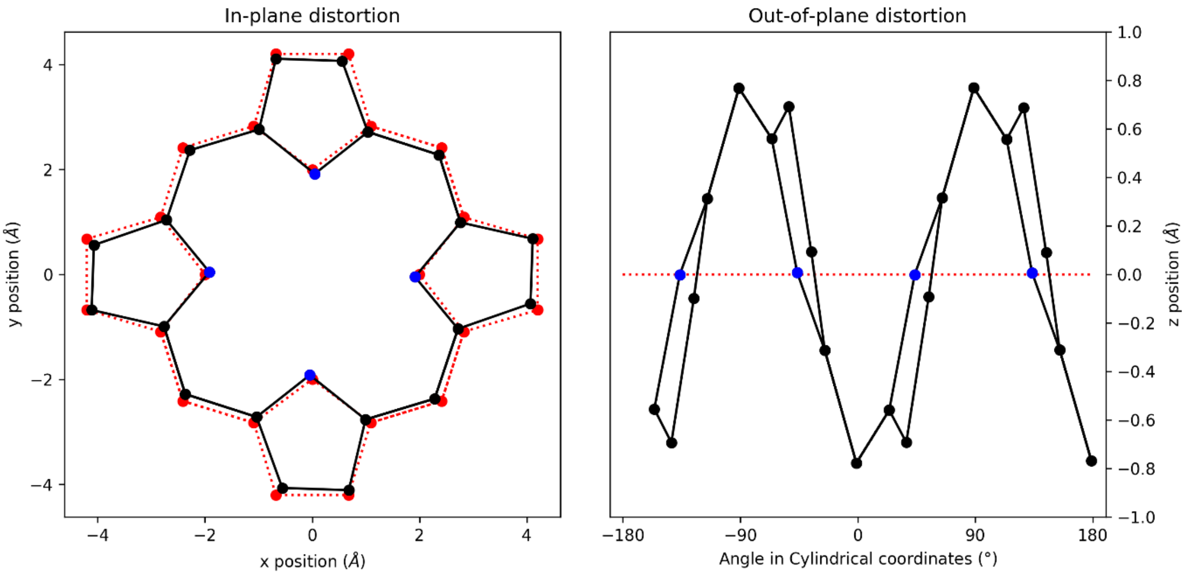

Figure S46. Graphical representation of in-plane and out-of-plane distortions for the fully optimized structure of the dication **9**$^{++}$ generated by the NSD tool.

ACID plots:

ACID Calculations were performed for the neutral porphyrin **9**, the radical-cation **9**$^{\cdot+}$ and the dication **9**$^{++}$ (PBE/def2-SVP//B3LYP/def2-TZVP level of theory.[6,12,13]) as well as for the parent Ni-porphin, Ni-porphyin radical cation and Ni-porphyrin dication (PBE/def2-SVP//B3LYP/6-31G* level of theory.[14–23]) using Gaussian 16.[24] Molecular orbitals with π-contribution were identified for each individual molecule and an orbital separated ACID calculation was performed. ACID plots are shown below (Figure S47-S50). The molecular orbitals included in the calculations are shown in Table S9-S10.

Table S9. ACID calculations for the parent, neutral Ni-porphin system, the radical-cation and the dication. MOs included in the orbital separated ACID plots.

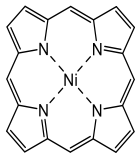

	neutral	radical-cation	dication
	93-84	94α-91α	93-84
	80	89α-83α	82
	74	80α	74
	68-67	74α-72α	65-63
	63	68α-67α	
		65α-63α	
MOs		93β-91β	
		89β-82β	
		89β-82β	
		75β	
		68β-67β	
		65β-63β	

Table S10. ACID calculations for the neutral porphyrin 9, the radical-cation $9^{\cdot+}$ and the dication 9^{++}. MOs included in the orbital separated ACID plots.

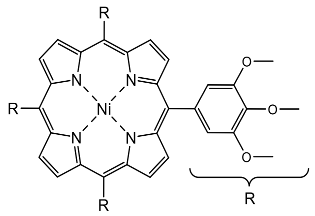

	neutral	radical-cation	dication
	270-245	270α-246α	269-251
	242-241	244α-237α	246-235
	212	229α-227α	232-224
	210	211α-209α	215-214
	207	205α	212
	204-203	202α	209-208
	200	196α-194α	
	197-196	189α	
	194	187α-186α	
	189	184α-181α	
	186	176α	
	176-169	174α	
MOs		269β-247β	
		245β-241β	
		239β-237β	
		231β-224β	
		211β-210β	
		202β	
		198β-195β	
		192β-191β	
		189β	
		187β	
		184β	
		181β	

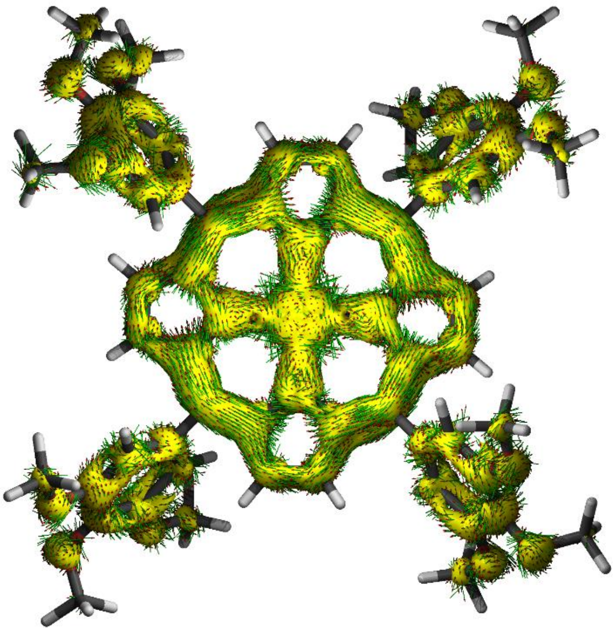

Figure S47. Orbital separated ACID plot for the neutral porphyrin **9**. Isovalue 0.06. Magnetic field vector B orthogonal to the ring plane, pointing towards the viewer.

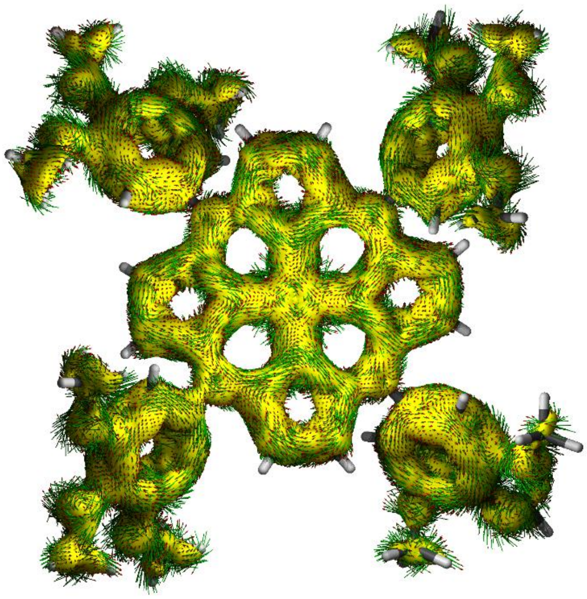

Figure S48. Orbital separated ACID plot for the radical-cation **9**$^{\cdot+}$. Isovalue 0.025. Magnetic field vector B orthogonal to the ring plane, pointing towards the viewer.

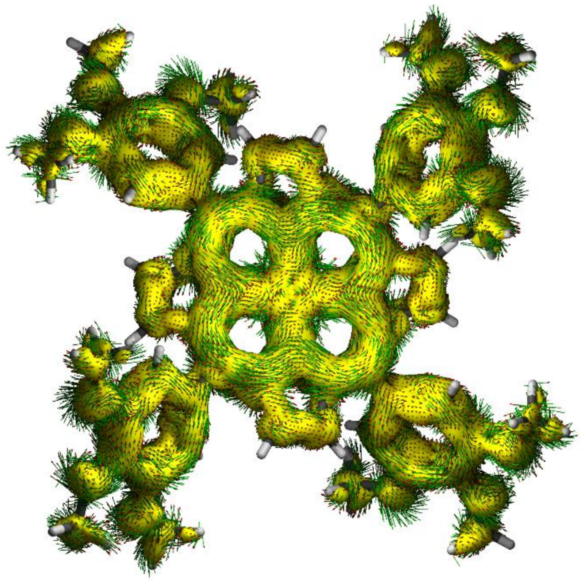

Figure S49. Orbital separated ACID plot for the core of dication **9**$^{++}$. Isovalue 0.25. Magnetic field vector B orthogonal to the ring plane, pointing towards the viewer.

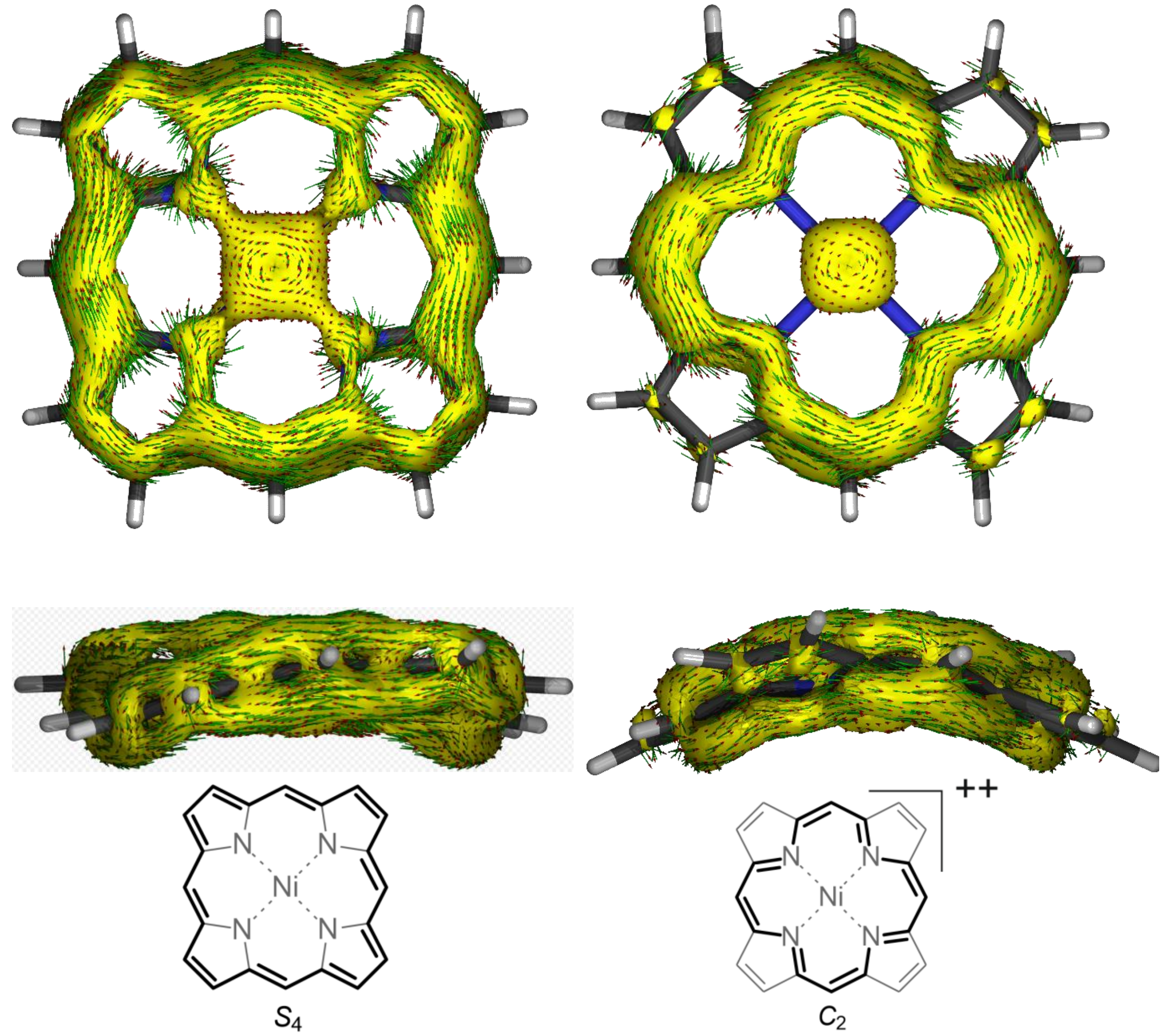

Figure S50. Orbital separated ACID plot for the unsubstituted, neutral Ni-porphin (left) and the corresponding dication (right), viewed from above (top) and side view (bottom). Computational details see text and Table S9.

NMR-Calculations:

NMR- Calculations were performed using ORCA. Geometry optimizations were performed using the PBE functional and the def2-SVP basis. To save CPU time, the NMR calculation of the neutral molecule **9** was restrained to a S_4 point group. The dication was optimized without symmetry restrictions. The chemical shifts were obtained by single point calculation on a M06-L/pcSseg2[25] level of theory with the grid setup as proposed by Stoychev.[26] Since the current ORCA version employs a newer set of grids, we followed the recommendation in the manual and used DEFGRID3 instead of GRID8 (Additional ORCA Keywords used: RIJCOSX def2/JK DEFGRID3 NOFINALGRIDX NMR AUTOAUX). TMS shifts were calculated as reference. The M06-L predicted ^{1}H-shifts which were reasonably close to the experimental data for both molecules. While the ^{13}C-shift prediction was also very close for the neutral molecule it was necessary to apply the TPSS[27] functional to improve ^{13}C-shift prediction for the dication. To match the experimentally observed NMR-Spectra calculated shifts were averaged using the Multiwfn tool.[28]

Table S11. Comparison of the experimental ^{13}C shifts of the neutral porphyrin 9 and the calculated values.

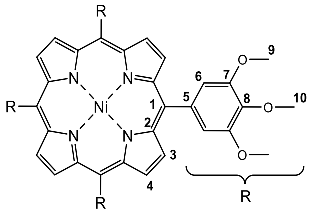

Numbering	experimental [ppm]	calculated*) [ppm]
C-1	119.0	118.5 (4C)
C-2	142.8	144.3 (4C)<br>142.1 (4C)
C-3/4	132.2	133.1 (4C)<br>132.6 (4C)
C-5	136.3	139.9 (4C)
C-6	111.9	123.7 (4C)<br>123.5 (4C)
C-7	151.6	156.2 (4C, C-7)<br>154.9 (4C, C-7)
C-8	137.9	144.7 (4C, C-8)
C-9/10	56.3 (*C*-9)<br>61.3 (*C*-10)	67.7-65.6 (12C)

Table S12. Comparison of the experimental ^{1}H shifts of the neutral porphyrin 9 and the calculated shifts.

numbering	experimental [ppm]	calculated[b*)] [ppm]
o-phenyl-*H*	7.28 (8H)	7.28 (8H)
pyrrole-*H*	8.86 (8H)	8.68 (8H)
m-methoxy-*H*	3.93 (24H)	3.73-4.89[a)]
p-methoxy-*H*	4.13 (12H)	3.62-4.61[a)]

a) note that there is almost free rotation of the methoxy groups, however, the calculations refer to a single conformation including H-bonds.
b) averaged from S_4 Symmetry
*) pbe/def2-SVP//M06-L/pcSseg-2

Table S13. Comparison of the experimental ^{13}C shifts of the dication 9^{++} and the calculated values.

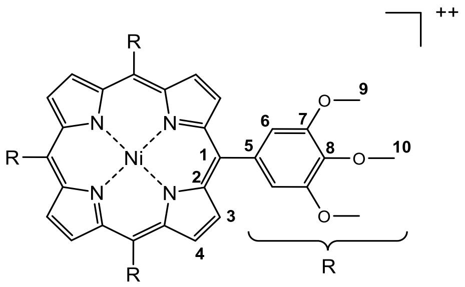

Numbering	experimental [ppm]	calculated*) [ppm]
C-1a)	148.2	147.0 (2C)
	145.3	146.7 (2C)
C-2a)	156.7	155.9 (2C)
	154.9	155.6 (2C)
		151.8 (2C)
		151.7 (2C)
C-3/4	137.6	138.1 (2C)
	135.6	137.7 (2C)
	132.0	134.2 (2C)
	131.1	133.3 (2C)
C-5	148.0	136.7 (2C)
	144.0	136.5 (2C)
	130.5	
	130.0	
C-6	108.8	113.2 (2C)
	108.7	113.1 (2C)
	108.5	112.7 (2C)
	108.3	112.2 (2C)
C-7/8	153.3	159.3 (*C*-7, 2C)
	153.0	159.2 (*C*-7, 2C)
	152.9	156.9 (*C*-7, 2C)
	139.2	156.7 (*C*-7, 2C)
	139.1	158.1 (*C*-8, 2C)
		157.8 (*C*-8, 2C)
C-9/10	55.1-61.9	59.0-68.1

a) Experimental assignment based on NMR-spectra not conclusive. C-numbering assigned based on calculation results.
*) PBE/def2-SVP//TPSS/pcSseg-2

Table S14. Comparison of the experimental ^{1}H shifts of the dication $\mathbf{9}^{++}$ and the calculated values for a dication with and without TFA anion present.

a)

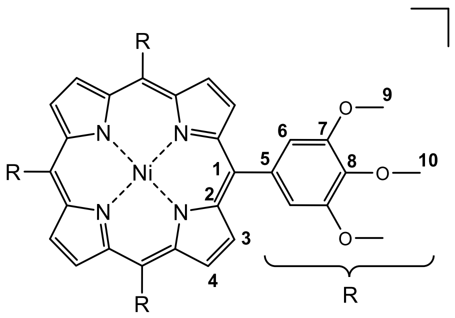

b)

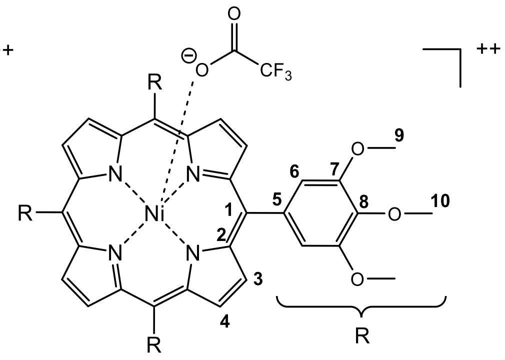

numbering	experimental [ppm]	calculated[a*)] [ppm]	calculated[b*)] [ppm]
o-phenyl-*H*	7.06 (2H)	6.54 (2H)	6.61(2H)
	6.68 (4H)	6.42 (2H)	6.53(2H)
	6.63 (2H)	6.33 (2H)	6.49(2H)
		6.22 (2H)	6.44(2H)
pyrrole-*H*	7.05 (2H)	7.21 (2H)	7.39(2H)
	6.99 (2H)	7.11 (2H)	7.29(2H)
	6.61 (2H)	6.60 (2H)	6.99(2H)
	6.34 (2H)	6.50 (2H)	6.48(2H)
m-methoxy-*H*	3.87-3.99	3.79-4.70[c)]	3.46-4.41[c)]
p-methoxy-*H*	3.87-3.99	3.62-4.70[c)]	3.62-4.99 [c)]

a) Dication $\mathbf{9}^{++}$
b) Dication $\mathbf{9}^{++}$ with one axial coordinated TFA anion at the Ni center
c) note that there is almost free rotation of the methoxy groups, however, the calculations refer to a single conformation including H-bonds
*) PBE/def2-SVP//TPSS/pcSseg-2

References

(1) Peters, M. K.; Röhricht, F.; Näther, C.; Herges, R. One-Pot Approach to Chlorins, Isobacteriochlorins, Bacteriochlorins, and Pyrrocorphins. *Org. Lett.* **2018**, *20*, 7879–7883. DOI: 10.1021/acs.orglett.8b03433.

(2) Ahn, H. M.; Bae, J. M.; Kim, M. J.; Bok, K. H.; Jeong, H. Y.; Lee, S. J.; Kim, C. Synthesis, Characterization, and Efficient Catalytic Activities of a Nickel(II) Porphyrin: Remarkable Solvent and Substrate Effects on Participation of Multiple Active Oxidants. *Chem. Eur. J.* **2017**, *23*, 11969–11976. DOI: 10.1002/chem.201702750.

(3) Balch, A. L.; Chan, Y. W. A complex containing a nickel-oxygen unit at the center of a porphyrin. The x-ray crystal and molecular structure of the nickel(II) complex of octaethylporphyrin N-oxide dianion. *Inorg. Chim. Acta* **1986**, *115*, L45-L46. DOI: 10.1016/S0020-1693(00)84399-6.

(4) Tsurumaki, H.; Watanabe, Y.; Morishima, I. Three Isoelectronic Structures of One-Electron-Oxidized Nickel Porphyrins. *Inorg. Chem.* **1994**, *33*, 4186–4188. DOI: 10.1021/ic00096a050.

(5) Glotz, G.; Knaipp, K.; Maier, M. S.; Hüll, K.; Novak, A.; Kelterer, A.-M.; Griebenow, T.; Herges, R.; Trauner, D.; Gescheidt, G. To Isomerize or not to Isomerize? E /Z Isomers of Cyclic Azobenzene Derivatives and their Reactivity upon One-Electron Reduction. *Chem. Eur. J.* **2023**, e202300146. DOI: 10.1002/chem.202300146.

(6) Weigend, F.; Ahlrichs, R. Balanced basis sets of split valence, triple zeta valence and quadruple zeta valence quality for H to Rn: Design and assessment of accuracy. *Phys. Chem. Phys.* **2005**, *7*, 3297–3305. DOI: 10.1039/B508541A.

(7) Weigend, F. Accurate Coulomb-fitting basis sets for H to Rn. *Phys. Chem. Phys.* **2006**, *8*, 1057–1065. DOI: 10.1039/B515623H.

(8) Perdew, J. P.; Ernzerhof, M.; Burke, K. Rationale for mixing exact exchange with density functional approximations. *J. Chem. Phys.* **1996**, *105*, 9982–9985. DOI: 10.1063/1.472933.
(9) Neese, F.; Wennmohs, F.; Becker, U.; Riplinger, C. The ORCA quantum chemistry program package. *J. Chem. Phys.* **2020**, *152*, 224108. DOI: 10.1063/5.0004608.
(10) Kingsbury, C. J.; Senge, M. O. The shape of porphyrins. *Coordination Chemistry Reviews* **2021**, *431*, 213760. DOI: 10.1016/j.ccr.2020.213760.
(11) Jentzen, W.; Song, X.-Z.; Shelnutt, J. A. Structural Characterization of Synthetic and Protein-Bound Porphyrins in Terms of the Lowest-Frequency Normal Coordinates of the Macrocycle. *J. Phys. Chem. B* **1997**, *101* (9), 1684–1699. DOI: 10.1021/jp963142h.
(12) Stephens, P. J.; Devlin, F. J.; Chabalowski, C. F.; Frisch, M. J. Ab Initio Calculation of Vibrational Absorption and Circular Dichroism Spectra Using Density Functional Force Fields. *J. Phys. Chem.* **1994**, *98* (45), 11623–11627. DOI: 10.1021/j100096a001.
(13) Geuenich, D.; Hess, K.; Köhler, F.; Herges, R. Anisotropy of the induced current density (ACID), a general method to quantify and visualize electronic delocalization. *Chemical reviews* **2005**, *105* (10), 3758–3772. DOI: 10.1021/cr0300901.
(14) Rassolov, V. A.; Ratner, M. A.; Pople, J. A.; Redfern, P. C.; Curtiss, L. A. 6-31G* basis set for third-row atoms. *J Comput Chem* **2001**, *22* (9), 976–984. DOI: 10.1002/jcc.1058.
(15) Rassolov, V. A.; Pople, J. A.; Ratner, M. A.; Windus, T. L. 6-31G* basis set for atoms K through Zn. *The Journal of Chemical Physics* **1998**, *109* (4), 1223–1229. DOI: 10.1063/1.476673.
(16) Blaudeau, J.-P.; McGrath, M. P.; Curtiss, L. A.; Radom, L. Extension of Gaussian-2 (G2) theory to molecules containing third-row atoms K and Ca. *The Journal of Chemical Physics* **1997**, *107* (13), 5016–5021. DOI: 10.1063/1.474865.
(17) Binning, R. C.; Curtiss, L. A. Compact contracted basis sets for third-row atoms: Ga–Kr. *J Comput Chem* **1990**, *11* (10), 1206–1216. DOI: 10.1002/jcc.540111013.
(18) Francl, M. M.; Pietro, W. J.; Hehre, W. J.; Binkley, J. S.; Gordon, M. S.; DeFrees, D. J.; Pople, J. A. Self-consistent molecular orbital methods. XXIII. A polarization-type basis set for second-row elements. *The Journal of Chemical Physics* **1982**, *77* (7), 3654–3665. DOI: 10.1063/1.444267.
(19) Gordon, M. S. The isomers of silacyclopropane. *Chemical Physics Letters* **1980**, *76* (1), 163–168. DOI: 10.1016/0009-2614(80)80628-2.
(20) Hariharan, P. C.; Pople, J. A. Accuracy of AH n equilibrium geometries by single determinant molecular orbital theory. *Molecular Physics* **1974**, *27* (1), 209–214. DOI: 10.1080/00268977400100171.
(21) Hariharan, P. C.; Pople, J. A. The influence of polarization functions on molecular orbital hydrogenation energies. *Theoret. Chim. Acta* **1973**, *28* (3), 213–222. DOI: 10.1007/BF00533485.
(22) Hehre, W. J.; Ditchfield, R.; Pople, J. A. Self—Consistent Molecular Orbital Methods. XII. Further Extensions of Gaussian—Type Basis Sets for Use in Molecular Orbital Studies of Organic Molecules. *The Journal of Chemical Physics* **1972**, *56* (5), 2257–2261. DOI: 10.1063/1.1677527.
(23) Ditchfield, R.; Hehre, W. J.; Pople, J. A. Self-Consistent Molecular-Orbital Methods. IX. An Extended Gaussian-Type Basis for Molecular-Orbital Studies of Organic Molecules. *The Journal of Chemical Physics* **1971**, *54* (2), 724–728. DOI: 10.1063/1.1674902.
(24) Gaussian 16, Revision A.03, Frisch, M. J.; Trucks, G. W.; Schlegel, H. B.; Scuseria, G. E.; Robb, M. A.; Cheeseman, J. R.; Scalmani, G.; Barone, V.; Petersson, G. A.; Nakatsuji, H.; Li, X.; Caricato, M.; Marenich, A. V.; Bloino, J.; Janesko, B. G.; Gomperts, R.; Mennucci, B.; Hratchian, H. P.; Ortiz, J. V.; Izmaylov, A. F.; Sonnenberg, J. L.; Williams-Young, D.; Ding, F.; Lipparini, F.; Egidi, F.; Goings, J.; Peng, B.; Petrone, A.; Henderson, T.; Ranasinghe, D.; Zakrzewski, V. G.; Gao, J.; Rega, N.; Zheng, G.; Liang, W.; Hada, M.; Ehara, M.; Toyota, K.; Fukuda, R.; Hasegawa, J.; Ishida, M.; Nakajima, T.; Honda, Y.; Kitao, O.; Nakai, H.; Vreven, T.; Throssell, K.; Montgomery, J. A., Jr.; Peralta, J. E.; Ogliaro, F.; Bearpark, M. J.; Heyd, J. J.; Brothers, E. N.; Kudin, K. N.; Staroverov, V. N.; Keith, T. A.; Kobayashi, R.; Normand, J.; Raghavachari, K.; Rendell, A. P.; Burant, J. C.; Iyengar, S. S.; Tomasi, J.; Cossi, M.; Millam, J. M.; Klene, M.; Adamo, C.; Cammi, R.; Ochterski, J. W.; Martin, R. L.; Morokuma, K.; Farkas, O.; Foresman, J. B.; Fox, D. J. Gaussian Inc., Wallingford CT, 2016.
(25) Zhao, Y.; Truhlar, D. G. A new local density functional for main-group thermochemistry, transition metal bonding, thermochemical kinetics, and noncovalent interactions. *The Journal of Chemical Physics* **2006**, *125* (19), 194101. DOI: 10.1063/1.2370993.
(26) Stoychev, G. L.; Auer, A. A.; Izsák, R.; Neese, F. Self-Consistent Field Calculation of Nuclear Magnetic Resonance Chemical Shielding Constants Using Gauge-Including Atomic Orbitals and Approximate Two-Electron Integrals. *Journal of chemical theory and computation* **2018**, *14* (2), 619–637. DOI: 10.1021/acs.jctc.7b01006. Published Online: Jan. 25, 2018.
(27) Tao, J.; Perdew, J. P.; Staroverov, V. N.; Scuseria, G. E. Climbing the density functional ladder: nonempirical meta-generalized gradient approximation designed for molecules and solids. *Physical review letters* **2003**, *91* (14), 146401. DOI: 10.1103/PhysRevLett.91.146401. Published Online: Sep. 30, 2003.

(28) Lu, T.; Chen, F. Multiwfn: a multifunctional wavefunction analyzer. *J Comput Chem* **2012**, *33* (5), 580–592. DOI: 10.1002/jcc.22885. Published Online: Dec. 8, 2011.
